# A novel mesh processing based technique for 3D plant analysis

**DOI:** 10.1186/1471-2229-12-63

**Published:** 2012-05-03

**Authors:** Anthony Paproki, Xavier Sirault, Scott Berry, Robert Furbank, Jurgen Fripp

**Affiliations:** 1 The Australian e-Health Research Centre, CSIRO ICT Centre, Australia; 2 The High-Resolution Plant Phenomics Centre, CSIRO Plant Industry, Australia

## Abstract

**Background:**

In recent years, imaging based, automated, non-invasive, and non-destructive high-throughput plant phenotyping platforms have become popular tools for plant biology, underpinning the field of plant phenomics. Such platforms acquire and record large amounts of raw data that must be accurately and robustly calibrated, reconstructed, and analysed, requiring the development of sophisticated image understanding and quantification algorithms. The raw data can be processed in different ways, and the past few years have seen the emergence of two main approaches: 2D image processing and 3D mesh processing algorithms. Direct image quantification methods (usually 2D) dominate the current literature due to comparative simplicity. However, 3D mesh analysis provides the tremendous potential to accurately estimate specific morphological features cross-sectionally and monitor them over-time.

**Result:**

In this paper, we present a novel 3D mesh based technique developed for temporal high-throughput plant phenomics and perform initial tests for the analysis of *Gossypium hirsutum* vegetative growth. Based on plant meshes previously reconstructed from multi-view images, the methodology involves several stages, including morphological mesh segmentation, phenotypic parameters estimation, and plant organs tracking over time. The initial study focuses on presenting and validating the accuracy of the methodology on dicotyledons such as cotton but we believe the approach will be more broadly applicable. This study involved applying our technique to a set of six *Gossypium hirsutum* (cotton) plants studied over four time-points. Manual measurements, performed for each plant at every time-point, were used to assess the accuracy of our pipeline and quantify the error on the morphological parameters estimated.

**Conclusion:**

By directly comparing our automated mesh based quantitative data with manual measurements of individual stem height, leaf width and leaf length, we obtained the mean absolute errors of 9.34%, 5.75%, 8.78%, and correlation coefficients 0.88, 0.96, and 0.95 respectively. The temporal matching of leaves was accurate in 95% of the cases and the average execution time required to analyse a plant over four time-points was 4.9 minutes. The mesh processing based methodology is thus considered suitable for quantitative 4D monitoring of plant phenotypic features.

## Background

In the coming decades, it is expected that mankind will need to double the quantity of food and biofuel produced in order to meet global demand [1]. To achieve this with existing resources, new plant characteristics need to be identified, quantified, and bred to obtain more productive plant varieties within existing environments. This will require a greater understanding of how the genetic make-up of plants determines their phenotype (visible traits) in high resolution and in high throughput. Performing plant phenomics involves screening large germplasm collections to facilitate the discovery of new interesting traits (*forward phenomics*), and analysing known phenotypic data in order to uncover the genes involved in their evolution and use these genes in plant breeding (*reverse phenomics*) [1]. Investigated plants are usually grown in thoroughly controlled conditions (growth chambers or glasshouses) and subjected to different environmental conditions and stresses (e.g. drought, salt, heat, etc.) with the primary aim of monitoring their phenotypic response using various measurements [2,3].

Common plant morphological traits of interest include parameters such as main stem height, size and inclination, petiole length and initiation angle, and leaf width, length, inclination, thickness, area, and biomass [1-4]. The usual procedure to collect these data consists of many laborious manual measurements, often requiring destructive harvests and thus multiple replicates of individual plant genotypes or varieties to allow successive harvests over time. A typical manual phenotypic analysis of 200 plants (daily objective) would require approximately 100 man-hours of work (≃ 30 minutes per plant depending on the size and complexity), which is impractical. In light of the importance of gene discovery and agricultural crop improvement, the development of solutions to automate such a tedious task is imperative.

High-throughput plant phenotyping aims to extend the standard approach by growing, measuring and analysing temporally thousands of plants [5]. In recent years, the plant phenotyping research has seen the emergence of high-throughput plant screening facilities [1,6]; however, few image and mesh processing solutions are available to analyse the large amount of data captured and extract yield determinants (i.e. plant, leaf, or root characteristics). Among existing solutions, PHENOPSIS [7] and GROWSCREEN [8,9], provide 2D image-processing based semi-automated solutions for leaf phenotyping (leaf width, length, area, and perimeter) and root data monitoring (number of roots, root area, and growth rate). LAMINA [10], another 2D-image based tool for leaf shape and size probing proposes a leaf analysis for various plant species. Recent image-processing solutions, such as TraitMill [11] and HTPheno [12], provide a more general plant analysis and measure information such as plant height, width, centre of gravity, projected area and bio-volume, and provide colorimetric analysis (e.g. greenness-differences between plants). Due to the importance of rice as a primary food resource, image-based solutions for rice phenotyping have been developed [6,13] and involve the measurements of parameters such as grain size (length, width, and thickness), panicle length, and number of tillers. In the past 2 years, fully automated imaging techniques for the high-throughput investigation of plant root characteristics (yield determinants) have been developed [14-16] to analyse non-destructively phenotypic traits such as root average radius, area, maximum horizontal width, and length distribution.

The latest applications have introduced a third dimension to the plant analysis. Stereo-imaging and mesh processing based systems, such as GROWSCREEN 3D [17], the *3D imaging and RootReader3D software platform*[18], or the solution proposed in [19], have pioneered the explicit 3D analysis of leaves and roots, allowing more accurate measurements of leaf area, and extraction of additional volumetric data.

To date, the literature is distinctly dominated by 2D image-processing techniques for high-throughput phenotyping of plants [6-16]. The major limitation of these 2D solutions is the loss of crucial spatial and volumetric information (e.g. thickness, bending, rolling, orientation) when transposing available data from 3D to 2D. The recent introduction of new tools for plant analysis based on explicit 3D reconstructions [17-19] (as opposed to inferred 3D based analysis [20,21], widely used since the 1960’s) promises to increase potential of high-throughput studies in terms of accuracy and exhaustiveness of the measured features, but available three-dimensional solutions are currently focussed on a specific organ (e.g. leaves [17,19] or roots [18]), tailored to a particular image acquisition system [22], and tend to be qualitative (or applied) rather than providing quantitative information and estimates of accuracy. Hence, a clear need exists for a more generalised plant analysis based on increasingly explicit 3D models and in which the reliability of the measurements is questioned and quantitatively assessed.

In this paper, we present a novel mesh-based technique developed for the high-throughput 3D analysis of plant aerial-parts. A focus is made on the feasibility of accurately extracting plant phenotypic parameters from a 3D mesh acquired for the dicotyledonous crop cotton. In this initial study, meshes were reconstructed using a low cost commercial 3D reconstruction system [23]. The proposed methodology aims at a non-exhaustive, accurate, cross-sectional (observation of a representative subset of a population at a fixed time-point), and temporal investigation of the plant macroscopic phenotype. This requires advanced features such as plant mesh morphological segmentation [24,25], accurate plant data extraction [26], and plant organs tracking over-time. The mesh based methodology was tested on plant meshes reconstructed [23,27] for a set of six plants studied at four time-points (i.e. 6×4 = 24 plant meshes).

## Methods

**Figure 1:**
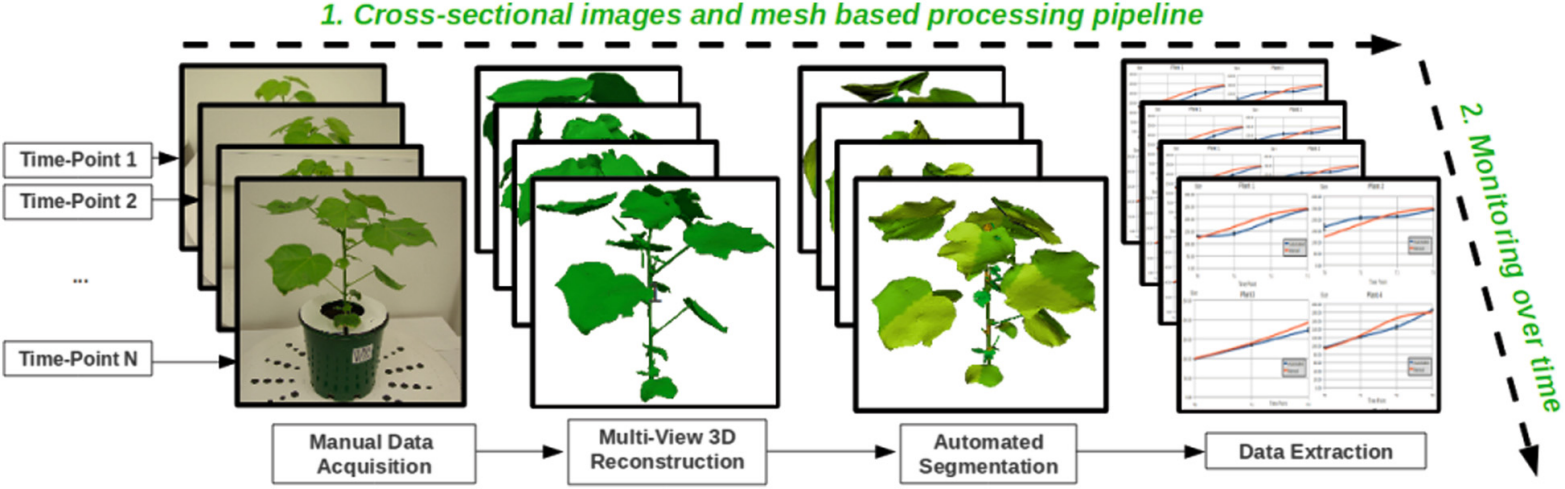
**Prototype plant analysis pipeline.** The horizontal axis **(1)** illustrates our prototype cross-sectional (observation of a representative subset of a population at a specific time-point) pipeline which is divided into 4 main stages: plant image acquisition, surface mesh reconstruction, morphological mesh segmentation (see Figure 2), and plant phenotypic parameters extraction. Monitoring the phenotypic parameters over-time **(2)** involves repeating the cross-sectional pipeline throughout all the time-points available and analysing the variations in the plant phenotype (the temporal pipeline is detailed on Figure 3).

To investigate the feasibility of a mesh based processing pipeline for the 3D analysis of plants, we initially developed the prototype cross-sectional pipeline described on Figure 1. The following sections propose a brief presentation of the image acquisition and plant mesh reconstruction steps, and detailed descriptions of the mesh segmentation, temporal phenotypic analysis, and validation scheme.

**Figure 2:**
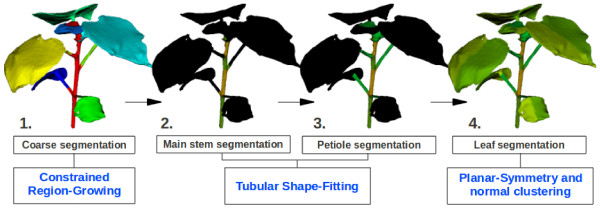
**Automated segmentation pipeline.** Illustration of the 4 successive steps of the segmentation pipeline: **(1)** coarse segmentation - takes as input the non segmented plant mesh and provides as output a plant mesh roughly segmented (*1* region for the main stem (red) and *N* regions for the associated petioles and leaves (other colours)), **(2)** precise stem segmentation - takes as input the main stem previously segmented (region *M*) and partitions it into several internodes, **(3)** petiole segmentation - takes as input a given associated leaf and petiole (from step 1) and separates them into two distinct regions (process repeated for all the associated leaves/petioles), and **(4)** leaf segmentation - takes as input a given separated leaf and segments it into adaxial/abaxial surfaces and left/right parts (process repeated for all the leaves).

**Figure 3:**
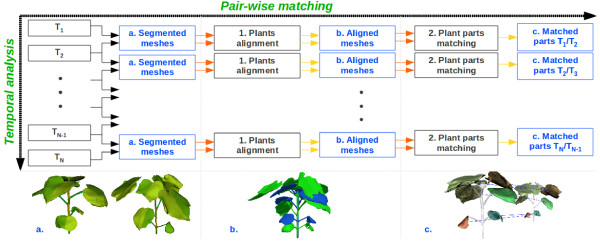
**Temporal analysis pipeline.**The full temporal analysis involves repeating the same pair-wise matching pipeline throughout all the time points. The pair-wise matching (horizontal axis) is constituted of two steps: **(1)** a plant alignment, that takes as inputs the segmented plant meshes for the time-points *T*_*x*_and *T*_*x* + 1_(see **(a)**) and rigidly aligns the mesh from time-point *T*_*x*_with the mesh from time-point *T*_*x* + 1_(see resulting mesh *T*_*x*_in blue on **(b)**), and **(2)** a plant parts matching that matches the different plant organs using the aligned meshes (see example of matched leaves on **(c)**). The full temporal matching (vertical axis) consists of repeating this pair-wise matching throughout all the time-points in order to obtain the mapping of the different organs of the plant over-time.

### Plant material

The prototype study involved acquiring and processing images for an initial set of six *Gossypium hirsutum* plants studied over four time-points. Manual measurements, performed by X.S and S.B for each plant and each time-point, were used to validate the accuracy and quantify the error on the mesh-based phenotypic data estimation. The first three time-points involved measuring invasively (but non-destructively) parameters such as main stem height, leaf width and leaf length using a measuring tape. For the last time-point, measurements were collected after destructive harvest in order to optimise their precision. The petioles and leaves were cut from the main stem, laid flat on a table and carefully measured. Overall, a set of 384 measurements was manually collected (24 main stem height measurements, 180 leaf width measurements, and 180 leaf length measurements).

### Plant data acquisition and mesh reconstruction

A manual data capture process similar to that described in [12] was used to collect multiple plant images from different viewing angles using a high-resolution Pentax K10 SLR camera with a sigma 20-40mm aspherical lens. Each cotton plant pot was placed at the centre of a rotating tray. The camera was fixed on a tripod during all the acquisition process. The rotating tray was manually turned and pictures were taken at each rotation angle (every $\frac{360}{64}$ degree). The acquisition process completed, 64 images were available (per plant and time-point) for the multi-view 3D reconstruction. An example of acquired plant image is shown on Figure 1, the image resolution was 3872x2592 pixels (≃ 10 Megapixels).

Plant 3D models (meshes) were created from the high-resolution images using 3DSOM, a commercial 3D digitisation software [23]. The number of polygons constituting the reconstructed meshes fluctuated between 120000 and 270000.

The acquisition and mesh generation are not the primary focus of the current paper, however we acknowledge the “semi-automated” steps involved. An automated image acquisition platform [28] and a mesh reconstruction algorithm (based on [29-31]) are under development and will allow full automation for future experiments.

### Automated plant mesh segmentation

The identification of different plant organs is a critical stage in performing mesh-based plant phenotyping and has proven problematic with 2D based image analysis solutions [1]. To complete this task, we developed an advanced mesh segmentation algorithm that partitions the plant mesh into morphological regions.

Mesh segmentation algorithms involve assigning a unique value (called a label) to all the points of the mesh (called vertices) that belong to the same region. A surface mesh is constituted of triangles that link the vertices together through their edges. Two vertices are said to be topologically connected (neighbours) if they share the edge of a triangle. Finally, a vertex comprises a normal vector equal to the average of the normal vectors of the neighbouring triangles.

Due to the complex and irregular morphology of plants, no generic mesh segmentation algorithm [24,25] is accurate and robust enough to identify the different plant parts (main stem, petioles, leaves). This paper introduces a “hybrid” segmentation pipeline that overcomes the morphological shape differences between cotton plants and various reconstruction inconsistencies due to occlusions (the most common being missing petioles, as they are occluded by the leaves in the images used by the reconstruction scheme). Our automated segmentation pipeline, illustrated in Figure 2, is constituted of 4 successive steps: a coarse segmentation, a stem segmentation, petioles segmentations, and leaves segmentations. All the operations described in the next paragraphs are fully automatic and do not require any manual input [28].

#### Step 1: Coarse segmentation

The purpose of this first step is to partition the plant into *n+1* coarse regions (with *n* = number of leaves), *one* for the main stem (region *M*) and *n* for the pairs of petioles and leaves (regions *N*_*i*_*i*=1,…,*n*). This is performed by a region-growing algorithm [24,32]. Region-growing algorithms start from a seed point (automatically selected based on prior criteria defined by the application) and gradually grow a region from neighbour to neighbour until a given criteria is met. Since the criteria to stop the growth of a region are user-defined, this generic approach is particularly convenient for coarse segmentations but often shows limitations when seeking accurate region delineation.

The scheme starts by defining a coarse region *M* as the main stem by fitting a curve *c*_*p*_ to the main stem from one extremity to the other and assigning to the region *M* all the vertices in a given planar radius of *c*_*p*_. Remaining vertices are classified into the *n* regions *N*_*i*_using a region-growing algorithm. The algorithm finds the first vertex that is not part of any region yet (at the start only one region is defined: *M*), uses it as seed point, and recursively grows a new region to all the eligible topological neighbours (creating a second region *N*_1_). A neighbour is eligible if it does not belong to *M* or any of the regions *N*_*i*_already created. The region stops to grow when there is no eligible neighbour remaining, i.e. all neighbours are labelled. The algorithm iterates through all the vertices of the mesh and grows a new region *N*_*i*_each time it finds a vertex that does not belong to any of the regions *M* or *N*_*i*_ already created (new seed point). This scheme is robust to reconstruction issues such as holes in the mesh or detached mesh pieces, as a vertex does not need to be connected to the main mesh to become a seed. A typical result of this pass is shown in Figure 2.1.

#### Step 2: Main stem segmentation

The second segmentation step is based on a primitive fitting segmentation approach [33,34] that aims at refining the rough stem segmentation and partitioning the stem into different internodes (using the previously extracted region *M*). Primitive fitting algorithms involve finding a given shape (chosen based on the mesh structure) in a complex mesh and considering that all the vertices within the registered shape belong to the same region. In this work, the tubular shape fitting algorithm involves finding the tube parameters that minimise the point to surface distance to the region *M*.

**Figure 4:**
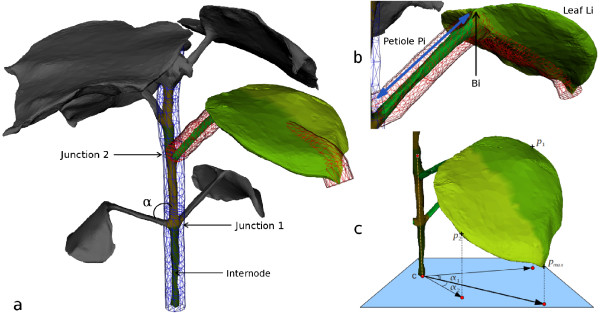
**Schematic illustration of the segmentation.(a)** shows the tubes used to segment the stem (blue) and one petiole (red). **(b)** illustrates the tube used to separate (segment) the petiole *P*_*i*_from the leaf *L*_*i*_. **(c)** illustrates the planar symmetry used to segment the leaf into two symmetric parts. In this particular case, the points *p*_1_and *p*_2_will belong to two different leaf regions as the angles *α*_1_and *α*_2_are signed differently .

The algorithm creates a tube around the curve *c*_*p*_(see previous section) and optimises its radius to tighten the shape around the main stem (see Figure 4.a). Vertices inside the final tube fitted are considered part of the main stem region. The vertices bordering the tube (i.e. junctions between the petioles and the main stem) are used to partition *M* into different regions. A stem internode goes from one junction to the other (see Figure 4.a). Typical stem segmentations are shown in Figures 2.2 and 4.a.

#### Step 3: Petiole segmentation

The petioles are also segmented (and separated) from the leaves in each regions *N*_*i*_ (created in the first step of the segmentation) using tubular fitting. For each petiole and associated leaf in the regions *N*_*i*_, we interpolate a curve along the petiole (using the local centre of mass of the vertices) and build a tube around it (see red tube on Figure 4.a/b). The tube follows the petiole and extends to the apex of the leaf. If we define *B*_*i*_ as the vertex outside the tube which is the closest to the main stem (leaf stalk), then all the vertices inside the tube which are closer to the main stem than *B*_*i*_ belong to the petiole region *P*_*i*_ (see Figure 4.b). Other vertices naturally belong to the leaf region *L*_*i*_. In the case of a missing petiole (detected by a non-topological connectivity to the main stem), this step is skipped, and the region is processed using Step 4. Figure 2.3 illustrates a typical plant mesh after the petiole segmentations.

#### Step 4: Leaf segmentation

**Figure 5:**
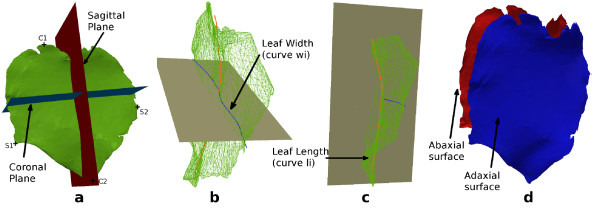
**Illustration of a leaf data extraction.(a)** shows the leaf sagittal and coronal planes, and the points *S*_*i*,1_, *S*_*i*,2_, *C*_*i*,1_, *C*_*i*,2_which are used to compute the leaf width and length, **(b)** shows, in blue, the projection (onto the coronal plane) of the line fitted to the shape of the leaf from *S*_*i*,1_to *S*_*i*,2_. This projected line length is the estimate of the leaf width. **(c)** shows, in orange, the projection (onto the sagittal plane) of the line fitted to the shape of the leaf from *C*_*i*,1_to *C*_*i*,2_. This projected line length is the estimate of the leaf length. **(d)** represents a leaf transversally sliced into abaxial and adaxial surfaces (using the vertices normal vector clustering algorithm).

The leaf segmentation algorithm aims at obtaining a *sagittal* (left and right parts) and *transversal* (adaxial and abaxial surfaces: see Figure 5.d) segmentation. It has been designed to be robust to numerous natural leaf shape variations (bending, changes over-time) and erroneous leaf reconstructions due to occlusions during the 3D reconstruction (e.g. abnormal leaf thickness, leaves stuck together). A high accuracy of this stage is crucial for leaf width, length, area, and average thickness estimation. The proposed solution is based on two properties common to all the studied leaves: the symmetry and the vertices normal vector distribution (mainly pointing in two directions: away from the leaf adaxial or abaxial surface).

For the sagittal segmentation, a 2D-symmetry based algorithm was found to be the most robust, accurate, and computationally efficient. It is obtained by projecting the vertices of the leaf onto the plane having the main stem axis as normal and comparing the sign of the angle between the vector going from the main stem (*c*_*p*_) to the apex and the vector going from *c*_*p*_to the considered vertex. The region to which a vertex belongs to depends on the sign of the angle (*α*_1_ and *α*_2_ in Figure 4.c). An illustration of this process is provided in Figure 4.c.

The transversal segmentation involves using a normal clustering algorithm that starts by computing $\vec{V_{n_{i}}}$ as the average normal vector for the whole leaf *L*_*i*_($\vec{V_{n_{i}}}$ will naturally point away from adaxial or abaxial surface). A first pass will sort the leaf vertices into two different regions (adaxial or abaxial) depending on the angle formed by their normal vector and $\vec{V_{n_{i}}}$ (higher or lower than $\frac{\Pi }{2}$). We then recompute $\vec{V_{n_{i}}}$ using only the normal vectors of the vertices belonging to one of the two created regions and repeat the sorting process using the updated $\vec{V_{n_{i}}}$. This scheme is repeated until a natural convergence occurs (i.e. $\vec{V_{n_{i}}}$ does not change between two iterations).

In the case where two leaves were merged together due to occlusion issues in the 3D reconstruction, the algorithm detects a largely greater volume, splits the region *L*_*i*_ into two regions, and performs the normal leaf segmentation on each region. To create the two regions, the algorithm detects the points *f*_*i*,1_ and *f*_*i*,2_ which are the furthest apart in the region *L*_*i*_, computes the centroid *f*_*c* *i*_of these two points, and uses as split plane the plane defined with *f*_*c* *i*_ as origin and the normalised vector from *f*_*c* *i*_to *f*_*i*,2_ as normal. The mesh segmentation after this step is shown on Figure 2.4.

### Phenotypic parameters of interest

For phenotypic analysis, important parameters are main stem height, size and inclination, petiole length and initiation angle, and leaf width, length, area, and inclination. This section presents the process used to extract these parameters from the segmented plant mesh, and focuses in particular on the leaf parameters, which are crucial indicators of the level of stress to which the plant is subjected to [2,3].

#### Main stem

The main stem *height* can be expressed as the height difference between the highest and lowest vertices of the region *M*. The normalised vector between these two vertices defines the main stem axis and the angle between this axis and the coordinates system up-vector gives the inclination of the main stem. In this work the main stem *length* is defined as the length of the curve *c*_*p*_fitted to the main stem.

#### Petiole

If *c*_*i*_ is a curve interpolated along the petiole *P*_*i*_(using local vertices centre of mass), the length of the petiole can then be expressed as the length of *c*_*i*_. In addition, if *l*_*i*_ and *h*_*i*_ denote the points of *c*_*i*_ that are the closest to *c*_*p*_ and the highest respectively, then the angle *α* between the main stem axis and the vector $\vec{l_{i}h_{i}}$ defines the petiole initiation angle (see *α*on Figure 4.a).

#### Leaf blade

For each segmented leaf *L*_*i*_, we define *L*_*ci*_ as the centroid of the leaf, $\vec{u_{i,1}}$ as the average of the vectors going from *L*_*ci*_ to the vertices belonging to the *right* part of the leaf, and $\vec{u_{i,2}}$ as the vector going from *L*_*c* *i*_ to the tip of the leaf. Let $\pi_{i,1}=(Lc_{i},\vec{u_{i,1}})$ and $\pi_{i,2}=(Lc_{i},\vec{u_{i,2}})$ define the leaf sagittal and coronal planes (in which *L*_*c* *i*_and $\vec{u_{i,x}}$ are the origin and normal of the plane _*π* *i*,*x*_) as displayed in Figure 5.a. Let *S*_*i*,1_and *S*_*i*,2_ (*resp.C*_*i*,1_ and *C*_*i*,2_) be the points on each side of *π*_*i*,1_ (*resp.π*_*i*,2_) that maximise the distance to *π*_*i*,1_(*resp.π*_*i*,2_) as illustrated on Figure 5.b/c.

To estimate the leaf width (*resp.* length), we compute the length of the curve *w*_*i*_ (resp. *l*_*i*_) interpolated to the leaf shape from *S*_*i*,1_to *S*_*i*,2_ (*resp.C*_*i*,1_ to *C*_*i*,2_) and projected onto *π*_*i*,2_(*resp.π*_*i*,1_) (to remove additional transversal length). Illustrations are provided in Figure 5.b/c. The projection of $\vec{C_{i,1}C_{i,2}}$ onto *π*_*i*,1_ is used as leaf axis. The angle between this axis and the main stem axis gives the leaf inclination. The leaf area can be estimated by averaging the areas of the adaxial and abaxial surfaces (see Figure 5.c), which are computed by summing the area of the triangles composing them. The leaf thickness can be estimated by averaging the distance between each vertex of the adaxial surface to the closest vertex on the abaxial surface.

### Analysis over-time

This step of the pipeline involved monitoring the variations of the estimated plant parameters over time. Even though this is a straightforward process for stem height monitoring, the temporal petiole and leaf parameters analysis requires an efficient matching algorithm that tracks the different plant parts over time (orientation and size of the leaves change over time as a result of variations in growing conditions, making it difficult to find robust descriptors).

To perform this task, we developed the pipeline presented on Figure 3 which is based on the assumption that a plant organ position does not vary much between two close imaging dates. We apply the same “pairwise matching” algorithm (horizontal axis on Figure 3) throughout all the available time-points (i.e. matching of *T*_1_ and *T*_2_, of *T*_2_ and *T*_3_, …, of *T*_*N*−1_ and *T*_*N*_. See vertical axis on Figure 3) in order to obtain the sequences of leaves and petioles. The pair-wise scheme is divided into two main steps: an *alignment* of the two plants and a *parts matching* algorithm.

#### Plants alignment

The plant at *T*_*x*_ is rigidly aligned with the plant at *T*_*x* + 1_using a translation and a rotation around its main stem axis. The translation is performed using the vector going from centre of the plant at *T*_*x*_(lowest point of the main stem region) to the centre of the plant at *T*_*x* + 1_. The rotation is performed using the angle *α* that minimises the metric *m*_*α*_ defined in Eq. (1). In this equation $Lc_{T_{x},i}$, stands for the centroid of the leaf *i*, and $\Psi_{T_{x}}$ for the number of leaves for the plant at time-point *T*_*x*_, and $D(Lc_{T_{x},i},Lc_{T_{x+1},j})$ expresses the 3D Euclidean distance between $Lc_{T_{x},i}$ and $Lc_{T_{x+1},j}$. An example of the alignment is shown in Figure 3.b.

(1) $$m_{\alpha }(T_{x},T_{x+1})=\overset{i\leq \Psi_{T_{x}}}{\underset{i=1}{\sum }}D_{\mathrm{opt}}(Lc_{T_{x},i})$$

(2) $$\mathrm{where}D_{\mathrm{opt}}(Lc_{T_{x},i})=\underset{1\leq j\leq \Psi_{T_{x+1}}}{\min}(D(Lc_{T_{x},i},Lc_{T_{x+1},j}))$$

#### Plant parts matching

##### Internodes matching:

**Figure 6:**
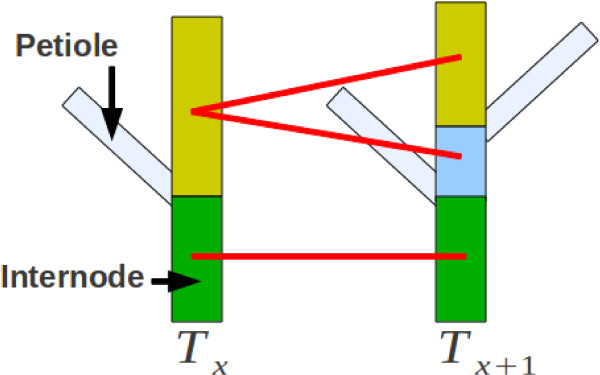
**Illustration of the process to match internodes.** An internode from the plant at *T*_*x*_can be matched with multiple internodes from the plant at *T*_*x* + 1_in the case of a petiole appearance between the two time-points.

This step aims at matching the different internodes of a plant between two time-points. Petioles may grow between two time-points, and thus, split an internode of the plant at time-point *T*_*x*_into two parts, meaning that an internode from the plant at time-point *T*_*x*_ can be matched with multiple internodes from the plant at *T*_*x* + 1_. For the two plants, we rank the internodes by normalised height, retrieve their lowest and highest boundaries and match two together when there is an overlap between their boundaries. Figure 6 shows a schematic illustration of the process.

##### Leaf blades and petioles matching:

Using two aligned plants, we match the different leaves and petioles of the plants by solving an assignment problem. We build an adjacency matrix (comporting $\Psi_{T_{x}}$ rows and $\Psi_{T_{x+1}}$ columns) such that at a given position (*ij*) in the matrix, we store the distance $D(Lc_{T_{x},i},Lc_{T_{x+1},j})$ between the centroids of the leaf *i* of the plant at *T*_*x*_ and of the leaf *j* of the plant at *T*_*x* + 1_. Since the plants are now aligned, two leaves are eligible for pair-wise correspondence only if they belong to a given angular range from each other, and we set to ∞ the distance between them in the adjacency matrix when this condition is not satisfied. The pair-wise matching is performed using a simplified version of the Hungarian algorithm [35] that minimises the sum of the distances between the paired leaves. The petioles linked to the paired leaves are paired at the same time.

After this step, the morphological parts of the plant are matched over time.

### Validation methodology

We were able to compute the relative error $\varepsilon_{a_{i},m_{i}}$ and the squared difference $\Delta_{a_{i},m_{i}}^{2}$ for a given automated measurement *a*_*i*_ with respect the manual measurement *m*_*i*_by applying Eq. (2) and (3):

(3) $$\varepsilon_{a_{i},m_{i}}=\frac{\left|a_{i}-m_{i}\right|}{m_{i}}$$

(4) $$\Delta_{a_{i},m_{i}}^{2}={\left(a_{i}-m_{i}\right)}^{2}$$

Let *S*_*a*_={*s*_*a*1_,…,*s*_*aΩ*_} and *S*_*m*_={*s*_*m*1_,…,*s*_*mΩ*_} denote the sets of automated and manual main stem height measurements (with *Ω* number of plants). Let also *W*_*a*_={*w*_*a*1_,…,*w*_*aΦ*_} and *W*_*m*_={*w*_*m*1_,…,*w*_*mΦ*_} denote the sets of automated and manual leaf width measurements (with *Φ* total number of leaves: 180). Using Eq. (2) and (3), we can then express the mean absolute percentage errors *E*_*s*_, *E*_*w*_, and the root mean square errors *RMSE*_*s*_, *RMSE*_*w*_ on the main stem height and leaf width measurements by:

(5) $$\begin{array}{cc} E_{s} & =\frac{100\times \overset{i\leq \Omega }{\underset{i=1}{\sum }}\varepsilon_{S_{a,i},S_{m,i}}}{\Omega }\mathrm{and}\\ \mathrm{RMS}E_{s} & =\sqrt{\frac{\overset{i\leq \Omega }{\underset{i=1}{\sum }}\Delta_{S_{a,i},S_{m,i}}^{2}}{\Omega }} \end{array}$$

(6) $$\begin{array}{cc} E_{w} & =\frac{100\times \overset{i\leq \Phi }{\underset{i=1}{\sum }}\varepsilon_{W_{a,i},W_{m,i}}}{\Phi }\mathrm{and}\\ \mathrm{RMS}E_{w} & =\sqrt{\frac{\overset{i\leq \Phi }{\underset{i=1}{\sum }}\Delta_{W_{a,i},W_{m,i}}^{2}}{\Phi }} \end{array}$$

A similar analysis allows to compute *E*_*l*_and *RMSE*_*l*_ for the leaf length measurements.

These errors were computed either using the whole datasets mentioned, or using the datasets trimmed from 10% of the outliers (5% of the best and worst relative errors). In addition, to be able to test the correlation between the automated and manual measurements, we calculated the *squared Pearson product-moment correlation coefficient* (^*R*2^) [36] and the *Intraclass Correlation Coefficient* (ICC - Two-ways random single measures) [37-39]. The closer the ^*R*2^ and ICC coefficients are to 1, the stronger the correlation between two measurements.

## Results

The results we obtained by applying our processing pipeline on the initial population of 6 *Gossypium hirsutum* plants studied over 4 time-points are presented bellow.

### Plant mesh segmentation

**Figure 7:**
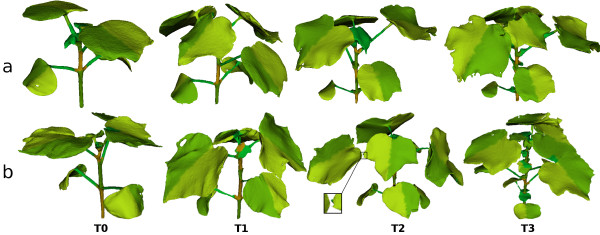
**Examples of plant segmentation results**Illustration of the segmentation of two *Gossypium hirsutum* cotton plant **(a)** and **(b)** studied over 4 time-points. As shown by the segmentation of the plant **b** at _*T*2_and _*T*3_the algorithm is robust to reconstruction issues such as floating mesh parts (typically leaves), and two leaves merges together.

As illustrated by the segmentation results displayed in Figure 7, the segmentation pipeline performs well and meets its targeted expectation which is the identification of the morphological parts of the plant. The algorithm has proved to be robust to the mesh abnormalities caused by occlusions during the reconstruction step, including holes in the mesh and plant parts detached from the main mesh (typically, a missing petiole, see magnification on Figure 7 b/T2) or stuck together (see Figure 7 b/T3). The accuracy of the final mesh partitioning is limited by the quality of the 3D reconstruction and irregularities in the sagittal segmentation of a leaf could appear if the leaf is considerably rotated with respect to the main stem.

### Phenotypic parameters estimation

**Figure 8:**
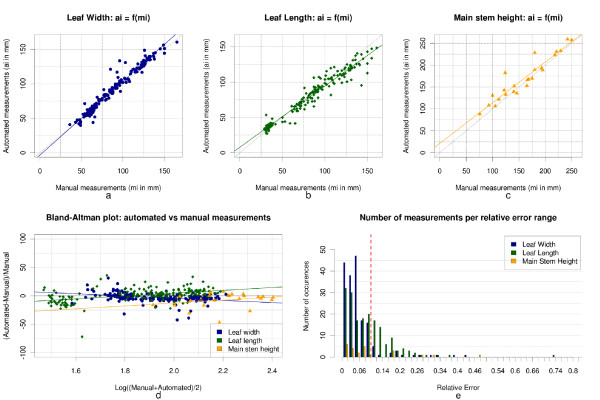
**Statistical analysis: Comparison of mesh-based measurements with manual ground-truthing(a)**, **(b)**, and **(c)** present scatter plots of the different phenotypic parameters evaluated by our pipeline against manual measurements. The *squared Pearson correlation* and *intra-class correlation* coefficients computed for the main stem height, leaf width, and leaf length measurements were $R_{s}^{2}≃0.887$, $R_{w}^{2}≃0.957$, $R_{l}^{2}≃0.948$, *ICC*_*s*_≃0.941, *ICC*_*w*_≃0.974, *ICC*_*l*_≃0.967, **(d)** is the Bland-Altman plot of our datasets (i.e. the relative error against logarithm of the mean of two measurements), **(e)** illustrates the distribution of the error for each measurement type. The dotted red line represents the 10% relative error.

Our methodology allowed us to perform 384 phenotypic measurements on our initial population of 24 *Gossypium hirsutum* plant meshes (24 main stem height measurements, and 180 measurements for both leaf width and length measurements). Overall, the described methodology estimates plant phenotypic parameters with sufficient accuracy and reproducibility to be used as a surrogate for manual or destructive phenotypic analysis (Table 1). We noted mean absolute percentage errors of _*E* *s*_≃9.34*%* (15.9 mm with an average stem height of 170.9 mm), _*E* *w*_≃5.75*%* (5.11 mm with an average leaf width of 88.9 mm), and _*E* *l*_≃8.78*%*(6.93 mm with an average leaf length of 78.9 mm) on the main stem height, and leaf width and leaf length measurements. The root mean square errors computed were *RMS*_*E* *s*_≃19.043 mm, *RMS*_*E* *w*_≃7.287 mm, and *RMS*_*E* *l*_≃9.707 mm. These values provide the average difference between the mesh based measurements and the direct measurements in millimetres. The Bland-Altman plot and the distribution of the relative error, presented in Figure 8.d/e, allow a more thorough analysis of the error and show that, even though most of the measurements were performed within an error range of [0*%*;10*%*] (see dotted red line on the Figure 8.e), many outliers remain in the analysis (vertical scattering on the Bland-Altman plot). The presence of outliers is caused by imprecision in the mesh segmentation and/or erroneous plant reconstructions due to occlusions during the 3D reconstruction. The scatter-plots and linear regressions displayed in Figure 8.a and 8.b allow to appreciate the strong correlations between the mesh-based and direct leaf measurements. The plotted point-clouds fall into slightly scattered lines and the linear regressions are approaching the targeted reference (i.e. *y*=*x*). The squared *Pearson* correlation and intraclass correlation coefficients $R_{w}^{2}≃0.957$, *IC*_*C* *w*_≃0.974, $R_{l}^{2}≃0.948$, and *IC*_*C* *l*_≃0.967 calculated on the leaf width and length measurements concord with our previous statement of strong correlations as they approach 1. Finally, although the plot of Figure 8.c shows a more scattered point-cloud for the main stem height measurements, the correlation coefficients found were $R_{s}^{2}≃0.887$ and *IC*_*C* *s*_≃0.941, which are acceptable precisions for our research.

**Table 1:** Main stem and leaf measurements analysis

**Comparison between the automated and manual measurements**									
	|*v*_*x*_|	*E*_*x*_	Range (mm)	*σ*_*x*_	*E*_*x*,10*%*_	*σ*_*x*,10*%*_	$R_{x}^{2}$	*ICC*_*x*_	*RMSE*_*x*_
Main Stem Height	24	9.34%	15.95	11.50%	7.29%	6.88%	0.887	0.941	19.043
Leaf Width	180	5.75%	5.11	6.40%	4.78%	3.20%	0.957	0.974	7.287
Leaf Length	180	8.78%	6.93	8.36%	7.92%	5.42%	0.948	0.967	9.707

In this table, we compare the values automatically extracted from the meshes with our direct measurements. |*v*_*x*_| is the cardinality of *v*_*x*_, *E*_*x*_ and *E*_*x*,10*%*_are respectively the non-trimmed and trimmed (10% extrema values removed) mean absolute percentage errors, and *σ*_*x*_ and *σ*_*x*,10*%*_ are respectively the non-trimmed and trimmed standard deviations. $R_{x}^{2}$ is the squared Pearson correlation coefficient, *ICC*_*x*_ is the intraclass correlation coefficient. *RMSE*_*x*_ is the Root Mean Square Error.

### Temporal analysis

**Figure 9:**
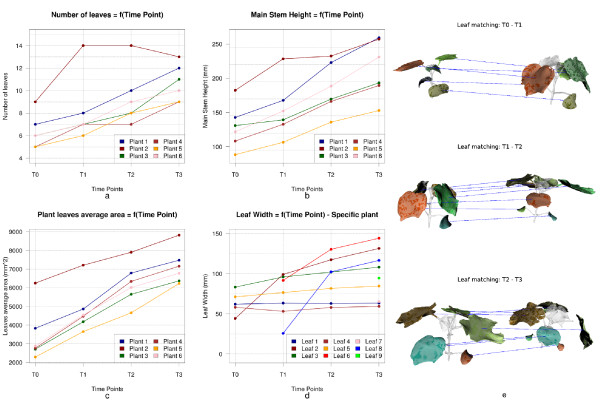
**Example of temporal analysis performed over 4 time-points(a)** presents the evolution of the number of leaves per plant, **(b)** illustrates the evolution of the main stem height for each plant in our initial set, **(c)** monitors the evolution of the average leaf area per plant, **(d)** monitor the evolution of the width of individual leaves for a given plant, and **(e)** illustrates the results of the pair-wise matching process for a given plant (for each pair-wise matching, the leaves matched are coloured similarly). These graphs illustrate an important contribution of our work, the temporal matching, as to date, no available solution allows to monitor the evolution of specific plant organs over-time while at the same providing a general analysis of the plant.

The automated temporal analysis of the plants was quite robust to the two major challenges: the growth of the plants over time and the changes in the topology and shape of the leaves over time. Correct matches of the different plant organs occurred in 95% of the cases (missing petioles were ignored). Illustrations of the results obtained by applying the pair-wise matching pipeline for a plant studied over 4 time-points are proposed in Figure 9.e. A dependency of the current matching scheme is that an organ needs to be accurately identified in order to be matched.

### Computational cost

The automated 3D analysis was performed on a standard computer equipped with a processor Intel Core 2 Duo E8300 (2.83GHz). The mesh analysis involved an average execution time of 4.9 minutes and a total of 29.3 minutes for the complete analysis of six plants over four time-points (with plant meshes decimated to 70000 triangles and without algorithm optimisations outside standard speeding techniques). Table 2 provides the full analysis of the computational cost of our algorithm. From Table 2 and Figure 9.a, we can denote that a higher the number of leaves will involve a slightly longer time to perform the analysis (due to repeated leaf segmentations and data computations, e.g. Plant 2). The time required to perform the full mesh-based analysis (3D reconstruction excluded) is faster than any manual method can be, and the performed analysis provides additional information on the evolution of the data over-time. Future work will involve paralleling different stages of the algorithm (such as step 2 and 3 of the segmentation, the leaves segmentations, or the computation of the metrics from Eq. (1) in the plant alignment) and utilising cluster and cloud computing technologies to access more important computational resources.

**Table 2:** Analysis of the computational cost (in minutes)

**Analysis of the computational cost (in minutes)**							
**Operation**	**Time-Point**	**Plant 1**	**Plant 2**	**Plant 3**	**Plant 4**	**Plant 5**	**Plant 6**
		T0	0.62	0.64	0.71	0.70	0.61	0.62
		T1	0.64	0.62	0.57	0.51	0.55	0.81
**Segmentation**								
	T2	0.71	0.69	0.52	0.68	0.65	0.60	
	T3	0.68	0.62	0.67	0.80	0.71	0.81	
	T0	0.075	0.065	0.082	0.073	0.074	0.078	
		T1	0.067	0.066	0.075	0.062	0.074	0.074
**Data extraction**								
	T2	0.072	0.068	0.061	0.063	0.068	0.067	
	T3	0.073	0.072	0.071	0.076	0.071	0.071	
**Temporal organs matching**		2.21	2.51	2.16	2.01	2.05	2.12	
**Complete mesh analysis**		4.97	5.19	4.65	4.77	4.67	5.06	

For the sake of consistency in the analysis of the CPU time (number of CPU cycles elapsed between the beginning and the end of an operation) required to run each step of the pipeline, we initially decimated all the plant meshes to 70000 triangles. The programs were run on a computer equipped with a processor Intel Core 2 Duo E8300 (2.83GHz). The segmentation steps were executed successively (non-threaded pipeline) and the results were obtained in less than 1 minute (< 50s) for every plant mesh. The data extraction steps were also executed successively, and the processing time required was negligible (< 0.082 min / < 5s per plant and per time-point). The CPU time required to run the plant parts matching for four time-points oscillated around 2 minutes (depending on the number of leaves). Operations of alignment and matching were executed successively. Note that the temporal pipeline includes the data extraction algorithm, as the plant data are required by the temporal pipeline. Finally the CPU time required to run the full mesh processing pipeline was around 5 minutes per plant. For a given plant, the segmentation of the different meshes were executed in parallel (4 time-points = 4 plant mesh segmentations) and followed by a serial temporal analysis.

### Additional results

Additional results are provided in the web-site associated with this paper [see Additional file 1].

## Discussion

As illustrated by Figure 9.a, 9.b, 9.c, and 9.d, that present a comparative study of the temporal evolution of phenotypic parameters for 6 *Gossypium hirsutum* plants, our methodology allows an accurate monitoring of the plants’ phenotypic traits over-time. By developing a hybrid mesh segmentation and analysis methodology for plant phenotyping, we have demonstrated that the automated temporal mesh-based analysis of the plant aerial part is feasible (from the temporal broad plant analysis to the evolution of individual organs).

Nevertheless, our initial study has several limitations which should be acknowledge and will lead to further investigation and development.

As of today, the pilot study was limited in terms of the exhaustiveness of the phenotypic parameters estimated, but the explicit 3D reconstruction and robust identification of the morphological parts of the plant allow estimation of a large number of parameters of interest to plant biologists not easily extracted from 2D images with existing software platforms (accurate leaf area and biovolume rather than projected area, growth of individual leaves, organ quantification over time, leaf number / phyllochron, leaf angle). More phenotypic parameter extractions can be easily developed and incorporated to our pipeline as the biologists’ requirements evolve, allowing re-use of existing libraries of 3D models and the capacity to tailor the pipeline to new trait identification and quantification. Plant architecture is an important determinant of radiation use efficiency in crops and analysis of this trait in explicit 3D and over time has previously been an intractable problem with anything other than low throughput [1]. We should acknowledge, however, that tools for 3D analysis of roots based on inferred 3D “reconstructions” (i.e. 3D approximation using shapes such as tubes) exist and have been extensively used since the early 1960’s [20,21,40].

Although the methodology was solely tested on *Gossypium hirsutum* plants, it is expected that the method will be broadly and easily adaptable to other dicotyledonous crops such as canola, tomato, and low tillering monocotyledons with simple architectures such as corn. The pipeline can be easily adapted, and operators can be implemented and combined in order to increase the flexibility of the algorithm. Preliminary results (unpublished), obtained by reusing the two first steps of the segmentation pipeline (rough segmentation and stem segmentation) on corn, allowed to isolate the main stem, the leaves, and inter-nodes, and allowed the direct computation of corn specific data. Due to the importance of rice and wheat as major food crops, the application of image based plant phenomics tools to grasses is of great interest. Significant development of our pipeline is needed to cope with occlusions due to the complex structure and the tillering observed in cereal crops. Their investigation will involve pushing the state-of-the-art reconstruction and segmentation algorithms to their limit.

With respect to the accuracy on the phenotypic parameters, errors between 5 and 10% (involving ranges between 5mm to 7mm for the leaves) are acceptable for morphological scale phenotyping, reflecting the magnitude of errors already inherent in manual measurements and variations observed between individual plants of identical genetic make-up, and are low enough to distinguish changes in the relevant traits between two imaging dates during development (which is the aim of our research). Measurements for which the mean absolute error is above 10% (or over 10mm range, e.g. main stem measurements) will involve further work to improve the accuracy (for instance, the mean bias error - that characterises systematic over/under estimations - for the main stem height measurements was *MBE*_*s*_≃9.8*mm*, against *MBE*_*w*_≃−2.7*mm* and *MBE*_*l*_≃3.1*mm* for the leaf width and length measurements, entailing that a systematic over-estimation on the main stem height measurements is made). Finally, our current aim involves reducing the error on the measurements to less than 5%, which we believe is achievable by training our algorithms on phantom plant meshes (with phenotypic parameters exactly known) generated using existing plant modelling technologies [4,41].

While the focus of the current study has been the processing of meshes produced by a commercial 3D reconstruction product, major future work will involve improving the digitisation of plant structure and function by incorporating data other than visible light images into the 3D model. In addition to visible light cameras collecting multiple view geometries, *PlantScan*, a new screening platform recently developed in our laboratory [28] is equipped with LiDAR (Light Detection and Ranging sensors), infra-red cameras, and multi-wavelength cameras. The LiDAR cameras allow the reconstruction of accurate point-clouds (precision of 200 microns) that will be integrated in our probabilistic reconstruction scheme [29-31] in order to improve the accuracy of the reconstructed plant meshes that currently limits the quality of the morphological segmentation and temporal analysis. These meshes will be overlaid with thermal infra-red data and multi-spectral images data that provide colorimetric information (for chemical composition and photosynthetic functional analysis). Our laboratory expects to scan one plant every 7 minutes, making the current mesh-based methodology (3D reconstruction excluded) suitable for high-throughput dicotyledonous plant analysis. As 3DSOM required an average processing time of 15 minutes to reconstruct suitable meshes, a special focus will be placed on the efficiency of the reconstruction scheme developed.

## Conclusions

In this paper, we presented a hybrid mesh-based methodology developed for high-throughput plant phenomics research. The proposed solution provides advanced mesh-processing features, including plant mesh morphological segmentation, accurate plant aerial-part phenotypic parameters estimation, and individual organ tracking and data monitoring over-time. Experiments involved testing our processing pipeline on an initial set of six Gossypium hirsutum plants analysed over four time-points.

From the qualitative and quantitative results presented in the paper, we believe that the development of a mesh based methodology for high-resolution and high-throughput plant phenomics platform is feasible and offers multiple advantages over current systems that use a small number of 2D images. The hybrid mesh segmentation presented allowed the identification of the different plant organs for all the test plants. The phenotypic parameter estimation algorithms allowed the retrieval of measurements such as main stem height and inclination, petiole length and initiation angle, and leaf width, length, area and inclination. By comparing 384 mesh-based measurements with manual measurements, we observed errors ranging from 5.75% to 9.34% and correlations ranging from 0.887 to 0.974. The temporal organ tracking algorithm successfully matched plant organs between time-points in 95% of the cases. Finally, the proposed analysis required only 4.9 minutes in average to analyse a plant over four time-points. The mesh-based analysis is thus considered a suitable mean to perform accurate and efficient 3D plant phenotypic analysis.

## Competing interests

The authors declare that they have no competing interests.

## Authors’ contributions

XS, SB, and RF performed the manual data acquisition, 3D reconstruction and the manual measurements on the plants. AP and JF built the automated images and mesh based analysis pipeline (segmentation, data extraction, temporal analysis). AP, JF, and XS are the principal authors of this paper. All authors read and approved the final manuscript.

## Availability and requirements

The different operators presented in this paper, as well as an initial set of plant meshes, are available for download from the PlantScan home page (Microsoft Windows 64-bits installer): [28].

## Supplementary Material

Addtional file 1 **Website presenting the results.** Website containing the results obtained by applying our method on the initial set of plant meshes. The different results are presented as tables containing links to the different web-pages. The results of the segmentation and temporal matching between the different time-points are available as images. Phenotypic parameters estimated by our method are available in the form of tables. In addition, a spreadsheet containing all the mesh-based and manual measurements is available as a web-page and contains the statistical analysis presented in the paper. Click here for file
