# Supplementary material for: A novel mesh processing based technique for 3D plant analysis
Source: BMC Plant Biol. 2012 May 3;12:63. doi: 10.1186/1471-2229-12-63 (PMC3464618; doi:10.1186/1471-2229-12-63)
Supplement: Addtional file 1 — Website presenting the results. Website containing the results obtained by applying our method on the initial set of plant meshes. The different results are presented as tables containing links to the different web-pages. The results of the segmentation and temporal matching between the different time-points are available as images. Phenotypic parameters estimated by our method are available in the form of tables. In addition, a spreadsheet containing all the mesh-based and manual measurements is available as a web-page and contains the statistical analysis presented in the paper. [file 1471-2229-12-63-S1.zip › PlantPhenomics_mini_website_bmc/index.html]

# A novel mesh processing based technique for 3D plant analysisAuthors - Anthony Paproki - Jurgen Fripp - Xavier Sirault - Scott Berry - Robert Furbank Introduction This website comes as an addition to the following paper: "A novel mesh processing based technique for 3D plant analysis". You will find the different results mentioned in the paper under the form of images, tables, or spreadsheets. The segmentation and data extraction results are grouped per plant and time-point. The temporal analysis data are grouped per plant and involve analysing each plant over 4 time-points. The different graphs presented in the paper are available in the section spreadsheets, which contains html spreadsheets generated from Open-Office. All the results presented on this website were obtained on a computer equipped with a processor Intel Core 2 Duo E8300 (2.83GHz) and running under Ubuntu 10.04 LTS, 64 bits. Segmentation/data extraction results The following table contains the links to the pages presenting the results of the segmentation and data extraction algorithms. These data are separately available per plant and per date. Clicking on the links will forward you to a page containing the phenotypic parameters esrtimated and an illustration of the mesh segmentation. | Plant ID | Time-Point | Link | | --- | --- | --- | | Plant1 | T0 | Here | | Plant1 | T1 | Here | | Plant1 | T2 | Here | | Plant1 | T3 | Here | | Plant2 | T0 | Here | | Plant2 | T1 | Here | | Plant2 | T2 | Here | | Plant2 | T3 | Here | | Plant3 | T0 | Here | | Plant3 | T1 | Here | | Plant3 | T2 | Here | | Plant3 | T3 | Here | | Plant4 | T0 | Here | | Plant4 | T1 | Here | | Plant4 | T2 | Here | | Plant4 | T3 | Here | | Plant5 | T0 | Here | | Plant5 | T1 | Here | | Plant5 | T2 | Here | | Plant5 | T3 | Here | | Plant6 | T0 | Here | | Plant6 | T1 | Here | | Plant6 | T2 | Here | | Plant6 | T3 | Here | Temporal Matching / Analysis per plant The data presented in this section are grouped per plant. By clicking on the links, you'll be forwared to pages containing the temporal analysis of all the phenotypic parameters for a given plant. You will also find illustrations of the pair-wise matching. | Plant ID | Link | | --- | --- | | Plant1 | Here | | Plant2 | Here | | Plant3 | Here | | Plant4 | Here | | Plant5 | Here | | Plant6 | Here | Spreadsheets and graphs This page contains the statistical analysis made on the results we obtained. | Desctiption | Link | | --- | --- | | Statistical analysis | Here | Related Links The PlantScan project page The Australian e-Health Research Centre: contact Anthony Paproki The High-Resolution Plant Phenomics Centre: contact Xavier Sirault
