# Supplementary material for: A novel mesh processing based technique for 3D plant analysis
Source: BMC Plant Biol. 2012 May 3;12:63. doi: 10.1186/1471-2229-12-63 (PMC3464618; doi:10.1186/1471-2229-12-63)
Supplement: Addtional file 1 — Website presenting the results. Website containing the results obtained by applying our method on the initial set of plant meshes. The different results are presented as tables containing links to the different web-pages. The results of the segmentation and temporal matching between the different time-points are available as images. Phenotypic parameters estimated by our method are available in the form of tables. In addition, a spreadsheet containing all the mesh-based and manual measurements is available as a web-page and contains the statistical analysis presented in the paper. [file 1471-2229-12-63-S1.zip › PlantPhenomics_mini_website_bmc/spreadsheets/stats.html]

|  |  |  |  |  |  |  |  |  |  |  |  |  |  |  |  |  |  |  |  |  |  |  |  |  |  |  |  |  |  |  |  |  |  |  |  |  |  |  |  |  |  |  |
| --- | --- | --- | --- | --- | --- | --- | --- | --- | --- | --- | --- | --- | --- | --- | --- | --- | --- | --- | --- | --- | --- | --- | --- | --- | --- | --- | --- | --- | --- | --- | --- | --- | --- | --- | --- | --- | --- | --- | --- | --- | --- | --- |
| **Statistical analysis** |  |  |  |  |  |  |  |  |  |  |  |  |  |  |  |  |  |  |  |  |  |  |  |  |  |  |  |  |  |  |  |  |  |  |  |  |  |  |  |  |  |  |
|  |  |  |  |  |  |  |  |  |  |  |  |  |  |  |  |  |  |  |  |  |  |  |  |  |  |  |  |  |  |  |  |  |  |  |  |  |  |  |  |  |  |  |
| **Leaves Data** |  |  |  |  |  |  |  |  |  |  |  |  |  |  |  |  | **Values used to compute the ICC** |  |  |  |  |  |  | **Used for RMSE** |  | **Used for Bland/Altman** |  |  |  | **Used for MBE** |  | **Used for MAE** |  |  |  |  |  |  |  |  |  |  |
|  |  |  |  |  |  |  |  |  |  |  |  |  |  |  |  |  |  |  |  |  |  |  |  |  |  |  |  |  |  |  |  |  |  |  |  |  |  |  |  |  |  |  |
| **Plant ID** | **TP** | **Leaf ID** | **Automated Width** | **Automated Length** | **Automated Area** | **Manual Width (M)** | **Manual Length (M)** | **Width Error** | **Length Error** | **Width Range** | **Length Range** | **aw+mw** | **al + ml** | **(mw – w\_bar)^2** | **(ml – l\_bar)^2** | **(aw – w\_bar)^2** | **(al – l\_bar)^2** | **(mw - w\_bar)** | **(ml – l\_bar)** | **(aw – w\_bar)** | **(al – l\_bar)** | **W\*Y** | **X\*Z** | **(mw – aw)^2** | **(mw – aw)^2** | **log((mw+aw)/2)** | **log((ml+al)/2)** | **(mw-aw)/mw** | **(ml-al)/ml** | **(mw-aw)** | **(ml-al)** | **|mw-aw|** | **|ml-al|** |  | **Average Area** |  |  |  |  |  |  |  |
| Plant1 | T0 | 0 | 63.944 | 65.845 | 3490.905 | 61.000 | 61.000 | **0.048** | **0.079** | 2.944 | 4.845 | 124.944 | 126.845 | 707.414 | 378.102 | 559.476 | 213.156 | -26.597 | -19.445 | -23.653 | -14.600 | 629.111 | 283.892 | 8.667 | 23.474 | 1.796 | 1.802 | -0.048 | -0.079 | -2.944 | -4.845 | 2.944 | 4.845 |  |  |  |  |  |  |  |  |  |
| Plant1 | T0 | 1 | 91.292 | 93.466 | 5749.579 | 87.000 | 87.000 | **0.049** | **0.074** | 4.292 | 6.466 | 178.292 | 180.466 | 0.357 | 42.970 | 13.651 | 169.542 | -0.597 | 6.555 | 3.695 | 13.021 | -2.207 | 85.353 | 18.422 | 41.805 | 1.950 | 1.955 | -0.049 | -0.074 | -4.292 | -6.466 | 4.292 | 6.466 |  |  |  |  |  |  |  |  |  |
| Plant1 | T0 | 2 | 84.371 | 86.009 | 5341.932 | 79.000 | 88.000 | **0.068** | **0.023** | 5.371 | 1.991 | 163.371 | 174.009 | 73.913 | 57.080 | 10.409 | 30.958 | -8.597 | 7.555 | -3.226 | 5.564 | 27.738 | 42.036 | 28.847 | 3.965 | 1.912 | 1.940 | -0.068 | 0.023 | -5.371 | 1.991 | 5.371 | 1.991 |  |  |  |  |  |  |  |  |  |
| Plant1 | T0 | 3 | 41.958 | 32.673 | 1301.875 | 47.000 | 29.000 | **0.107** | **0.127** | 5.042 | 3.673 | 88.958 | 61.673 | 1648.137 | 2646.573 | 2082.986 | 2282.195 | -40.597 | -51.445 | -45.640 | -47.772 | 1852.848 | 2457.640 | 25.427 | 13.488 | 1.648 | 1.489 | 0.107 | -0.127 | 5.042 | -3.673 | 5.042 | 3.673 |  |  |  |  |  |  |  |  |  |
| Plant1 | T0 | 5 | 83.079 | 78.063 | 5075.179 | 82.000 | 80.000 | **0.013** | **0.024** | 1.079 | 1.937 | 165.079 | 158.063 | 31.329 | 0.198 | 20.411 | 5.676 | -5.597 | -0.445 | -4.518 | -2.382 | 25.287 | 1.060 | 1.165 | 3.754 | 1.917 | 1.898 | -0.013 | 0.024 | -1.079 | 1.937 | 1.079 | 1.937 |  |  |  |  |  |  |  |  |  |
| Plant1 | T0 | 6 | 59.271 | 38.968 | 2025.695 | 60.000 | 33.000 | **0.012** | **0.181** | 0.729 | 5.968 | 119.271 | 71.968 | 761.608 | 2251.014 | 802.375 | 1720.292 | -27.597 | -47.445 | -28.326 | -41.476 | 781.726 | 1967.842 | 0.531 | 35.622 | 1.776 | 1.556 | 0.012 | -0.181 | 0.729 | -5.968 | 0.729 | 5.968 |  | **3830.861** |  |  |  |  |  |  |  |
| Plant1 | T1 | 0 | 102.181 | 82 | 6284.439 | 104.000 | 107.000 | **0.017** | **0.236** | 1.819 | 25.210 | 206.181 | 188.790 | 269.050 | 705.176 | 212.686 | 1.810 | 16.403 | 26.555 | 14.584 | 1.345 | 239.214 | 35.727 | 3.309 | 635.532 | 2.013 | 1.975 | 0.017 | 0.236 | 1.819 | 25.210 | 1.819 | 25.210 |  |  |  |  |  |  |  |  |  |
| Plant1 | T1 | 1 | 117.796 | 109.424 | 9076.881 | 116.000 | 120.000 | **0.015** | **0.088** | 1.796 | 10.576 | 233.796 | 229.424 | 806.716 | 1564.609 | 911.972 | 839.799 | 28.403 | 39.555 | 30.199 | 28.979 | 857.731 | 1146.280 | 3.226 | 111.849 | 2.068 | 2.060 | -0.015 | 0.088 | -1.796 | 10.576 | 1.796 | 10.576 |  |  |  |  |  |  |  |  |  |
| Plant1 | T1 | 2 | 106.184 | 109.635 | 8400.000 | 107.000 | 115.000 | **0.008** | **0.047** | 0.816 | 5.365 | 213.184 | 224.635 | 376.467 | 1194.058 | 345.480 | 852.070 | 19.403 | 34.555 | 18.587 | 29.190 | 360.641 | 1008.673 | 0.665 | 28.782 | 2.028 | 2.050 | 0.008 | 0.047 | 0.816 | 5.365 | 0.816 | 5.365 |  |  |  |  |  |  |  |  |  |
| Plant1 | T1 | 3 | 76.522 | 72.640 | 4087.391 | 73.000 | 85.000 | **0.048** | **0.145** | 3.522 | 12.360 | 149.522 | 157.640 | 213.080 | 20.749 | 122.668 | 60.913 | -14.597 | 4.555 | -11.076 | -7.805 | 161.673 | -35.551 | 12.402 | 152.765 | 1.874 | 1.897 | -0.048 | 0.145 | -3.522 | 12.360 | 3.522 | 12.360 |  |  |  |  |  |  |  |  |  |
| Plant1 | T1 | 4 | 65.412 | 66.258 | 3369.319 | 64.000 | 72.000 | **0.022** | **0.080** | 1.412 | 5.742 | 129.412 | 138.258 | 556.830 | 71.316 | 492.164 | 201.267 | -23.597 | -8.445 | -22.185 | -14.187 | 523.499 | 119.806 | 1.995 | 32.971 | 1.811 | 1.840 | -0.022 | 0.080 | -1.412 | 5.742 | 1.412 | 5.742 |  |  |  |  |  |  |  |  |  |
| Plant1 | T1 | 5 | 43.767 | 34.196 | 1426.626 | 49.000 | 31.000 | **0.107** | **0.103** | 5.233 | 3.196 | 92.767 | 65.196 | 1489.748 | 2444.794 | 1921.067 | 2138.928 | -38.597 | -49.445 | -43.830 | -46.249 | 1691.717 | 2286.753 | 27.381 | 10.216 | 1.666 | 1.513 | 0.107 | -0.103 | 5.233 | -3.196 | 5.233 | 3.196 |  |  |  |  |  |  |  |  |  |
| Plant1 | T1 | 6 | 43.916 | 30.871 | 1103.607 | 64.000 | 34.000 | **0.314** | **0.092** | 20.084 | 3.129 | 107.916 | 64.871 | 556.830 | 2157.125 | 1908.086 | 2457.616 | -23.597 | -46.445 | -43.682 | -49.574 | 1030.767 | 2302.474 | 403.383 | 9.794 | 1.732 | 1.511 | 0.314 | 0.092 | 20.084 | 3.129 | 20.084 | 3.129 |  |  |  |  |  |  |  |  |  |
| Plant1 | T1 | 7 | 86.002 | 77.120 | 5158.269 | 87.000 | 85.000 | **0.011** | **0.093** | 0.998 | 7.880 | 173.002 | 162.120 | 0.357 | 20.749 | 2.546 | 11.052 | -0.597 | 4.555 | -1.596 | -3.324 | 0.953 | -15.143 | 0.997 | 62.088 | 1.937 | 1.909 | 0.011 | 0.093 | 0.998 | 7.880 | 0.998 | 7.880 |  | **4863.317** |  |  |  |  |  |  |  |
| Plant1 | T2 | 0 | 65.967 | 69 | 3648.262 | 64.000 | 67.000 | **0.031** | **0.028** | 1.967 | 1.853 | 129.967 | 135.853 | 556.830 | 180.764 | 467.884 | 134.375 | -23.597 | -13.445 | -21.631 | -11.592 | 510.423 | 155.853 | 3.868 | 3.433 | 1.813 | 1.832 | -0.031 | -0.028 | -1.967 | -1.853 | 1.967 | 1.853 |  |  |  |  |  |  |  |  |  |
| Plant1 | T2 | 1 | 125.630 | 116.937 | 11514.887 | 125.000 | 129.000 | **0.005** | **0.094** | 0.630 | 12.063 | 250.630 | 245.937 | 1398.965 | 2357.602 | 1446.477 | 1331.641 | 37.403 | 48.555 | 38.033 | 36.492 | 1422.523 | 1771.858 | 0.397 | 145.528 | 2.098 | 2.090 | -0.005 | 0.094 | -0.630 | 12.063 | 0.630 | 12.063 |  |  |  |  |  |  |  |  |  |
| Plant1 | T2 | 2 | 97.688 | 92.154 | 6108.760 | 96.000 | 95.000 | **0.018** | **0.030** | 1.688 | 2.846 | 193.688 | 187.154 | 70.606 | 211.852 | 101.822 | 137.111 | 8.403 | 14.555 | 10.091 | 11.709 | 84.789 | 170.432 | 2.849 | 8.098 | 1.986 | 1.971 | -0.018 | 0.030 | -1.688 | 2.846 | 1.688 | 2.846 |  |  |  |  |  |  |  |  |  |
| Plant1 | T2 | 3 | 88.813 | 86.885 | 5811.778 | 88.000 | 86.000 | **0.009** | **0.010** | 0.813 | 0.885 | 176.813 | 172.885 | 0.162 | 30.860 | 1.478 | 41.469 | 0.403 | 5.555 | 1.216 | 6.440 | 0.490 | 35.773 | 0.661 | 0.782 | 1.946 | 1.937 | -0.009 | -0.010 | -0.813 | -0.885 | 0.813 | 0.885 |  |  |  |  |  |  |  |  |  |
| Plant1 | T2 | 4 | 127.004 | 122.907 | 11208.716 | 122.000 | 130.000 | **0.041** | **0.055** | 5.004 | 7.093 | 249.004 | 252.907 | 1183.549 | 2455.712 | 1552.884 | 1803.066 | 34.403 | 49.555 | 39.407 | 42.463 | 1355.697 | 2104.237 | 25.039 | 50.305 | 2.095 | 2.102 | -0.041 | 0.055 | -5.004 | 7.093 | 5.004 | 7.093 |  |  |  |  |  |  |  |  |  |
| Plant1 | T2 | 5 | 83.382 | 72.664 | 3899.215 | 77.000 | 75.000 | **0.083** | **0.031** | 6.382 | 2.336 | 160.382 | 147.664 | 112.302 | 29.646 | 17.765 | 60.540 | -10.597 | -5.445 | -4.215 | -7.781 | 44.666 | 42.365 | 40.735 | 5.457 | 1.904 | 1.868 | -0.083 | 0.031 | -6.382 | 2.336 | 6.382 | 2.336 |  |  |  |  |  |  |  |  |  |
| Plant1 | T2 | 6 | 39.641 | 32.408 | 1152.519 | 49.000 | 31.000 | **0.191** | **0.045** | 9.359 | 1.408 | 88.641 | 63.408 | 1489.748 | 2444.794 | 2299.763 | 2307.574 | -38.597 | -49.445 | -47.956 | -48.037 | 1850.964 | 2375.193 | 87.583 | 1.981 | 1.647 | 1.501 | 0.191 | -0.045 | 9.359 | -1.408 | 9.359 | 1.408 |  |  |  |  |  |  |  |  |  |
| Plant1 | T2 | 7 | 134.715 | 121.038 | 12192.278 | 132.000 | 135.000 | **0.021** | **0.103** | 2.715 | 13.962 | 266.715 | 256.038 | 1971.604 | 2976.264 | 2220.108 | 1647.818 | 44.403 | 54.555 | 47.118 | 40.593 | 2092.169 | 2214.575 | 7.373 | 194.932 | 2.125 | 2.107 | -0.021 | 0.103 | -2.715 | 13.962 | 2.715 | 13.962 |  |  |  |  |  |  |  |  |  |
| Plant1 | T2 | 8 | 60.280 | 39.476 | 1967.479 | 64.000 | 34.000 | **0.058** | **0.161** | 3.720 | 5.476 | 124.280 | 73.476 | 556.830 | 2157.125 | 746.209 | 1678.460 | -23.597 | -46.445 | -27.317 | -40.969 | 644.602 | 1902.800 | 13.835 | 29.985 | 1.793 | 1.565 | 0.058 | -0.161 | 3.720 | -5.476 | 3.720 | 5.476 |  |  |  |  |  |  |  |  |  |
| Plant1 | T2 | 9 | 119.023 | 115.651 | 10376.039 | 115.000 | 115.000 | **0.035** | **0.006** | 4.023 | 0.651 | 234.023 | 230.651 | 750.911 | 1194.058 | 987.607 | 1239.442 | 27.403 | 34.555 | 31.426 | 35.206 | 861.165 | 1216.538 | 16.188 | 0.423 | 2.068 | 2.062 | -0.035 | -0.006 | -4.023 | -0.651 | 4.023 | 0.651 |  | **6787.993** |  |  |  |  |  |  |  |
| Plant1 | T3 | 0 | 135.150 | 125.891 | 12613.190 | 133.000 | 140.000 | **0.016** | **0.101** | 2.150 | 14.109 | 268.150 | 265.891 | 2061.409 | 3546.815 | 2261.279 | 2065.361 | 45.403 | 59.555 | 47.553 | 45.446 | 2159.032 | 2706.558 | 4.623 | 199.061 | 2.127 | 2.124 | -0.016 | 0.101 | -2.150 | 14.109 | 2.150 | 14.109 |  |  |  |  |  |  |  |  |  |
| Plant1 | T3 | 1 | 120.939 | 111.390 | 9747.177 | 112.000 | 118.000 | **0.080** | **0.056** | 8.939 | 6.610 | 232.939 | 229.390 | 595.494 | 1410.389 | 1111.700 | 957.601 | 24.403 | 37.555 | 33.342 | 30.945 | 813.640 | 1162.149 | 79.913 | 43.692 | 2.066 | 2.060 | -0.080 | 0.056 | -8.939 | 6.610 | 8.939 | 6.610 |  |  |  |  |  |  |  |  |  |
| Plant1 | T3 | 2 | 129.212 | 117.363 | 11209.553 | 127.000 | 130.000 | **0.017** | **0.097** | 2.212 | 12.637 | 256.212 | 247.363 | 1552.576 | 2455.712 | 1731.788 | 1362.980 | 39.403 | 49.555 | 41.615 | 36.919 | 1639.736 | 1829.504 | 4.893 | 159.683 | 2.108 | 2.092 | -0.017 | 0.097 | -2.212 | 12.637 | 2.212 | 12.637 |  |  |  |  |  |  |  |  |  |
| Plant1 | T3 | 3 | 88.620 | 85.344 | 5694.940 | 88.000 | 86.000 | **0.007** | **0.008** | 0.620 | 0.656 | 176.620 | 171.344 | 0.162 | 30.860 | 1.045 | 24.004 | 0.403 | 5.555 | 1.022 | 4.899 | 0.412 | 27.217 | 0.384 | 0.430 | 1.946 | 1.933 | -0.007 | 0.008 | -0.620 | 0.656 | 0.620 | 0.656 |  |  |  |  |  |  |  |  |  |
| Plant1 | T3 | 4 | 65.174 | 67.162 | 3354.250 | 64.000 | 70.000 | **0.018** | **0.041** | 1.174 | 2.838 | 129.174 | 137.162 | 556.830 | 109.095 | 502.810 | 176.437 | -23.597 | -10.445 | -22.423 | -13.283 | 529.131 | 138.738 | 1.378 | 8.055 | 1.810 | 1.836 | -0.018 | 0.041 | -1.174 | 2.838 | 1.174 | 2.838 |  |  |  |  |  |  |  |  |  |
| Plant1 | T3 | 5 | 123.684 | 107.477 | 9175.744 | 122.000 | 123.000 | **0.014** | **0.126** | 1.684 | 15.523 | 245.684 | 230.477 | 1183.549 | 1810.940 | 1302.239 | 730.715 | 34.403 | 42.555 | 36.087 | 27.032 | 1241.477 | 1150.340 | 2.835 | 240.976 | 2.089 | 2.062 | -0.014 | 0.126 | -1.684 | 15.523 | 1.684 | 15.523 |  |  |  |  |  |  |  |  |  |
| Plant1 | T3 | 6 | 61.672 | 35.609 | 1897.290 | 65.000 | 35.000 | **0.051** | **0.017** | 3.328 | 0.609 | 126.672 | 70.609 | 510.636 | 2065.235 | 672.127 | 2010.266 | -22.597 | -45.445 | -25.925 | -44.836 | 585.843 | 2037.565 | 11.077 | 0.371 | 1.802 | 1.548 | 0.051 | -0.017 | 3.328 | -0.609 | 3.328 | 0.609 |  |  |  |  |  |  |  |  |  |
| Plant1 | T3 | 9 | 121.271 | 99.169 | 7906.986 | 113.000 | 110.000 | **0.073** | **0.098** | 8.271 | 10.831 | 234.271 | 209.169 | 645.300 | 873.507 | 1133.934 | 350.601 | 25.403 | 29.555 | 33.674 | 18.724 | 855.411 | 553.400 | 68.413 | 117.306 | 2.069 | 2.019 | -0.073 | 0.098 | -8.271 | 10.831 | 8.271 | 10.831 |  |  |  |  |  |  |  |  |  |
| Plant1 | T3 | 10 | 45.156 | 27.825 | 1115.226 | 49.000 | 31.000 | **0.078** | **0.102** | 3.844 | 3.175 | 94.156 | 58.825 | 1489.748 | 2444.794 | 1801.228 | 2768.866 | -38.597 | -49.445 | -42.441 | -52.620 | 1638.101 | 2601.789 | 14.773 | 10.082 | 1.673 | 1.469 | 0.078 | 0.102 | 3.844 | 3.175 | 3.844 | 3.175 |  |  |  |  |  |  |  |  |  |
| Plant1 | T3 | 11 | 139.840 | 124.296 | 12052.339 | 137.000 | 141.000 | **0.021** | **0.118** | 2.840 | 16.704 | 276.840 | 265.296 | 2440.631 | 3666.925 | 2729.313 | 1922.903 | 49.403 | 60.555 | 52.243 | 43.851 | 2580.939 | 2655.399 | 8.066 | 279.031 | 2.141 | 2.123 | -0.021 | 0.118 | -2.840 | 16.704 | 2.840 | 16.704 |  | **7476.669** |  |  |  |  |  |  |  |
| Plant2 | T0 | 0 | 130.847 | 121.399 | 11784.927 | 103.000 | 111.000 | **0.270** | **0.094** | 27.847 | 10.399 | 233.847 | 232.399 | 237.245 | 933.617 | 1870.554 | 1677.278 | 15.403 | 30.555 | 43.250 | 40.955 | 666.167 | 1251.373 | 775.464 | 108.148 | 2.068 | 2.065 | -0.270 | -0.094 | -27.847 | -10.399 | 27.847 | 10.399 |  |  |  |  |  |  |  |  |  |
| Plant2 | T0 | 1 | 121.143 | 106.312 | 7510.527 | 85.000 | 87.000 | **0.425** | **0.222** | 36.143 | 19.312 | 206.143 | 193.312 | 6.746 | 42.970 | 1125.323 | 669.097 | -2.597 | 6.555 | 33.546 | 25.867 | -87.127 | 169.561 | 1306.323 | 372.944 | 2.013 | 1.985 | -0.425 | -0.222 | -36.143 | -19.312 | 36.143 | 19.312 |  |  |  |  |  |  |  |  |  |
| Plant2 | T0 | 2 | 91.061 | 84.685 | 6272.882 | 85.000 | 87.000 | **0.071** | **0.027** | 6.061 | 2.315 | 176.061 | 171.685 | 6.746 | 42.970 | 11.995 | 17.983 | -2.597 | 6.555 | 3.463 | 4.241 | -8.995 | 27.798 | 36.731 | 5.357 | 1.945 | 1.934 | -0.071 | 0.027 | -6.061 | 2.315 | 6.061 | 2.315 |  |  |  |  |  |  |  |  |  |
| Plant2 | T0 | 3 | 61.487 | 39.431 | 1941.654 | 65.000 | 37.000 | **0.054** | **0.066** | 3.513 | 2.431 | 126.487 | 76.431 | 510.636 | 1887.456 | 681.764 | 1682.145 | -22.597 | -43.445 | -26.111 | -41.014 | 590.028 | 1781.846 | 12.344 | 5.909 | 1.801 | 1.582 | 0.054 | -0.066 | 3.513 | -2.431 | 3.513 | 2.431 |  |  |  |  |  |  |  |  |  |
| Plant2 | T0 | 4 | 61.163 | 39.556 | 2257.039 | 59.000 | 33.000 | **0.037** | **0.199** | 2.163 | 6.556 | 120.163 | 72.556 | 817.803 | 2251.014 | 698.785 | 1671.896 | -28.597 | -47.445 | -26.435 | -40.889 | 755.955 | 1939.964 | 4.677 | 42.982 | 1.779 | 1.560 | -0.037 | -0.199 | -2.163 | -6.556 | 2.163 | 6.556 |  |  |  |  |  |  |  |  |  |
| Plant2 | T0 | 5 | 103.198 | 105.270 | 7730.621 | 97.000 | 95.000 | **0.064** | **0.108** | 6.198 | 10.270 | 200.198 | 200.270 | 88.412 | 211.852 | 243.395 | 616.287 | 9.403 | 14.555 | 15.601 | 24.825 | 146.693 | 361.333 | 38.420 | 105.472 | 2.000 | 2.001 | -0.064 | -0.108 | -6.198 | -10.270 | 6.198 | 10.270 |  | **6249.608** |  |  |  |  |  |  |  |
| Plant2 | T1 | 0 | 133.575 | 119.899 | 10475.776 | 96.000 | 107.000 | **0.391** | **0.121** | 37.575 | 12.899 | 229.575 | 226.899 | 70.606 | 705.176 | 2113.967 | 1556.636 | 8.403 | 26.555 | 45.978 | 39.454 | 386.341 | 1047.713 | 1411.892 | 166.386 | 2.060 | 2.055 | -0.391 | -0.121 | -37.575 | -12.899 | 37.575 | 12.899 |  |  |  |  |  |  |  |  |  |
| Plant2 | T1 | 1 | 140.878 | 140.753 | 13888.605 | 127.000 | 138.000 | **0.109** | **0.020** | 1288.067 | 7061.953 | 267.878 | 278.753 | 1552.576 | 3312.595 | 2838.824 | 3637.065 | 39.403 | 57.555 | 53.281 | 60.308 | 2099.403 | 3471.041 | 192.595 | 7.579 | 2.127 | 2.144 | -0.109 | -0.020 | -13.878 | -2.753 | 13.878 | 2.753 |  |  |  |  |  |  |  |  |  |
| Plant2 | T1 | 3 | 71.289 | 59.494 | 1645.351 | 54.000 | 61.000 | **0.320** | **0.025** | 185.816 | 2886.903 | 125.289 | 120.494 | 1128.775 | 378.102 | 265.946 | 438.953 | -33.597 | -19.445 | -16.308 | -20.951 | 547.899 | 407.393 | 298.924 | 2.269 | 1.797 | 1.780 | -0.320 | 0.025 | -17.289 | 1.506 | 17.289 | 1.506 |  |  |  |  |  |  |  |  |  |
| Plant2 | T1 | 7 | 107.199 | 103.833 | 8177.694 | 103.000 | 100.000 | **0.041** | **0.038** | 4.199 | 3.833 | 210.199 | 203.833 | 237.245 | 382.404 | 384.235 | 547.025 | 15.403 | 19.555 | 19.602 | 23.389 | 301.923 | 457.367 | 17.633 | 14.695 | 2.022 | 2.008 | -0.041 | -0.038 | -4.199 | -3.833 | 4.199 | 3.833 |  |  |  |  |  |  |  |  |  |
| Plant2 | T1 | 8 | 64.856 | 38.053 | 1706.024 | 66.000 | 37.000 | **0.017** | **0.028** | 1.144 | 1.053 | 130.856 | 75.053 | 466.441 | 1887.456 | 517.180 | 1797.086 | -21.597 | -43.445 | -22.742 | -42.392 | 491.156 | 1841.716 | 1.310 | 1.108 | 1.816 | 1.574 | 0.017 | -0.028 | 1.144 | -1.053 | 1.144 | 1.053 |  |  |  |  |  |  |  |  |  |
| Plant2 | T1 | 10 | 88.141 | 92.338 | 6039.754 | 85.000 | 89.000 | **0.037** | **0.038** | 3.141 | 3.338 | 173.141 | 181.338 | 6.746 | 73.190 | 0.296 | 141.442 | -2.597 | 8.555 | 0.544 | 11.893 | -1.413 | 101.746 | 9.869 | 11.141 | 1.937 | 1.957 | -0.037 | -0.038 | -3.141 | -3.338 | 3.141 | 3.338 |  |  |  |  |  |  |  |  |  |
| Plant2 | T1 | 11 | 148.789 | 130.922 | 13994.006 | 126.000 | 133.000 | **0.181** | **0.016** | 22.789 | 2.078 | 274.789 | 263.922 | 1474.771 | 2762.043 | 3744.406 | 2547.912 | 38.403 | 52.555 | 61.192 | 50.477 | 2349.924 | 2652.818 | 519.330 | 4.319 | 2.138 | 2.120 | -0.181 | 0.016 | -22.789 | 2.078 | 22.789 | 2.078 |  |  |  |  |  |  |  |  |  |
| Plant2 | T1 | 13 | 58.469 | 34.470 | 1754.817 | 64.000 | 34.000 | **0.086** | **0.014** | 5.531 | 0.470 | 122.469 | 68.470 | 556.830 | 2157.125 | 848.474 | 2113.694 | -23.597 | -46.445 | -29.129 | -45.975 | 687.354 | 2135.299 | 30.596 | 0.221 | 1.787 | 1.534 | 0.086 | -0.014 | 5.531 | -0.470 | 5.531 | 0.470 |  | **7210.253** |  |  |  |  |  |  |  |
| Plant2 | T2 | 0 | 135.808 | 120.231 | 10503.635 | 131.000 | 131.000 | **0.037** | **0.082** | 4.808 | 10.769 | 266.808 | 251.231 | 1883.798 | 2555.823 | 2324.309 | 1582.926 | 43.403 | 50.555 | 48.211 | 39.786 | 2092.494 | 2011.387 | 23.120 | 115.975 | 2.125 | 2.099 | -0.037 | 0.082 | -4.808 | 10.769 | 4.808 | 10.769 |  |  |  |  |  |  |  |  |  |
| Plant2 | T2 | 1 | 144.456 | 133.989 | 13229.168 | 150.000 | 155.000 | **0.037** | **0.136** | 5.544 | 21.011 | 294.456 | 288.989 | 3894.103 | 5558.469 | 3232.915 | 2866.976 | 62.403 | 74.555 | 56.859 | 53.544 | 3548.141 | 3991.992 | 30.736 | 441.462 | 2.168 | 2.160 | 0.037 | 0.136 | 5.544 | 21.011 | 5.544 | 21.011 |  |  |  |  |  |  |  |  |  |
| Plant2 | T2 | 2 | 75.681 | 61.557 | 1320.703 | 71.000 | 76.000 | **0.066** | **0.190** | 4.681 | 14.443 | 146.681 | 137.557 | 275.469 | 19.757 | 142.003 | 356.760 | -16.597 | -4.445 | -11.916 | -18.888 | 197.781 | 83.955 | 21.910 | 208.607 | 1.865 | 1.837 | -0.066 | 0.190 | -4.681 | 14.443 | 4.681 | 14.443 |  |  |  |  |  |  |  |  |  |
| Plant2 | T2 | 6 | 107.524 | 104.726 | 8343.037 | 106.000 | 106.000 | **0.014** | **0.012** | 1.524 | 1.274 | 213.524 | 210.726 | 338.661 | 653.065 | 397.064 | 589.586 | 18.403 | 25.555 | 19.926 | 24.281 | 366.701 | 620.514 | 2.322 | 1.622 | 2.028 | 2.023 | -0.014 | 0.012 | -1.524 | 1.274 | 1.524 | 1.274 |  |  |  |  |  |  |  |  |  |
| Plant2 | T2 | 8 | 86.683 | 88.985 | 6025.233 | 88.000 | 91.000 | **0.015** | **0.022** | 1.317 | 2.015 | 174.683 | 179.985 | 0.162 | 111.411 | 0.836 | 72.934 | 0.403 | 10.555 | -0.914 | 8.540 | -0.368 | 90.143 | 1.734 | 4.060 | 1.941 | 1.954 | 0.015 | 0.022 | 1.317 | 2.015 | 1.317 | 2.015 |  |  |  |  |  |  |  |  |  |
| Plant2 | T2 | 9 | 150.792 | 126.351 | 14007.193 | 145.000 | 150.000 | **0.040** | **0.158** | 5.792 | 23.649 | 295.792 | 276.351 | 3295.075 | 4837.918 | 3993.624 | 2107.355 | 57.403 | 69.555 | 63.195 | 45.906 | 3627.574 | 3192.994 | 33.552 | 559.285 | 2.170 | 2.140 | -0.040 | 0.158 | -5.792 | 23.649 | 5.792 | 23.649 |  |  |  |  |  |  |  |  |  |
| Plant2 | T2 | 11 | 58.814 | 35.839 | 1768.893 | 60.000 | 35.000 | **0.020** | **0.024** | 1.186 | 0.839 | 118.814 | 70.839 | 761.608 | 2065.235 | 828.486 | 1989.660 | -27.597 | -45.445 | -28.783 | -44.606 | 794.344 | 2027.095 | 1.407 | 0.704 | 1.774 | 1.549 | 0.020 | -0.024 | 1.186 | -0.839 | 1.186 | 0.839 |  |  |  |  |  |  |  |  |  |
| Plant2 | T2 | 12 | 65.113 | 48.123 | 2296.158 | 66.000 | 38.000 | **0.013** | **0.266** | 0.887 | 10.123 | 131.113 | 86.123 | 466.441 | 1801.566 | 505.557 | 1044.683 | -21.597 | -42.445 | -22.485 | -32.322 | 485.606 | 1371.884 | 0.787 | 102.481 | 1.817 | 1.634 | 0.013 | -0.266 | 0.887 | -10.123 | 0.887 | 10.123 |  |  |  |  |  |  |  |  |  |
| Plant2 | T2 | 13 | 139.889 | 133.047 | 13617.899 | 132.000 | 145.000 | **0.060** | **0.082** | 7.889 | 11.953 | 271.889 | 278.047 | 1971.604 | 4167.367 | 2734.377 | 2766.947 | 44.403 | 64.555 | 52.291 | 52.602 | 2321.876 | 3395.715 | 62.229 | 142.883 | 2.133 | 2.143 | -0.060 | 0.082 | -7.889 | 11.953 | 7.889 | 11.953 |  | **7901.324** |  |  |  |  |  |  |  |
| Plant2 | T3 | 0 | 151.323 | 147.307 | 15371.719 | 150.000 | 155.000 | **0.009** | **0.050** | 1.323 | 7.693 | 301.323 | 302.307 | 3894.103 | 5558.469 | 4060.981 | 4470.490 | 62.403 | 74.555 | 63.726 | 66.862 | 3976.667 | 4984.885 | 1.751 | 59.189 | 2.178 | 2.179 | -0.009 | 0.050 | -1.323 | 7.693 | 1.323 | 7.693 |  |  |  |  |  |  |  |  |  |
| Plant2 | T3 | 1 | 145.245 | 143.096 | 15058.503 | 139.000 | 150.000 | **0.045** | **0.046** | 6.245 | 6.904 | 284.245 | 293.096 | 2642.242 | 4837.918 | 3323.226 | 3925.121 | 51.403 | 69.555 | 57.647 | 62.651 | 2963.236 | 4357.684 | 38.996 | 47.670 | 2.153 | 2.166 | -0.045 | 0.046 | -6.245 | 6.904 | 6.245 | 6.904 |  |  |  |  |  |  |  |  |  |
| Plant2 | T3 | 2 | 156.516 | 112.840 | 12120.205 | 149.000 | 150.000 | **0.050** | **0.248** | 7.516 | 37.160 | 305.516 | 262.840 | 3770.297 | 4837.918 | 4749.801 | 1049.458 | 61.403 | 69.555 | 68.919 | 32.395 | 4231.804 | 2253.262 | 56.491 | 1380.851 | 2.184 | 2.119 | -0.050 | 0.248 | -7.516 | 37.160 | 7.516 | 37.160 |  |  |  |  |  |  |  |  |  |
| Plant2 | T3 | 3 | 61.891 | 41.549 | 678.901 | 60.000 | 65.000 | **0.032** | **0.361** | 1.891 | 23.451 | 121.891 | 106.549 | 761.608 | 238.544 | 660.790 | 1512.853 | -27.597 | -15.445 | -25.706 | -38.895 | 709.410 | 600.734 | 3.577 | 549.928 | 1.785 | 1.727 | -0.032 | 0.361 | -1.891 | 23.451 | 1.891 | 23.451 |  |  |  |  |  |  |  |  |  |
| Plant2 | T3 | 4 | 160.716 | 148.529 | 16848.045 | 165.000 | 161.000 | **0.026** | **0.077** | 4.284 | 12.471 | 325.716 | 309.529 | 5991.185 | 6489.131 | 5346.360 | 4635.485 | 77.403 | 80.555 | 73.119 | 68.084 | 5659.597 | 5484.548 | 18.352 | 155.520 | 2.212 | 2.190 | 0.026 | 0.077 | 4.284 | 12.471 | 4.284 | 12.471 |  |  |  |  |  |  |  |  |  |
| Plant2 | T3 | 6 | 103.264 | 104.497 | 8144.787 | 108.000 | 108.000 | **0.044** | **0.032** | 4.736 | 3.503 | 211.264 | 212.497 | 416.272 | 759.286 | 245.456 | 578.519 | 20.403 | 27.555 | 15.667 | 24.052 | 319.651 | 662.768 | 22.427 | 12.269 | 2.024 | 2.026 | 0.044 | 0.032 | 4.736 | 3.503 | 4.736 | 3.503 |  |  |  |  |  |  |  |  |  |
| Plant2 | T3 | 8 | 51.848 | 41.503 | 1907.593 | 66.000 | 39.000 | **0.214** | **0.064** | 14.152 | 2.503 | 117.848 | 80.503 | 466.441 | 1717.676 | 1278.037 | 1516.490 | -21.597 | -41.445 | -35.750 | -38.942 | 772.094 | 1613.951 | 200.290 | 6.264 | 1.770 | 1.605 | 0.214 | -0.064 | 14.152 | -2.503 | 14.152 | 2.503 |  |  |  |  |  |  |  |  |  |
| Plant2 | T3 | 10 | 108.039 | 97.817 | 7274.255 | 102.000 | 111.000 | **0.059** | **0.119** | 6.039 | 13.183 | 210.039 | 208.817 | 207.439 | 933.617 | 417.855 | 301.780 | 14.403 | 30.555 | 20.442 | 17.372 | 294.414 | 530.798 | 36.467 | 173.800 | 2.021 | 2.019 | -0.059 | 0.119 | -6.039 | 13.183 | 6.039 | 13.183 |  |  |  |  |  |  |  |  |  |
| Plant2 | T3 | 12 | 55.212 | 37.348 | 1963.992 | 61.000 | 35.000 | **0.095** | **0.067** | 5.788 | 2.348 | 116.212 | 72.348 | 707.414 | 2065.235 | 1048.806 | 1857.372 | -26.597 | -45.445 | -32.385 | -43.097 | 861.360 | 1958.548 | 33.501 | 5.511 | 1.764 | 1.558 | 0.095 | -0.067 | 5.788 | -2.348 | 5.788 | 2.348 |  | **8818.667** |  |  |  |  |  |  |  |
| Plant3 | T0 | 0 | 67.231 | 59.142 | 3166.567 | 67.000 | 68.000 | **0.003** | **0.130** | 0.231 | 8.858 | 134.231 | 127.142 | 424.247 | 154.874 | 414.795 | 453.820 | -20.597 | -12.445 | -20.367 | -21.303 | 419.495 | 265.113 | 0.053 | 78.468 | 1.827 | 1.803 | -0.003 | 0.130 | -0.231 | 8.858 | 0.231 | 8.858 |  |  |  |  |  |  |  |  |  |
| Plant3 | T0 | 1 | 80.519 | 62.632 | 3454.592 | 69.000 | 71.000 | **0.167** | **0.118** | 11.519 | 8.368 | 149.519 | 133.632 | 345.858 | 89.205 | 50.107 | 317.285 | -18.597 | -9.445 | -7.079 | -17.813 | 131.643 | 168.237 | 132.678 | 70.017 | 1.874 | 1.825 | -0.167 | 0.118 | -11.519 | 8.368 | 11.519 | 8.368 |  |  |  |  |  |  |  |  |  |
| Plant3 | T0 | 2 | 76.299 | 91.767 | 4503.378 | 79.000 | 76.000 | **0.034** | **0.207** | 2.701 | 15.767 | 155.299 | 167.767 | 73.913 | 19.757 | 127.652 | 128.197 | -8.597 | -4.445 | -11.298 | 11.322 | 97.134 | -50.327 | 7.296 | 248.607 | 1.890 | 1.924 | 0.034 | -0.207 | 2.701 | -15.767 | 2.701 | 15.767 |  |  |  |  |  |  |  |  |  |
| Plant3 | T0 | 3 | 53.906 | 30.835 | 1662.204 | 59.000 | 31.000 | **0.086** | **0.005** | 5.094 | 0.165 | 112.906 | 61.835 | 817.803 | 2444.794 | 1135.116 | 2461.121 | -28.597 | -49.445 | -33.691 | -49.610 | 963.484 | 2452.944 | 25.951 | 0.027 | 1.752 | 1.490 | 0.086 | 0.005 | 5.094 | 0.165 | 5.094 | 0.165 |  |  |  |  |  |  |  |  |  |
| Plant3 | T0 | 4 | 56.612 | 41.070 | 2156.531 | 62.000 | 35.000 | **0.087** | **0.173** | 5.388 | 6.070 | 118.612 | 76.070 | 655.219 | 2065.235 | 960.102 | 1550.386 | -25.597 | -45.445 | -30.986 | -39.375 | 793.144 | 1789.389 | 29.033 | 36.844 | 1.773 | 1.580 | 0.087 | -0.173 | 5.388 | -6.070 | 5.388 | 6.070 |  |  |  |  |  |  |  |  |  |
| Plant3 | T0 | 5 | 47.472 | 40.363 | 1346.203 | 45.000 | 42.000 | **0.055** | **0.039** | 2.472 | 1.637 | 92.472 | 82.363 | 1814.526 | 1478.007 | 1610.012 | 1606.585 | -42.597 | -38.445 | -40.125 | -40.082 | 1709.213 | 1540.955 | 6.112 | 2.681 | 1.665 | 1.615 | -0.055 | 0.039 | -2.472 | 1.637 | 2.472 | 1.637 |  | **2714.912** |  |  |  |  |  |  |  |
| Plant3 | T1 | 0 | 76.461 | 78.012 | 4646.594 | 75.000 | 75.000 | **0.019** | **0.040** | 1.461 | 3.012 | 151.461 | 153.012 | 158.691 | 29.646 | 124.009 | 5.920 | -12.597 | -5.445 | -11.136 | -2.433 | 140.282 | 13.248 | 2.135 | 9.070 | 1.879 | 1.884 | -0.019 | -0.040 | -1.461 | -3.012 | 1.461 | 3.012 |  |  |  |  |  |  |  |  |  |
| Plant3 | T1 | 1 | 105.881 | 94.298 | 7269.008 | 98.000 | 97.000 | **0.080** | **0.028** | 7.881 | 2.702 | 203.881 | 191.298 | 108.217 | 274.073 | 334.300 | 191.910 | 10.403 | 16.555 | 18.284 | 13.853 | 190.203 | 229.341 | 62.112 | 7.301 | 2.008 | 1.981 | -0.080 | 0.028 | -7.881 | 2.702 | 7.881 | 2.702 |  |  |  |  |  |  |  |  |  |
| Plant3 | T1 | 2 | 67.518 | 65.812 | 3328.047 | 63.000 | 65.000 | **0.072** | **0.012** | 4.518 | 0.812 | 130.518 | 130.812 | 605.025 | 238.544 | 403.172 | 214.120 | -24.597 | -15.445 | -20.079 | -14.633 | 493.892 | 226.002 | 20.413 | 0.659 | 1.815 | 1.816 | -0.072 | -0.012 | -4.518 | -0.812 | 4.518 | 0.812 |  |  |  |  |  |  |  |  |  |
| Plant3 | T1 | 3 | 60.389 | 41.808 | 2234.001 | 64.000 | 36.000 | **0.056** | **0.161** | 3.611 | 5.808 | 124.389 | 77.808 | 556.830 | 1975.345 | 740.287 | 1492.785 | -23.597 | -44.445 | -27.208 | -38.637 | 642.039 | 1717.197 | 13.039 | 33.736 | 1.794 | 1.590 | 0.056 | -0.161 | 3.611 | -5.808 | 3.611 | 5.808 |  |  |  |  |  |  |  |  |  |
| Plant3 | T1 | 4 | 106.859 | 106.834 | 8378.072 | 106.000 | 108.000 | **0.008** | **0.011** | 0.859 | 1.166 | 212.859 | 214.834 | 338.661 | 759.286 | 371.019 | 696.373 | 18.403 | 27.555 | 19.262 | 26.389 | 354.471 | 727.150 | 0.738 | 1.360 | 2.027 | 2.031 | -0.008 | 0.011 | -0.859 | 1.166 | 0.859 | 1.166 |  |  |  |  |  |  |  |  |  |
| Plant3 | T1 | 5 | 61.630 | 59.209 | 1660.119 | 60.000 | 67.000 | **0.027** | **0.116** | 1.630 | 7.791 | 121.630 | 126.209 | 761.608 | 180.764 | 674.279 | 450.953 | -27.597 | -13.445 | -25.967 | -21.236 | 716.615 | 285.510 | 2.658 | 60.697 | 1.784 | 1.800 | -0.027 | 0.116 | -1.630 | 7.791 | 1.630 | 7.791 |  |  |  |  |  |  |  |  |  |
| Plant3 | T1 | 6 | 58.228 | 34.031 | 1742.902 | 61.000 | 31.000 | **0.045** | **0.098** | 2.772 | 3.031 | 119.228 | 65.031 | 707.414 | 2444.794 | 862.540 | 2154.230 | -26.597 | -49.445 | -29.369 | -46.414 | 781.135 | 2294.918 | 7.683 | 9.188 | 1.775 | 1.512 | 0.045 | -0.098 | 2.772 | -3.031 | 2.772 | 3.031 |  | **4179.821** |  |  |  |  |  |  |  |
| Plant3 | T2 | 0 | 129.219 | 118.693 | 10443.787 | 123.000 | 123.000 | **0.051** | **0.035** | 6.219 | 4.307 | 252.219 | 241.693 | 1253.355 | 1810.940 | 1732.343 | 1462.887 | 35.403 | 42.555 | 41.621 | 38.248 | 1473.513 | 1627.636 | 38.672 | 18.554 | 2.101 | 2.082 | -0.051 | 0.035 | -6.219 | 4.307 | 6.219 | 4.307 |  |  |  |  |  |  |  |  |  |
| Plant3 | T2 | 1 | 98.151 | 77.813 | 4260.392 | 91.000 | 92.000 | **0.079** | **0.154** | 7.151 | 14.187 | 189.151 | 169.813 | 11.579 | 133.521 | 111.392 | 6.927 | 3.403 | 11.555 | 10.554 | -2.632 | 35.913 | -30.412 | 51.144 | 201.273 | 1.976 | 1.929 | -0.079 | 0.154 | -7.151 | 14.187 | 7.151 | 14.187 |  |  |  |  |  |  |  |  |  |
| Plant3 | T2 | 2 | 123.323 | 112.472 | 8108.631 | 117.000 | 124.000 | **0.054** | **0.093** | 6.323 | 11.528 | 240.323 | 236.472 | 864.522 | 1897.051 | 1276.339 | 1025.747 | 29.403 | 43.555 | 35.726 | 32.027 | 1050.439 | 1394.953 | 39.982 | 132.892 | 2.080 | 2.073 | -0.054 | 0.093 | -6.323 | 11.528 | 6.323 | 11.528 |  |  |  |  |  |  |  |  |  |
| Plant3 | T2 | 3 | 62.863 | 37.568 | 2295.280 | 65.000 | 37.000 | **0.033** | **0.015** | 2.137 | 0.568 | 127.863 | 74.568 | 510.636 | 1887.456 | 611.765 | 1838.424 | -22.597 | -43.445 | -24.734 | -42.877 | 558.918 | 1862.779 | 4.565 | 0.323 | 1.806 | 1.572 | 0.033 | -0.015 | 2.137 | -0.568 | 2.137 | 0.568 |  |  |  |  |  |  |  |  |  |
| Plant3 | T2 | 4 | 55.426 | 32.151 | 1768.992 | 62.000 | 34.000 | **0.106** | **0.054** | 6.574 | 1.849 | 117.426 | 66.151 | 655.219 | 2157.125 | 1034.996 | 2332.262 | -25.597 | -46.445 | -32.171 | -48.293 | 823.498 | 2242.985 | 43.219 | 3.417 | 1.769 | 1.520 | 0.106 | 0.054 | 6.574 | 1.849 | 6.574 | 1.849 |  |  |  |  |  |  |  |  |  |
| Plant3 | T2 | 5 | 65.754 | 67.980 | 3487.591 | 68.000 | 69.000 | **0.033** | **0.015** | 2.246 | 1.020 | 133.754 | 136.980 | 384.052 | 130.985 | 477.131 | 155.374 | -19.597 | -11.445 | -21.843 | -12.465 | 428.069 | 142.659 | 5.045 | 1.041 | 1.825 | 1.836 | 0.033 | 0.015 | 2.246 | 1.020 | 2.246 | 1.020 |  |  |  |  |  |  |  |  |  |
| Plant3 | T2 | 6 | 117.001 | 111.967 | 9707.519 | 115.000 | 122.000 | **0.017** | **0.082** | 2.001 | 10.033 | 232.001 | 233.967 | 750.911 | 1726.830 | 864.561 | 993.673 | 27.403 | 41.555 | 29.403 | 31.523 | 805.734 | 1309.926 | 4.003 | 100.652 | 2.064 | 2.068 | -0.017 | 0.082 | -2.001 | 10.033 | 2.001 | 10.033 |  |  |  |  |  |  |  |  |  |
| Plant3 | T2 | 7 | 77.437 | 77.882 | 5134.285 | 80.000 | 83.000 | **0.032** | **0.062** | 2.563 | 5.118 | 157.437 | 160.882 | 57.718 | 6.529 | 103.237 | 6.566 | -7.597 | 2.555 | -10.161 | -2.562 | 77.192 | -6.547 | 6.571 | 26.189 | 1.896 | 1.905 | 0.032 | 0.062 | 2.563 | 5.118 | 2.563 | 5.118 |  | **5650.810** |  |  |  |  |  |  |  |
| Plant3 | T3 | 0 | 137.292 | 124.238 | 11549.599 | 130.000 | 130.000 | **0.056** | **0.044** | 7.292 | 5.762 | 267.292 | 254.238 | 1797.993 | 2455.712 | 2469.547 | 1917.846 | 42.403 | 49.555 | 49.695 | 43.793 | 2107.185 | 2170.179 | 53.170 | 33.200 | 2.126 | 2.104 | -0.056 | 0.044 | -7.292 | 5.762 | 7.292 | 5.762 |  |  |  |  |  |  |  |  |  |
| Plant3 | T3 | 1 | 123.307 | 116.321 | 8878.941 | 119.000 | 120.000 | **0.036** | **0.031** | 4.307 | 3.679 | 242.307 | 236.321 | 986.133 | 1564.609 | 1275.186 | 1287.097 | 31.403 | 39.555 | 35.710 | 35.876 | 1121.384 | 1419.086 | 18.550 | 13.535 | 2.083 | 2.072 | -0.036 | 0.031 | -4.307 | 3.679 | 4.307 | 3.679 |  |  |  |  |  |  |  |  |  |
| Plant3 | T3 | 2 | 67.962 | 62.327 | 3422.764 | 68.000 | 67.000 | **0.001** | **0.070** | 0.038 | 4.673 | 135.962 | 129.327 | 384.052 | 180.764 | 385.558 | 328.250 | -19.597 | -13.445 | -19.636 | -18.118 | 384.804 | 243.589 | 0.001 | 21.835 | 1.832 | 1.811 | 0.001 | 0.070 | 0.038 | 4.673 | 0.038 | 4.673 |  |  |  |  |  |  |  |  |  |
| Plant3 | T3 | 4 | 79.146 | 65.596 | 5104.108 | 83.000 | 87.000 | **0.046** | **0.246** | 3.854 | 21.404 | 162.146 | 152.596 | 21.135 | 42.970 | 71.420 | 220.480 | -4.597 | 6.555 | -8.451 | -14.849 | 38.852 | -97.335 | 14.852 | 458.119 | 1.909 | 1.883 | 0.046 | 0.246 | 3.854 | 21.404 | 3.854 | 21.404 |  |  |  |  |  |  |  |  |  |
| Plant3 | T3 | 5 | 62.263 | 61.594 | 1967.814 | 59.000 | 64.000 | **0.055** | **0.038** | 3.263 | 2.406 | 121.263 | 125.594 | 817.803 | 270.433 | 641.832 | 355.359 | -28.597 | -16.445 | -25.334 | -18.851 | 724.494 | 310.001 | 10.646 | 5.789 | 1.783 | 1.798 | -0.055 | 0.038 | -3.263 | 2.406 | 3.263 | 2.406 |  |  |  |  |  |  |  |  |  |
| Plant3 | T3 | 6 | 137.759 | 137.479 | 12192.730 | 131.000 | 136.000 | **0.052** | **0.011** | 6.759 | 1.479 | 268.759 | 273.479 | 1883.798 | 3086.374 | 2516.169 | 3252.880 | 43.403 | 55.555 | 50.161 | 57.034 | 2177.144 | 3168.533 | 45.680 | 2.187 | 2.128 | 2.136 | -0.052 | -0.011 | -6.759 | -1.479 | 6.759 | 1.479 |  |  |  |  |  |  |  |  |  |
| Plant3 | T3 | 8 | 62.250 | 41.948 | 2354.340 | 65.000 | 37.000 | **0.042** | **0.134** | 2.750 | 4.948 | 127.250 | 78.948 | 510.636 | 1887.456 | 642.479 | 1481.998 | -22.597 | -43.445 | -25.347 | -38.497 | 572.776 | 1672.485 | 7.562 | 24.484 | 1.804 | 1.596 | 0.042 | -0.134 | 2.750 | -4.948 | 2.750 | 4.948 |  |  |  |  |  |  |  |  |  |
| Plant3 | T3 | 9 | 56.398 | 31.682 | 1886.879 | 62.000 | 32.000 | **0.090** | **0.010** | 5.602 | 0.318 | 118.398 | 63.682 | 655.219 | 2346.904 | 973.396 | 2377.827 | -25.597 | -48.445 | -31.199 | -48.763 | 798.616 | 2362.315 | 31.383 | 0.101 | 1.772 | 1.503 | 0.090 | 0.010 | 5.602 | 0.318 | 5.602 | 0.318 |  |  |  |  |  |  |  |  |  |
| Plant3 | T3 | 10 | 122.899 | 120.746 | 9990.872 | 117.000 | 124.000 | **0.050** | **0.026** | 5.899 | 3.254 | 239.899 | 244.746 | 864.522 | 1897.051 | 1246.197 | 1624.207 | 29.403 | 43.555 | 35.302 | 40.301 | 1037.961 | 1755.336 | 34.795 | 10.586 | 2.079 | 2.088 | -0.050 | 0.026 | -5.899 | 3.254 | 5.899 | 3.254 |  | **6372.005** |  |  |  |  |  |  |  |
| Plant4 | T0 | 0 | 62.053 | 38.343 | 2021.243 | 60.000 | 33.000 | **0.034** | **0.162** | 2.053 | 5.343 | 122.053 | 71.343 | 761.608 | 2251.014 | 652.528 | 1772.579 | -27.597 | -47.445 | -25.545 | -42.102 | 704.961 | 1997.524 | 4.213 | 28.546 | 1.786 | 1.552 | -0.034 | -0.162 | -2.053 | -5.343 | 2.053 | 5.343 |  |  |  |  |  |  |  |  |  |
| Plant4 | T0 | 1 | 44.226 | 49.796 | 1065.692 | 43.000 | 50.000 | **0.029** | **0.004** | 1.226 | 0.204 | 87.226 | 99.796 | 1988.915 | 926.889 | 1881.082 | 939.341 | -44.597 | -30.445 | -43.371 | -30.649 | 1934.247 | 933.095 | 1.503 | 0.042 | 1.640 | 1.698 | -0.029 | 0.004 | -1.226 | 0.204 | 1.226 | 0.204 |  |  |  |  |  |  |  |  |  |
| Plant4 | T0 | 2 | 83.306 | 81.048 | 4843.218 | 81.000 | 81.000 | **0.028** | **0.001** | 2.306 | 0.048 | 164.306 | 162.048 | 43.524 | 0.308 | 18.417 | 0.364 | -6.597 | 0.555 | -4.291 | 0.603 | 28.312 | 0.335 | 5.317 | 0.002 | 1.915 | 1.909 | -0.028 | -0.001 | -2.306 | -0.048 | 2.306 | 0.048 |  |  |  |  |  |  |  |  |  |
| Plant4 | T0 | 3 | 58.142 | 35.334 | 1744.315 | 58.000 | 31.000 | **0.002** | **0.140** | 0.142 | 4.334 | 116.142 | 66.334 | 875.997 | 2444.794 | 867.618 | 2034.989 | -29.597 | -49.445 | -29.455 | -45.111 | 871.797 | 2230.499 | 0.020 | 18.784 | 1.764 | 1.521 | -0.002 | -0.140 | -0.142 | -4.334 | 0.142 | 4.334 |  |  |  |  |  |  |  |  |  |
| Plant4 | T0 | 4 | 71.182 | 84.093 | 4151.811 | 71.000 | 78.000 | **0.003** | **0.078** | 0.182 | 6.093 | 142.182 | 162.093 | 275.469 | 5.977 | 269.452 | 13.310 | -16.597 | -2.445 | -16.415 | 3.648 | 272.444 | -8.919 | 0.033 | 37.126 | 1.852 | 1.909 | -0.003 | -0.078 | -0.182 | -6.093 | 0.182 | 6.093 |  | **2765.256** |  |  |  |  |  |  |  |
| Plant4 | T1 | 0 | 91.668 | 73.088 | 4312.955 | 77.000 | 88.000 | **0.190** | **0.169** | 14.668 | 14.912 | 168.668 | 161.088 | 112.302 | 57.080 | 16.568 | 54.122 | -10.597 | 7.555 | 4.070 | -7.357 | -43.135 | -55.581 | 215.141 | 222.365 | 1.926 | 1.906 | -0.190 | 0.169 | -14.668 | 14.912 | 14.668 | 14.912 |  |  |  |  |  |  |  |  |  |
| Plant4 | T1 | 2 | 63.391 | 36.220 | 2106.814 | 62.000 | 34.000 | **0.022** | **0.065** | 1.391 | 2.220 | 125.391 | 70.220 | 655.219 | 2157.125 | 585.959 | 1955.834 | -25.597 | -46.445 | -24.207 | -44.225 | 619.622 | 2054.015 | 1.934 | 4.929 | 1.797 | 1.545 | -0.022 | -0.065 | -1.391 | -2.220 | 1.391 | 2.220 |  |  |  |  |  |  |  |  |  |
| Plant4 | T1 | 3 | 76.576 | 82.503 | 4755.063 | 74.000 | 82.000 | **0.035** | **0.006** | 2.576 | 0.503 | 150.576 | 164.503 | 184.885 | 2.418 | 121.474 | 4.234 | -13.597 | 1.555 | -11.022 | 2.058 | 149.863 | 3.200 | 6.634 | 0.253 | 1.877 | 1.915 | -0.035 | -0.006 | -2.576 | -0.503 | 2.576 | 0.503 |  |  |  |  |  |  |  |  |  |
| Plant4 | T1 | 4 | 99.167 | 103.032 | 7255.367 | 93.000 | 109.000 | **0.066** | **0.055** | 6.167 | 5.968 | 192.167 | 212.032 | 29.190 | 815.396 | 133.862 | 510.183 | 5.403 | 28.555 | 11.570 | 22.587 | 62.509 | 644.981 | 38.033 | 35.616 | 1.983 | 2.025 | -0.066 | 0.055 | -6.167 | 5.968 | 6.167 | 5.968 |  |  |  |  |  |  |  |  |  |
| Plant4 | T1 | 5 | 95.990 | 91.930 | 6579.486 | 92.000 | 93.000 | **0.043** | **0.012** | 3.990 | 1.070 | 187.990 | 184.930 | 19.384 | 157.632 | 70.440 | 131.906 | 4.403 | 12.555 | 8.393 | 11.485 | 36.952 | 144.196 | 15.921 | 1.145 | 1.973 | 1.966 | -0.043 | 0.012 | -3.990 | 1.070 | 3.990 | 1.070 |  |  |  |  |  |  |  |  |  |
| Plant4 | T1 | 6 | 53.145 | 37.983 | 1816.005 | 60.000 | 31.000 | **0.114** | **0.225** | 6.855 | 6.983 | 113.145 | 68.983 | 761.608 | 2444.794 | 1186.987 | 1803.010 | -27.597 | -49.445 | -34.453 | -42.462 | 950.799 | 2099.521 | 46.997 | 48.762 | 1.753 | 1.538 | 0.114 | -0.225 | 6.855 | -6.983 | 6.855 | 6.983 |  | **4470.948** |  |  |  |  |  |  |  |
| Plant4 | T2 | 0 | 102.381 | 94.154 | 6083.619 | 98.000 | 105.000 | **0.045** | **0.103** | 4.381 | 10.846 | 200.381 | 199.154 | 108.217 | 602.955 | 218.564 | 187.950 | 10.403 | 24.555 | 14.784 | 13.709 | 153.793 | 336.638 | 19.195 | 117.629 | 2.001 | 1.998 | -0.045 | 0.103 | -4.381 | 10.846 | 4.381 | 10.846 |  |  |  |  |  |  |  |  |  |
| Plant4 | T2 | 1 | 117.448 | 125.735 | 10497.540 | 117.000 | 137.000 | **0.004** | **0.082** | 0.448 | 11.265 | 234.448 | 262.735 | 864.522 | 3198.484 | 891.086 | 2051.204 | 29.403 | 56.555 | 29.851 | 45.290 | 877.703 | 2561.395 | 0.201 | 126.898 | 2.069 | 2.118 | -0.004 | 0.082 | -0.448 | 11.265 | 0.448 | 11.265 |  |  |  |  |  |  |  |  |  |
| Plant4 | T2 | 2 | 81.730 | 89.885 | 5346.166 | 78.000 | 88.000 | **0.048** | **0.021** | 3.730 | 1.885 | 159.730 | 177.885 | 92.107 | 57.080 | 34.422 | 89.123 | -9.597 | 7.555 | -5.867 | 9.440 | 56.308 | 71.324 | 13.914 | 3.554 | 1.902 | 1.949 | -0.048 | -0.021 | -3.730 | -1.885 | 3.730 | 1.885 |  |  |  |  |  |  |  |  |  |
| Plant4 | T2 | 3 | 62.948 | 37.935 | 2071.586 | 64.000 | 34.000 | **0.016** | **0.116** | 1.052 | 3.935 | 126.948 | 71.935 | 556.830 | 2157.125 | 607.570 | 1807.102 | -23.597 | -46.445 | -24.649 | -42.510 | 581.647 | 1974.372 | 1.106 | 15.483 | 1.803 | 1.556 | 0.016 | -0.116 | 1.052 | -3.935 | 1.052 | 3.935 |  |  |  |  |  |  |  |  |  |
| Plant4 | T2 | 4 | 57.921 | 35.750 | 1890.770 | 61.000 | 33.000 | **0.050** | **0.083** | 3.079 | 2.750 | 118.921 | 68.750 | 707.414 | 2251.014 | 880.701 | 1997.606 | -26.597 | -47.445 | -29.677 | -44.695 | 789.316 | 2120.528 | 9.482 | 7.564 | 1.774 | 1.536 | 0.050 | -0.083 | 3.079 | -2.750 | 3.079 | 2.750 |  |  |  |  |  |  |  |  |  |
| Plant4 | T2 | 5 | 130.456 | 117.132 | 10932.912 | 125.000 | 128.000 | **0.044** | **0.085** | 5.456 | 10.868 | 255.456 | 245.132 | 1398.965 | 2261.492 | 1836.851 | 1345.982 | 37.403 | 47.555 | 42.859 | 36.688 | 1603.026 | 1744.685 | 29.765 | 118.103 | 2.106 | 2.088 | -0.044 | 0.085 | -5.456 | 10.868 | 5.456 | 10.868 |  |  |  |  |  |  |  |  |  |
| Plant4 | T2 | 6 | 102.095 | 98.598 | 7566.164 | 100.000 | 100.000 | **0.021** | **0.014** | 2.095 | 1.402 | 202.095 | 198.598 | 153.828 | 382.404 | 210.180 | 329.539 | 12.403 | 19.555 | 14.498 | 18.153 | 179.810 | 354.989 | 4.388 | 1.965 | 2.005 | 1.997 | -0.021 | 0.014 | -2.095 | 1.402 | 2.095 | 1.402 |  | **6341.251** |  |  |  |  |  |  |  |
| Plant4 | T3 | 0 | 94.559 | 86.459 | 4553.126 | 87.000 | 92.000 | **0.087** | **0.060** | 7.559 | 5.541 | 181.559 | 178.459 | 0.357 | 133.521 | 48.468 | 36.171 | -0.597 | 11.555 | 6.962 | 6.014 | -4.158 | 69.495 | 57.141 | 30.702 | 1.958 | 1.951 | -0.087 | 0.060 | -7.559 | 5.541 | 7.559 | 5.541 |  |  |  |  |  |  |  |  |  |
| Plant4 | T3 | 1 | 63.474 | 38.653 | 2242.010 | 64.000 | 34.000 | **0.008** | **0.137** | 0.526 | 4.653 | 127.474 | 72.653 | 556.830 | 2157.125 | 581.936 | 1746.552 | -23.597 | -46.445 | -24.123 | -41.792 | 569.245 | 1941.013 | 0.277 | 21.651 | 1.804 | 1.560 | 0.008 | -0.137 | 0.526 | -4.653 | 0.526 | 4.653 |  |  |  |  |  |  |  |  |  |
| Plant4 | T3 | 2 | 116.643 | 103.202 | 9413.100 | 123.000 | 131.000 | **0.052** | **0.212** | 6.357 | 27.798 | 239.643 | 234.202 | 1253.355 | 2555.823 | 843.662 | 517.902 | 35.403 | 50.555 | 29.046 | 22.757 | 1028.303 | 1150.507 | 40.410 | 772.711 | 2.079 | 2.069 | 0.052 | 0.212 | 6.357 | 27.798 | 6.357 | 27.798 |  |  |  |  |  |  |  |  |  |
| Plant4 | T3 | 4 | 131.508 | 139.260 | 12401.433 | 123.000 | 143.000 | **0.069** | **0.026** | 8.508 | 3.740 | 254.508 | 282.260 | 1253.355 | 3913.146 | 1928.148 | 3459.273 | 35.403 | 62.555 | 43.911 | 58.816 | 1554.559 | 3679.218 | 72.385 | 13.984 | 2.105 | 2.150 | -0.069 | 0.026 | -8.508 | 3.740 | 8.508 | 3.740 |  |  |  |  |  |  |  |  |  |
| Plant4 | T3 | 5 | 59.415 | 37.207 | 1824.034 | 61.000 | 33.000 | **0.026** | **0.127** | 1.585 | 4.207 | 120.415 | 70.207 | 707.414 | 2251.014 | 794.231 | 1869.533 | -26.597 | -47.445 | -28.182 | -43.238 | 749.566 | 2051.425 | 2.512 | 17.697 | 1.780 | 1.545 | 0.026 | -0.127 | 1.585 | -4.207 | 1.585 | 4.207 |  |  |  |  |  |  |  |  |  |
| Plant4 | T3 | 6 | 84.685 | 87.294 | 5691.749 | 80.000 | 89.000 | **0.059** | **0.019** | 4.685 | 1.706 | 164.685 | 176.294 | 57.718 | 73.190 | 8.482 | 46.905 | -7.597 | 8.555 | -2.912 | 6.849 | 22.127 | 58.592 | 21.948 | 2.912 | 1.916 | 1.945 | -0.059 | 0.019 | -4.685 | 1.706 | 4.685 | 1.706 |  |  |  |  |  |  |  |  |  |
| Plant4 | T3 | 7 | 144.247 | 131.530 | 12965.828 | 137.000 | 136.000 | **0.053** | **0.033** | 7.247 | 4.470 | 281.247 | 267.530 | 2440.631 | 3086.374 | 3209.141 | 2609.649 | 49.403 | 55.555 | 56.649 | 51.085 | 2798.630 | 2838.019 | 52.512 | 19.985 | 2.148 | 2.126 | -0.053 | 0.033 | -7.247 | 4.470 | 7.247 | 4.470 |  |  |  |  |  |  |  |  |  |
| Plant4 | T3 | 8 | 108.176 | 109.251 | 8156.318 | 102.000 | 101.000 | **0.061** | **0.082** | 6.176 | 8.251 | 210.176 | 210.251 | 207.439 | 422.514 | 423.482 | 829.797 | 14.403 | 20.555 | 20.579 | 28.806 | 296.390 | 592.116 | 38.142 | 68.080 | 2.022 | 2.022 | -0.061 | -0.082 | -6.176 | -8.251 | 6.176 | 8.251 |  | **7155.950** |  |  |  |  |  |  |  |
| Plant5 | T0 | 0 | 59.194 | 33.310 | 1734.652 | 58.000 | 30.000 | **0.021** | **0.110** | 1.194 | 3.310 | 117.194 | 63.310 | 875.997 | 2544.684 | 806.745 | 2221.739 | -29.597 | -50.445 | -28.403 | -47.135 | 840.658 | 2377.735 | 1.426 | 10.953 | 1.768 | 1.500 | -0.021 | -0.110 | -1.194 | -3.310 | 1.194 | 3.310 |  |  |  |  |  |  |  |  |  |
| Plant5 | T0 | 1 | 56.499 | 34.143 | 1632.971 | 54.000 | 29.000 | **0.046** | **0.177** | 2.499 | 5.143 | 110.499 | 63.143 | 1128.775 | 2646.573 | 967.101 | 2143.869 | -33.597 | -51.445 | -31.098 | -46.302 | 1044.815 | 2381.996 | 6.245 | 26.450 | 1.742 | 1.499 | -0.046 | -0.177 | -2.499 | -5.143 | 2.499 | 5.143 |  |  |  |  |  |  |  |  |  |
| Plant5 | T0 | 2 | 75.901 | 75.631 | 3964.375 | 72.000 | 73.000 | **0.054** | **0.036** | 3.901 | 2.631 | 147.901 | 148.631 | 243.274 | 55.426 | 136.796 | 23.170 | -15.597 | -7.445 | -11.696 | -4.814 | 182.425 | 35.836 | 15.220 | 6.924 | 1.869 | 1.871 | -0.054 | -0.036 | -3.901 | -2.631 | 3.901 | 2.631 |  |  |  |  |  |  |  |  |  |
| Plant5 | T0 | 3 | 64.688 | 65.302 | 3425.976 | 62.000 | 64.000 | **0.043** | **0.020** | 2.688 | 1.302 | 126.688 | 129.302 | 655.219 | 270.433 | 524.850 | 229.312 | -25.597 | -16.445 | -22.910 | -15.143 | 586.423 | 249.025 | 7.223 | 1.695 | 1.802 | 1.811 | -0.043 | -0.020 | -2.688 | -1.302 | 2.688 | 1.302 |  |  |  |  |  |  |  |  |  |
| Plant5 | T0 | 4 | 40.798 | 38.744 | 690.413 | 36.000 | 38.000 | **0.133** | **0.020** | 4.798 | 0.744 | 76.798 | 76.744 | 2662.277 | 1801.566 | 2190.191 | 1738.946 | -51.597 | -42.445 | -46.799 | -41.701 | 2414.724 | 1769.979 | 23.019 | 0.554 | 1.584 | 1.584 | -0.133 | -0.020 | -4.798 | -0.744 | 4.798 | 0.744 |  | **2289.677** |  |  |  |  |  |  |  |
| Plant5 | T1 | 0 | 87.731 | 85.540 | 5464.697 | 85.000 | 85.000 | **0.032** | **0.006** | 2.731 | 0.540 | 172.731 | 170.540 | 6.746 | 20.749 | 0.018 | 25.961 | -2.597 | 4.555 | 0.134 | 5.095 | -0.349 | 23.209 | 7.461 | 0.292 | 1.936 | 1.931 | -0.032 | -0.006 | -2.731 | -0.540 | 2.731 | 0.540 |  |  |  |  |  |  |  |  |  |
| Plant5 | T1 | 1 | 78.728 | 73.738 | 3676.014 | 75.000 | 80.000 | **0.050** | **0.078** | 3.728 | 6.262 | 153.728 | 153.738 | 158.691 | 0.198 | 78.660 | 44.976 | -12.597 | -0.445 | -8.869 | -6.706 | 111.726 | 2.983 | 13.900 | 39.207 | 1.886 | 1.886 | -0.050 | 0.078 | -3.728 | 6.262 | 3.728 | 6.262 |  |  |  |  |  |  |  |  |  |
| Plant5 | T1 | 2 | 91.384 | 85.985 | 5777.120 | 84.000 | 91.000 | **0.088** | **0.055** | 7.384 | 5.015 | 175.384 | 176.985 | 12.940 | 111.411 | 14.336 | 30.696 | -3.597 | 10.555 | 3.786 | 5.540 | -13.620 | 58.480 | 54.517 | 25.148 | 1.943 | 1.947 | -0.088 | 0.055 | -7.384 | 5.015 | 7.384 | 5.015 |  |  |  |  |  |  |  |  |  |
| Plant5 | T1 | 3 | 68.037 | 70.889 | 3682.713 | 65.000 | 70.000 | **0.047** | **0.013** | 3.037 | 0.889 | 133.037 | 140.889 | 510.636 | 109.095 | 382.591 | 91.307 | -22.597 | -10.445 | -19.560 | -9.555 | 442.001 | 99.806 | 9.225 | 0.791 | 1.823 | 1.848 | -0.047 | -0.013 | -3.037 | -0.889 | 3.037 | 0.889 |  |  |  |  |  |  |  |  |  |
| Plant5 | T1 | 4 | 57.615 | 33.361 | 1585.222 | 57.000 | 30.000 | **0.011** | **0.112** | 0.615 | 3.361 | 114.615 | 63.361 | 936.192 | 2544.684 | 898.958 | 2216.935 | -30.597 | -50.445 | -29.983 | -47.084 | 917.386 | 2375.163 | 0.378 | 11.293 | 1.758 | 1.501 | -0.011 | -0.112 | -0.615 | -3.361 | 0.615 | 3.361 |  |  |  |  |  |  |  |  |  |
| Plant5 | T1 | 5 | 59.331 | 34.329 | 1724.701 | 58.000 | 31.000 | **0.023** | **0.107** | 1.331 | 3.329 | 117.331 | 65.329 | 875.997 | 2444.794 | 799.002 | 2126.654 | -29.597 | -49.445 | -28.267 | -46.116 | 836.614 | 2280.182 | 1.771 | 11.084 | 1.768 | 1.514 | -0.023 | -0.107 | -1.331 | -3.329 | 1.331 | 3.329 |  | **3651.745** |  |  |  |  |  |  |  |
| Plant5 | T2 | 0 | 58.971 | 32.756 | 1764.775 | 61.000 | 31.000 | **0.033** | **0.057** | 2.029 | 1.756 | 119.971 | 63.756 | 707.414 | 2444.794 | 819.434 | 2274.209 | -26.597 | -49.445 | -28.626 | -47.689 | 761.367 | 2357.959 | 4.115 | 3.084 | 1.778 | 1.503 | 0.033 | -0.057 | 2.029 | -1.756 | 2.029 | 1.756 |  |  |  |  |  |  |  |  |  |
| Plant5 | T2 | 1 | 70.125 | 66.308 | 3905.630 | 66.000 | 71.000 | **0.063** | **0.066** | 4.125 | 4.692 | 136.125 | 137.308 | 466.441 | 89.205 | 305.267 | 199.849 | -21.597 | -9.445 | -17.472 | -14.137 | 377.345 | 133.520 | 17.019 | 22.014 | 1.833 | 1.837 | -0.063 | 0.066 | -4.125 | 4.692 | 4.125 | 4.692 |  |  |  |  |  |  |  |  |  |
| Plant5 | T2 | 2 | 118.568 | 99.135 | 8598.905 | 115.000 | 120.000 | **0.031** | **0.174** | 3.568 | 20.865 | 233.568 | 219.135 | 750.911 | 1564.609 | 959.160 | 349.314 | 27.403 | 39.555 | 30.970 | 18.690 | 848.671 | 739.284 | 12.727 | 435.356 | 2.067 | 2.040 | -0.031 | 0.174 | -3.568 | 20.865 | 3.568 | 20.865 |  |  |  |  |  |  |  |  |  |
| Plant5 | T2 | 3 | 99.678 | 89.428 | 5777.334 | 91.000 | 97.000 | **0.095** | **0.078** | 8.678 | 7.572 | 190.678 | 186.428 | 11.579 | 274.073 | 145.936 | 80.690 | 3.403 | 16.555 | 12.080 | 8.983 | 41.107 | 148.710 | 75.301 | 57.341 | 1.979 | 1.969 | -0.095 | 0.078 | -8.678 | 7.572 | 8.678 | 7.572 |  |  |  |  |  |  |  |  |  |
| Plant5 | T2 | 4 | 56.640 | 31.282 | 1581.912 | 58.000 | 31.000 | **0.023** | **0.009** | 1.360 | 0.282 | 114.640 | 62.282 | 875.997 | 2444.794 | 958.373 | 2416.939 | -29.597 | -49.445 | -30.958 | -49.162 | 916.260 | 2430.827 | 1.851 | 0.080 | 1.758 | 1.493 | 0.023 | -0.009 | 1.360 | -0.282 | 1.360 | 0.282 |  |  |  |  |  |  |  |  |  |
| Plant5 | T2 | 5 | 114.338 | 104.511 | 8629.779 | 109.000 | 117.000 | **0.049** | **0.107** | 5.338 | 12.489 | 223.338 | 221.511 | 458.078 | 1336.279 | 715.053 | 579.173 | 21.403 | 36.555 | 26.740 | 24.066 | 572.320 | 879.736 | 28.491 | 155.979 | 2.048 | 2.044 | -0.049 | 0.107 | -5.338 | 12.489 | 5.338 | 12.489 |  |  |  |  |  |  |  |  |  |
| Plant5 | T2 | 6 | 47.619 | 43.584 | 1114.378 | 48.000 | 50.000 | **0.008** | **0.128** | 0.381 | 6.416 | 95.619 | 93.584 | 1567.942 | 926.889 | 1598.286 | 1358.710 | -39.597 | -30.445 | -39.979 | -36.861 | 1583.041 | 1122.218 | 0.145 | 41.163 | 1.680 | 1.670 | 0.008 | 0.128 | 0.381 | 6.416 | 0.381 | 6.416 |  |  |  |  |  |  |  |  |  |
| Plant5 | T2 | 7 | 93.563 | 89.033 | 5886.774 | 91.000 | 92.000 | **0.028** | **0.032** | 2.563 | 2.967 | 184.563 | 181.033 | 11.579 | 133.521 | 35.591 | 73.749 | 3.403 | 11.555 | 5.966 | 8.588 | 20.300 | 99.233 | 6.569 | 8.805 | 1.965 | 1.957 | -0.028 | 0.032 | -2.563 | 2.967 | 2.563 | 2.967 |  | **4657.436** |  |  |  |  |  |  |  |
| Plant5 | T3 | 0 | 121.603 | 100.565 | 8185.162 | 120.000 | 126.000 | **0.013** | **0.202** | 1.603 | 25.435 | 241.603 | 226.565 | 1049.938 | 2075.271 | 1156.395 | 404.801 | 32.403 | 45.555 | 34.006 | 20.120 | 1101.882 | 916.555 | 2.570 | 646.963 | 2.082 | 2.054 | -0.013 | 0.202 | -1.603 | 25.435 | 1.603 | 25.435 |  |  |  |  |  |  |  |  |  |
| Plant5 | T3 | 1 | 120.597 | 110.731 | 10215.958 | 116.000 | 122.000 | **0.040** | **0.092** | 4.597 | 11.269 | 236.597 | 232.731 | 806.716 | 1726.830 | 1088.968 | 917.269 | 28.403 | 41.555 | 33.000 | 30.286 | 937.277 | 1258.558 | 21.130 | 126.984 | 2.073 | 2.066 | -0.040 | 0.092 | -4.597 | 11.269 | 4.597 | 11.269 |  |  |  |  |  |  |  |  |  |
| Plant5 | T3 | 2 | 89.020 | 81.981 | 4629.501 | 84.000 | 87.000 | **0.060** | **0.058** | 5.020 | 5.019 | 173.020 | 168.981 | 12.940 | 42.970 | 2.025 | 2.361 | -3.597 | 6.555 | 1.423 | 1.536 | -5.119 | 10.072 | 25.204 | 25.187 | 1.937 | 1.927 | -0.060 | 0.058 | -5.020 | 5.019 | 5.020 | 5.019 |  |  |  |  |  |  |  |  |  |
| Plant5 | T3 | 3 | 71.770 | 71.743 | 4232.002 | 68.000 | 71.000 | **0.055** | **0.010** | 3.770 | 0.743 | 139.770 | 142.743 | 384.052 | 89.205 | 250.512 | 75.726 | -19.597 | -9.445 | -15.828 | -8.702 | 310.177 | 82.190 | 14.210 | 0.552 | 1.844 | 1.854 | -0.055 | -0.010 | -3.770 | -0.743 | 3.770 | 0.743 |  |  |  |  |  |  |  |  |  |
| Plant5 | T3 | 4 | 63.347 | 53.399 | 2323.582 | 60.000 | 31.000 | **0.056** | **0.723** | 3.347 | 22.399 | 123.347 | 84.399 | 761.608 | 2444.794 | 588.060 | 731.500 | -27.597 | -49.445 | -24.250 | -27.046 | 669.232 | 1337.298 | 11.204 | 501.697 | 1.790 | 1.625 | -0.056 | -0.723 | -3.347 | -22.399 | 3.347 | 22.399 |  |  |  |  |  |  |  |  |  |
| Plant5 | T3 | 5 | 57.778 | 35.242 | 1742.311 | 58.000 | 31.000 | **0.004** | **0.137** | 0.222 | 4.242 | 115.778 | 66.242 | 875.997 | 2444.794 | 889.180 | 2043.271 | -29.597 | -49.445 | -29.819 | -45.203 | 882.564 | 2235.034 | 0.049 | 17.997 | 1.763 | 1.520 | 0.004 | -0.137 | 0.222 | -4.242 | 0.222 | 4.242 |  |  |  |  |  |  |  |  |  |
| Plant5 | T3 | 7 | 133.306 | 120.052 | 11209.630 | 128.000 | 132.000 | **0.041** | **0.091** | 5.306 | 11.948 | 261.306 | 252.052 | 1632.382 | 2657.933 | 2089.307 | 1568.700 | 40.403 | 51.555 | 45.709 | 39.607 | 1846.767 | 2041.935 | 28.156 | 142.763 | 2.116 | 2.100 | -0.041 | 0.091 | -5.306 | 11.948 | 5.306 | 11.948 |  |  |  |  |  |  |  |  |  |
| Plant5 | T3 | 8 | 93.771 | 93.991 | 7310.183 | 93.000 | 93.000 | **0.008** | **0.011** | 0.771 | 0.991 | 186.771 | 186.991 | 29.190 | 157.632 | 38.115 | 183.496 | 5.403 | 12.555 | 6.174 | 13.546 | 33.355 | 170.073 | 0.594 | 0.982 | 1.970 | 1.971 | -0.008 | -0.011 | -0.771 | -0.991 | 0.771 | 0.991 |  | **6231.041** |  |  |  |  |  |  |  |
| Plant6 | T0 | 0 | 84.484 | 77.921 | 4491.451 | 78.000 | 80.000 | **0.083** | **0.026** | 6.484 | 2.079 | 162.484 | 157.921 | 92.107 | 0.198 | 9.694 | 6.372 | -9.597 | -0.445 | -3.114 | -2.524 | 29.881 | 1.123 | 42.039 | 4.324 | 1.910 | 1.897 | -0.083 | 0.026 | -6.484 | 2.079 | 6.484 | 2.079 |  |  |  |  |  |  |  |  |  |
| Plant6 | T0 | 1 | 58.752 | 58.123 | 3038.654 | 59.000 | 62.000 | **0.004** | **0.063** | 0.248 | 3.877 | 117.752 | 120.123 | 817.803 | 340.213 | 832.051 | 498.256 | -28.597 | -18.445 | -28.845 | -22.322 | 824.896 | 411.720 | 0.062 | 15.029 | 1.770 | 1.779 | 0.004 | 0.063 | 0.248 | 3.877 | 0.248 | 3.877 |  |  |  |  |  |  |  |  |  |
| Plant6 | T0 | 2 | 57.489 | 37.235 | 1844.639 | 58.000 | 32.000 | **0.009** | **0.164** | 0.511 | 5.235 | 115.489 | 69.235 | 875.997 | 2346.904 | 906.536 | 1867.109 | -29.597 | -48.445 | -30.109 | -43.210 | 891.136 | 2093.305 | 0.262 | 27.403 | 1.762 | 1.539 | 0.009 | -0.164 | 0.511 | -5.235 | 0.511 | 5.235 |  |  |  |  |  |  |  |  |  |
| Plant6 | T0 | 3 | 55.761 | 44.054 | 1058.280 | 52.000 | 53.000 | **0.072** | **0.169** | 3.761 | 8.946 | 107.761 | 97.054 | 1267.164 | 753.220 | 1013.561 | 1324.331 | -35.597 | -27.445 | -31.836 | -36.391 | 1133.291 | 998.755 | 14.143 | 80.040 | 1.731 | 1.686 | -0.072 | 0.169 | -3.761 | 8.946 | 3.761 | 8.946 |  |  |  |  |  |  |  |  |  |
| Plant6 | T0 | 4 | 88.091 | 83.985 | 5344.377 | 85.000 | 83.000 | **0.036** | **0.012** | 3.091 | 0.985 | 173.091 | 166.985 | 6.746 | 6.529 | 0.244 | 12.533 | -2.597 | 2.555 | 0.494 | 3.540 | -1.283 | 9.046 | 9.556 | 0.970 | 1.937 | 1.922 | -0.036 | -0.012 | -3.091 | -0.985 | 3.091 | 0.985 |  |  |  |  |  |  |  |  |  |
| Plant6 | T0 | 5 | 53.612 | 32.970 | 1535.720 | 54.000 | 30.000 | **0.007** | **0.099** | 0.388 | 2.970 | 107.612 | 62.970 | 1128.775 | 2544.684 | 1155.012 | 2253.815 | -33.597 | -50.445 | -33.985 | -47.474 | 1141.818 | 2394.837 | 0.151 | 8.824 | 1.731 | 1.498 | 0.007 | -0.099 | 0.388 | -2.970 | 0.388 | 2.970 |  | **2885.520** |  |  |  |  |  |  |  |
| Plant6 | T1 | 0 | 109.405 | 104.823 | 7474.050 | 94.000 | 104.000 | **0.164** | **0.008** | 15.405 | 0.823 | 203.405 | 208.823 | 40.995 | 554.845 | 475.559 | 594.283 | 6.403 | 23.555 | 21.807 | 24.378 | 139.627 | 574.225 | 237.300 | 0.677 | 2.007 | 2.019 | -0.164 | -0.008 | -15.405 | -0.823 | 15.405 | 0.823 |  |  |  |  |  |  |  |  |  |
| Plant6 | T1 | 1 | 94.639 | 85.459 | 5867.055 | 90.000 | 89.000 | **0.052** | **0.040** | 4.639 | 3.541 | 184.639 | 174.459 | 5.773 | 73.190 | 49.591 | 25.139 | 2.403 | 8.555 | 7.042 | 5.014 | 16.920 | 42.894 | 21.523 | 12.541 | 1.965 | 1.941 | -0.052 | 0.040 | -4.639 | 3.541 | 4.639 | 3.541 |  |  |  |  |  |  |  |  |  |
| Plant6 | T1 | 2 | 77.398 | 70.846 | 3086.959 | 71.000 | 80.000 | **0.090** | **0.114** | 6.398 | 9.154 | 148.398 | 150.846 | 275.469 | 0.198 | 104.018 | 92.132 | -16.597 | -0.445 | -10.199 | -9.599 | 169.274 | 4.270 | 40.939 | 83.790 | 1.870 | 1.878 | -0.090 | 0.114 | -6.398 | 9.154 | 6.398 | 9.154 |  |  |  |  |  |  |  |  |  |
| Plant6 | T1 | 3 | 51.693 | 31.882 | 1515.387 | 53.000 | 31.000 | **0.025** | **0.028** | 1.307 | 0.882 | 104.693 | 62.882 | 1196.970 | 2444.794 | 1289.111 | 2358.303 | -34.597 | -49.445 | -35.904 | -48.562 | 1242.186 | 2401.159 | 1.708 | 0.779 | 1.719 | 1.497 | 0.025 | -0.028 | 1.307 | -0.882 | 1.307 | 0.882 |  |  |  |  |  |  |  |  |  |
| Plant6 | T1 | 4 | 56.151 | 35.687 | 1817.723 | 56.000 | 32.000 | **0.003** | **0.115** | 0.151 | 3.687 | 112.151 | 67.687 | 998.386 | 2346.904 | 988.883 | 2003.293 | -31.597 | -48.445 | -31.447 | -44.758 | 993.624 | 2168.303 | 0.023 | 13.592 | 1.749 | 1.529 | -0.003 | -0.115 | -0.151 | -3.687 | 0.151 | 3.687 |  |  |  |  |  |  |  |  |  |
| Plant6 | T1 | 5 | 62.491 | 67.574 | 3248.401 | 57.000 | 77.000 | **0.096** | **0.122** | 5.491 | 9.426 | 119.491 | 144.574 | 936.192 | 11.867 | 630.322 | 165.647 | -30.597 | -3.445 | -25.106 | -12.870 | 768.181 | 44.337 | 30.151 | 88.840 | 1.776 | 1.859 | -0.096 | 0.122 | -5.491 | 9.426 | 5.491 | 9.426 |  |  |  |  |  |  |  |  |  |
| Plant6 | T1 | 6 | 118.238 | 109.654 | 8762.600 | 110.000 | 111.000 | **0.075** | **0.012** | 8.238 | 1.346 | 228.238 | 220.654 | 501.883 | 933.617 | 938.879 | 853.149 | 22.403 | 30.555 | 30.641 | 29.209 | 686.445 | 892.477 | 67.871 | 1.813 | 2.057 | 2.043 | -0.075 | 0.012 | -8.238 | 1.346 | 8.238 | 1.346 |  | **4538.882** |  |  |  |  |  |  |  |
| Plant6 | T2 | 0 | 134.362 | 119.617 | 11644.696 | 125.000 | 128.000 | **0.075** | **0.065** | 9.362 | 8.383 | 259.362 | 247.617 | 1398.965 | 2261.492 | 2186.953 | 1534.432 | 37.403 | 47.555 | 46.765 | 39.172 | 1749.134 | 1862.822 | 87.649 | 70.280 | 2.113 | 2.093 | -0.075 | 0.065 | -9.362 | 8.383 | 9.362 | 8.383 |  |  |  |  |  |  |  |  |  |
| Plant6 | T2 | 1 | 53.831 | 41.575 | 748.611 | 56.000 | 59.000 | **0.039** | **0.295** | 2.169 | 17.425 | 109.831 | 100.575 | 998.386 | 459.882 | 1140.171 | 1510.863 | -31.597 | -21.445 | -33.766 | -38.870 | 1066.926 | 833.558 | 4.705 | 303.629 | 1.740 | 1.701 | 0.039 | 0.295 | 2.169 | 17.425 | 2.169 | 17.425 |  |  |  |  |  |  |  |  |  |
| Plant6 | T2 | 2 | 127.809 | 122.783 | 11245.524 | 126.000 | 131.000 | **0.014** | **0.063** | 1.809 | 8.217 | 253.809 | 253.783 | 1474.771 | 2555.823 | 1616.969 | 1792.491 | 38.403 | 50.555 | 40.212 | 42.338 | 1544.234 | 2140.394 | 3.272 | 67.524 | 2.103 | 2.103 | -0.014 | 0.063 | -1.809 | 8.217 | 1.809 | 8.217 |  |  |  |  |  |  |  |  |  |
| Plant6 | T2 | 3 | 132.271 | 116.747 | 10952.731 | 127.000 | 128.000 | **0.042** | **0.088** | 5.271 | 11.253 | 259.271 | 244.747 | 1552.576 | 2261.492 | 1995.781 | 1317.868 | 39.403 | 47.555 | 44.674 | 36.302 | 1760.285 | 1726.368 | 27.788 | 126.623 | 2.113 | 2.088 | -0.042 | 0.088 | -5.271 | 11.253 | 5.271 | 11.253 |  |  |  |  |  |  |  |  |  |
| Plant6 | T2 | 4 | 58.616 | 38.307 | 1983.870 | 60.000 | 34.000 | **0.023** | **0.127** | 1.384 | 4.307 | 118.616 | 72.307 | 761.608 | 2157.125 | 839.899 | 1775.564 | -27.597 | -46.445 | -28.981 | -42.137 | 799.796 | 1957.067 | 1.915 | 18.554 | 1.773 | 1.558 | 0.023 | -0.127 | 1.384 | -4.307 | 1.384 | 4.307 |  |  |  |  |  |  |  |  |  |
| Plant6 | T2 | 5 | 64.354 | 70.083 | 3291.749 | 62.000 | 72.000 | **0.038** | **0.027** | 2.354 | 1.917 | 126.354 | 142.083 | 655.219 | 71.316 | 540.257 | 107.367 | -25.597 | -8.445 | -23.243 | -10.362 | 594.968 | 87.504 | 5.540 | 3.675 | 1.801 | 1.852 | -0.038 | 0.027 | -2.354 | 1.917 | 2.354 | 1.917 |  |  |  |  |  |  |  |  |  |
| Plant6 | T2 | 6 | 104.122 | 96.726 | 6054.425 | 96.000 | 102.000 | **0.085** | **0.052** | 8.122 | 5.274 | 200.122 | 198.726 | 70.606 | 464.624 | 273.066 | 265.074 | 8.403 | 21.555 | 16.525 | 16.281 | 138.853 | 350.941 | 65.966 | 27.816 | 2.000 | 1.997 | -0.085 | 0.052 | -8.122 | 5.274 | 8.122 | 5.274 |  |  |  |  |  |  |  |  |  |
| Plant6 | T2 | 7 | 54.752 | 27.336 | 1572.004 | 55.000 | 32.000 | **0.005** | **0.146** | 0.248 | 4.664 | 109.752 | 59.336 | 1062.581 | 2346.904 | 1078.794 | 2820.507 | -32.597 | -48.445 | -32.845 | -53.108 | 1070.657 | 2572.831 | 0.061 | 21.749 | 1.739 | 1.472 | 0.005 | 0.146 | 0.248 | 4.664 | 0.248 | 4.664 |  |  |  |  |  |  |  |  |  |
| Plant6 | T2 | 8 | 100.582 | 94.787 | 6631.986 | 95.000 | 92.000 | **0.059** | **0.030** | 5.582 | 2.787 | 195.582 | 186.787 | 54.801 | 133.521 | 168.597 | 205.686 | 7.403 | 11.555 | 12.984 | 14.342 | 96.121 | 165.721 | 31.156 | 7.765 | 1.990 | 1.970 | -0.059 | -0.030 | -5.582 | -2.787 | 5.582 | 2.787 |  | **6013.955** |  |  |  |  |  |  |  |
| Plant6 | T3 | 0 | 116.890 | 115.469 | 7186.256 | 93.000 | 102.000 | **0.257** | **0.132** | 23.890 | 13.469 | 209.890 | 217.469 | 29.190 | 464.624 | 858.042 | 1226.721 | 5.403 | 21.555 | 29.292 | 35.025 | 158.259 | 754.960 | 570.714 | 181.426 | 2.021 | 2.036 | -0.257 | -0.132 | -23.890 | -13.469 | 23.890 | 13.469 |  |  |  |  |  |  |  |  |  |
| Plant6 | T3 | 1 | 135.994 | 95.350 | 4440.576 | 138.000 | 143.000 | **0.015** | **0.333** | 2.006 | 47.650 | 273.994 | 238.350 | 2540.437 | 3913.146 | 2342.267 | 222.177 | 50.403 | 62.555 | 48.397 | 14.906 | 2439.340 | 932.422 | 4.023 | 2270.479 | 2.137 | 2.076 | 0.015 | 0.333 | 2.006 | 47.650 | 2.006 | 47.650 |  |  |  |  |  |  |  |  |  |
| Plant6 | T3 | 2 | 137.342 | 123.299 | 11923.058 | 129.000 | 130.000 | **0.065** | **0.052** | 8.342 | 6.701 | 266.342 | 253.299 | 1714.187 | 2455.712 | 2474.554 | 1836.478 | 41.403 | 49.555 | 49.745 | 42.854 | 2059.575 | 2123.643 | 69.591 | 44.903 | 2.124 | 2.103 | -0.065 | 0.052 | -8.342 | 6.701 | 8.342 | 6.701 |  |  |  |  |  |  |  |  |  |
| Plant6 | T3 | 3 | 62.425 | 39.609 | 2098.264 | 60.000 | 33.000 | **0.040** | **0.200** | 2.425 | 6.609 | 122.425 | 72.609 | 761.608 | 2251.014 | 633.656 | 1667.539 | -27.597 | -47.445 | -25.173 | -40.836 | 694.692 | 1937.435 | 5.879 | 43.683 | 1.787 | 1.560 | -0.040 | -0.200 | -2.425 | -6.609 | 2.425 | 6.609 |  |  |  |  |  |  |  |  |  |
| Plant6 | T3 | 4 | 63.490 | 72.085 | 3503.047 | 64.000 | 72.000 | **0.008** | **0.001** | 0.510 | 0.085 | 127.490 | 144.085 | 556.830 | 71.316 | 581.176 | 69.882 | -23.597 | -8.445 | -24.108 | -8.360 | 568.873 | 70.595 | 0.260 | 0.007 | 1.804 | 1.858 | 0.008 | -0.001 | 0.510 | -0.085 | 0.510 | 0.085 |  |  |  |  |  |  |  |  |  |
| Plant6 | T3 | 5 | 57.233 | 35.602 | 1753.550 | 58.000 | 31.000 | **0.013** | **0.148** | 0.767 | 4.602 | 115.233 | 66.602 | 875.997 | 2444.794 | 922.011 | 2010.896 | -29.597 | -49.445 | -30.365 | -44.843 | 898.710 | 2217.256 | 0.589 | 21.177 | 1.761 | 1.522 | 0.013 | -0.148 | 0.767 | -4.602 | 0.767 | 4.602 |  |  |  |  |  |  |  |  |  |
| Plant6 | T3 | 6 | 89.084 | 107.228 | 9112.514 | 97.000 | 93.000 | **0.082** | **0.153** | 7.916 | 14.228 | 186.084 | 200.228 | 88.412 | 157.632 | 2.212 | 717.360 | 9.403 | 12.555 | 1.487 | 26.784 | 13.984 | 336.272 | 62.656 | 202.448 | 1.969 | 2.000 | 0.082 | -0.153 | 7.916 | -14.228 | 7.916 | 14.228 |  |  |  |  |  |  |  |  |  |
| Plant6 | T3 | 8 | 128.891 | 125.139 | 8607.208 | 118.000 | 126.000 | **0.092** | **0.007** | 10.891 | 0.861 | 246.891 | 251.139 | 924.327 | 2075.271 | 1705.134 | 1997.604 | 30.403 | 45.555 | 41.293 | 44.695 | 1255.429 | 2036.067 | 118.603 | 0.741 | 2.091 | 2.099 | -0.092 | 0.007 | -10.891 | 0.861 | 10.891 | 0.861 |  |  |  |  |  |  |  |  |  |
| Plant6 | T3 | 9 | 139.711 | 122.587 | 12389.620 | 133.000 | 130.000 | **0.050** | **0.057** | 6.711 | 7.413 | 272.711 | 252.587 | 2061.409 | 2455.712 | 2715.867 | 1775.924 | 45.403 | 49.555 | 52.114 | 42.142 | 2366.118 | 2088.339 | 45.041 | 54.959 | 2.135 | 2.101 | -0.050 | 0.057 | -6.711 | 7.413 | 6.711 | 7.413 |  | **6779.344** |  |  |  |  |  |  |  |
| **Averages** |  |  | **88.967** | **78.890** | **5696.004** | **86.228** | **82.000** | **0.05748** | **0.088** | **12.857** | **62.016** | **175.195** | **160.890** | **808.696** | **1441.163** | **970.006** | **1142.634** | **-1.369** | **1.555** | **1.369** | **-1.555** | **862.804** | **1244.790** | **53.093** | **94.217** | **1.918** | **1.855** | **-0.026** | **0.009** | **-2.739** | **3.110** | **4.842** | **6.768** | **#DIV/0!** |  |  |  |  |  |  |  |  |
| **STDEV** |  |  | 31.056 | 33.797 | 3918.099 | 28.347 | 37.972 | **0.06400** | **0.08357** |  |  |  |  |  |  |  |  |  |  |  |  | x | x |  |  |  |  |  |  |  |  |  |  |  |  |  |  |  |  |  |  |  |
| **Min** |  |  | 39.641 | 27.336 | 678.901 | 36.000 | 29.000 | 0.001 | 0.001 |  | 5.114 | 6.927 |  |  |  |  |  |  |  |  |  | 475.595 | 392.277 |  |  |  |  |  |  |  |  |  |  |  |  |  |  |  |  |  |  |  |
| **Max** |  |  | 160.716 | 148.529 | 16848.045 | 165.000 | 161.000 | 0.425 | 0.723 |  |  |  |  |  |  |  |  |  |  |  |  |  |  |  |  |  |  |  |  |  |  |  |  |  |  |  |  |  |  |  |  |  |
|  |  |  |  |  |  |  |  |  |  |  |  |  |  |  |  |  |  |  |  |  |  |  |  |  |  |  |  |  |  |  |  |  |  |  |  |  |  |  |  |  |  |  |
|  |  |  |  |  | **Leaf width** | **Leaf Length** | **Unbiased Width** | **Unbiased Length** |  |  | **Leaf Width** | **Leaf Length** |  |  |  |  |  |  |  |  |  |  |  |  |  |  |  |  |  |  |  |  |  |  |  |  |  |  |  |  |  |  |
|  |  |  |  | **x\_bar** | 87.59725 | 80.44486 |  |  |  | **RMSE** | 7.2865 | 9.7065 |  |  |  |  |  |  |  |  |  |  |  |  |  |  |  |  |  |  |  |  |  |  |  |  |  |  |  |  |  |  |
|  |  |  |  | **S\_squre** | 889.35093 | 1291.89857 | 891.82823 | 1295.49717 |  | **Normalized RMSE** | 0.0602 | 0.0801 |  |  |  |  |  |  |  |  |  |  |  |  |  |  |  |  |  |  |  |  |  |  |  |  |  |  |  |  |  |  |
|  |  |  |  | **ICC** | **0.97015** | **0.96354** | **0.97286** | **0.96623** |  |  |  |  |  |  |  |  |  |  |  |  |  |  |  |  |  |  |  |  |  |  |  |  |  |  |  |  |  |  |  |  |  |  |
|  |  |  |  | **Peasron** | **0.97836** | **0.97376** |  |  |  |  |  |  |  |  |  |  |  |  |  |  |  |  |  |  |  |  |  |  |  |  |  |  |  |  |  |  |  |  |  |  |  |  |
|  |  |  |  | **(Pearson)^2** | **0.95719** | **0.94822** |  |  |  |  |  |  |  |  |  |  |  |  |  |  |  |  |  |  |  |  |  |  |  |  |  |  |  |  |  |  |  |  |  |  |  |  |
|  |  |  |  |  |  |  |  |  |  |  |  |  |  |  |  |  |  |  |  |  |  |  |  |  |  |  |  |  |  |  |  |  |  |  |  |  |  |  |  |  |  |  |
| **Main stem data** |  |  |  |  |  |  |  |  |  |  |  |  |  |  |  |  |  |  |  |  |  |  |  |  |  |  |  |  |  |  |  |  |  |  |  |  |  |  |  |  |  |  |
|  |  |  |  |  |  |  | **Values used to compute the ICC** |  |  |  | **RMSE** | **Bland/Altman** |  | **MBE** |  |  |  |  |  |  |  |  |  |  |  |  |  |  |  |  |  |  |  |  |  |  |  |  |  |  |  |  |
| **Plant ID** | **TP** | **Aut** | **Man** | **Error** | **x1+x2** | **x1 - x\_bar** | **x2 - x\_bar** | **(x1 – x\_bar)^2** | **(x2 – x\_bar)^2** | **X\*X** | **(m – a)^2** | **log((m+a)/2)** | **(a-m)/m** | **(a-m)** |  |  |  |  |  |  |  |  |  |  |  |  |  |  |  |  |  |  |  |  |  |  |  |  |  |  |  |  |
| Plant1 | T0 | 142.775 | 122.000 | 0.170 | 264.775 | -43.969 | -23.194 | 1933.286 | 537.962 | 1019.821 | 431.606 | 2.122 | -0.170 | 20.775 |  |  |  |  |  |  |  |  |  |  |  |  |  |  |  |  |  |  |  |  |  |  |  |  |  |  |  |  |
| Plant1 | T1 | 167.752 | 168.000 | 0.001 | 335.752 | 2.031 | 1.783 | 4.124 | 3.179 | 3.621 | 0.061 | 2.225 | 0.001 | -0.248 |  |  |  |  |  |  |  |  |  |  |  |  |  |  |  |  |  |  |  |  |  |  |  |  |  |  |  |  |
| Plant1 | T2 | 222.978 | 218.000 | 0.023 | 440.978 | 52.031 | 57.009 | 2707.209 | 3250.060 | 2966.242 | 24.785 | 2.343 | -0.023 | 4.978 |  |  |  |  |  |  |  |  |  |  |  |  |  |  |  |  |  |  |  |  |  |  |  |  |  |  |  |  |
| Plant1 | T3 | 259.328 | 242.000 | 0.072 | 501.328 | 76.031 | 93.359 | 5780.690 | 8715.866 | 7098.149 | 300.258 | 2.399 | -0.072 | 17.328 |  |  |  |  |  |  |  |  |  |  |  |  |  |  |  |  |  |  |  |  |  |  |  |  |  |  |  |  |
| Plant2 | T0 | 182.391 | 124.000 | 0.471 | 306.391 | -41.969 | 16.422 | 1761.410 | 269.669 | -689.201 | 3409.480 | 2.185 | -0.471 | 58.391 |  |  |  |  |  |  |  |  |  |  |  |  |  |  |  |  |  |  |  |  |  |  |  |  |  |  |  |  |
| Plant2 | T1 | 228.391 | 180.000 | 0.269 | 408.391 | 14.031 | 62.422 | 196.865 | 3896.456 | 875.828 | 2341.664 | 2.310 | -0.269 | 48.391 |  |  |  |  |  |  |  |  |  |  |  |  |  |  |  |  |  |  |  |  |  |  |  |  |  |  |  |  |
| Plant2 | T2 | 232.538 | 229.000 | 0.015 | 461.538 | 63.031 | 66.568 | 3972.888 | 4431.353 | 4195.863 | 12.514 | 2.363 | -0.015 | 3.538 |  |  |  |  |  |  |  |  |  |  |  |  |  |  |  |  |  |  |  |  |  |  |  |  |  |  |  |  |
| Plant2 | T3 | 257.216 | 250.000 | 0.029 | 507.216 | 84.031 | 91.247 | 7061.184 | 8325.943 | 7667.530 | 52.067 | 2.404 | -0.029 | 7.216 |  |  |  |  |  |  |  |  |  |  |  |  |  |  |  |  |  |  |  |  |  |  |  |  |  |  |  |  |
| Plant3 | T0 | 130.848 | 100.000 | 0.308 | 230.848 | -65.969 | -35.122 | 4351.929 | 1233.520 | 2316.936 | 951.577 | 2.062 | -0.308 | 30.848 |  |  |  |  |  |  |  |  |  |  |  |  |  |  |  |  |  |  |  |  |  |  |  |  |  |  |  |  |
| Plant3 | T1 | 139.299 | 140.000 | 0.005 | 279.299 | -25.969 | -26.670 | 674.397 | 711.301 | 692.603 | 0.491 | 2.145 | 0.005 | -0.701 |  |  |  |  |  |  |  |  |  |  |  |  |  |  |  |  |  |  |  |  |  |  |  |  |  |  |  |  |
| Plant3 | T2 | 169.609 | 175.000 | 0.031 | 344.609 | 9.031 | 3.640 | 81.556 | 13.251 | 32.874 | 29.060 | 2.236 | 0.031 | -5.391 |  |  |  |  |  |  |  |  |  |  |  |  |  |  |  |  |  |  |  |  |  |  |  |  |  |  |  |  |
| Plant3 | T3 | 193.353 | 193.000 | 0.002 | 386.353 | 27.031 | 27.384 | 730.667 | 749.868 | 740.205 | 0.125 | 2.286 | -0.002 | 0.353 |  |  |  |  |  |  |  |  |  |  |  |  |  |  |  |  |  |  |  |  |  |  |  |  |  |  |  |  |
| Plant4 | T0 | 108.225 | 93.000 | 0.164 | 201.225 | -72.969 | -57.744 | 5324.497 | 3334.374 | 4213.534 | 231.804 | 2.003 | -0.164 | 15.225 |  |  |  |  |  |  |  |  |  |  |  |  |  |  |  |  |  |  |  |  |  |  |  |  |  |  |  |  |
| Plant4 | T1 | 132.766 | 125.000 | 0.062 | 257.766 | -40.969 | -33.204 | 1678.471 | 1102.482 | 1360.325 | 60.303 | 2.110 | -0.062 | 7.766 |  |  |  |  |  |  |  |  |  |  |  |  |  |  |  |  |  |  |  |  |  |  |  |  |  |  |  |  |
| Plant4 | T2 | 166.184 | 166.000 | 0.001 | 332.184 | 0.031 | 0.215 | 0.001 | 0.046 | 0.007 | 0.034 | 2.220 | -0.001 | 0.184 |  |  |  |  |  |  |  |  |  |  |  |  |  |  |  |  |  |  |  |  |  |  |  |  |  |  |  |  |
| Plant4 | T3 | 189.535 | 180.000 | 0.053 | 369.535 | 14.031 | 23.565 | 196.865 | 555.329 | 330.643 | 90.908 | 2.267 | -0.053 | 9.535 |  |  |  |  |  |  |  |  |  |  |  |  |  |  |  |  |  |  |  |  |  |  |  |  |  |  |  |  |
| Plant5 | T0 | 88.355 | 76.000 | 0.163 | 164.355 | -89.969 | -77.614 | 8094.448 | 6024.010 | 6982.910 | 152.638 | 1.915 | -0.163 | 12.355 |  |  |  |  |  |  |  |  |  |  |  |  |  |  |  |  |  |  |  |  |  |  |  |  |  |  |  |  |
| Plant5 | T1 | 106.465 | 105.000 | 0.014 | 211.465 | -60.969 | -59.504 | 3717.237 | 3540.717 | 3627.904 | 2.147 | 2.024 | -0.014 | 1.465 |  |  |  |  |  |  |  |  |  |  |  |  |  |  |  |  |  |  |  |  |  |  |  |  |  |  |  |  |
| Plant5 | T2 | 135.922 | 146.000 | 0.069 | 281.922 | -19.969 | -30.047 | 398.767 | 902.826 | 600.014 | 101.564 | 2.149 | 0.069 | -10.078 |  |  |  |  |  |  |  |  |  |  |  |  |  |  |  |  |  |  |  |  |  |  |  |  |  |  |  |  |
| Plant5 | T3 | 152.929 | 165.000 | 0.073 | 317.929 | -0.969 | -13.041 | 0.939 | 170.058 | 12.638 | 145.721 | 2.201 | 0.073 | -12.071 |  |  |  |  |  |  |  |  |  |  |  |  |  |  |  |  |  |  |  |  |  |  |  |  |  |  |  |  |
| Plant6 | T0 | 121.779 | 112.000 | 0.087 | 233.779 | -53.969 | -44.190 | 2912.669 | 1952.771 | 2384.906 | 95.628 | 2.068 | -0.087 | 9.779 |  |  |  |  |  |  |  |  |  |  |  |  |  |  |  |  |  |  |  |  |  |  |  |  |  |  |  |  |
| Plant6 | T1 | 152.180 | 141.000 | 0.079 | 293.180 | -24.969 | -13.790 | 623.458 | 190.153 | 344.315 | 124.982 | 2.166 | -0.079 | 11.180 |  |  |  |  |  |  |  |  |  |  |  |  |  |  |  |  |  |  |  |  |  |  |  |  |  |  |  |  |
| Plant6 | T2 | 188.568 | 195.000 | 0.033 | 383.568 | 29.031 | 22.599 | 842.790 | 510.723 | 656.074 | 41.366 | 2.283 | 0.033 | -6.432 |  |  |  |  |  |  |  |  |  |  |  |  |  |  |  |  |  |  |  |  |  |  |  |  |  |  |  |  |
| Plant6 | T3 | 231.136 | 221.000 | 0.046 | 452.136 | 55.031 | 65.166 | 3028.394 | 4246.665 | 3586.165 | 102.730 | 2.354 | -0.046 | 10.136 |  |  |  |  |  |  |  |  |  |  |  |  |  |  |  |  |  |  |  |  |  |  |  |  |  |  |  |  |
| **Averages** |  | **170.855** | **161.083** | **0.093** | **9.34%** |  |  |  |  | **Average** | **362.646** |  |  | **9.77163308333333** | **MBE** |  |  |  |  |  |  |  |  |  |  |  |  |  |  |  |  |  |  |  |  |  |  |  |  |  |  |  |
| **STDEV** |  |  |  | **0.115** | **11.50%** |  |  |  |  | **Range** | 183.328 |  |  |  |  |  |  |  |  |  |  |  |  |  |  |  |  |  |  |  |  |  |  |  |  |  |  |  |  |  |  |  |
|  |  |  |  |  | **15.949** | **Range** |  |  |  |  |  |  |  |  |  |  |  |  |  |  |  |  |  |  |  |  |  |  |  |  |  |  |  |  |  |  |  |  |  |  |  |  |
|  |  |  |  |  |  |  |  |  |  |  |  |  |  |  |  |  |  |  |  |  |  |  |  |  |  |  |  |  |  |  |  |  |  |  |  |  |  |  |  |  |  |  |
|  |  |  |  |  |  |  |  |  |  |  |  |  |  |  |  |  |  |  |  |  |  |  |  |  |  |  |  |  |  |  |  |  |  |  |  |  |  |  |  |  |  |  |
|  |  |  |  |  |  |  |  |  |  |  |  |  |  |  |  |  |  |  |  |  |  |  |  |  |  |  |  |  |  |  |  |  |  |  |  |  |  |  |  |  |  |  |
|  |  |  |  |  |  |  |  |  |  |  |  |  |  |  |  |  |  |  |  |  |  |  |  |  |  |  |  |  |  |  |  |  |  |  |  |  |  |  |  |  |  |  |
|  |  |  |  |  |  |  |  |  |  |  |  |  |  |  |  |  |  |  |  |  |  |  |  |  |  |  |  |  |  |  |  |  |  |  |  |  |  |  |  |  |  |  |
|  |  |  |  |  |  |  |  |  |  |  |  |  |  |  |  |  |  |  |  |  |  |  |  |  |  |  |  |  |  |  |  |  |  |  |  |  |  |  |  |  |  |  |
|  |  |  |  |  | **Main Stem Height** | **Unbiased** |  | **RMSE** | 19.043 |  |  |  |  |  |  |  |  |  |  |  |  |  |  |  |  |  |  |  |  |  |  |  |  |  |  |  |  |  |  |  |  |  |
|  |  |  |  | **x\_bar** | 165.969 |  |  | **NRMSE** | 0.104 |  |  |  |  |  |  |  |  |  |  |  |  |  |  |  |  |  |  |  |  |  |  |  |  |  |  |  |  |  |  |  |  |  |
|  |  |  |  | **S\_square** | 2307.153 | 2356.241 |  |  |  |  |  |  |  |  |  |  |  |  |  |  |  |  |  |  |  |  |  |  |  |  |  |  |  |  |  |  |  |  |  |  |  |  |
|  |  |  |  | **ICC** | **0.921** | **0.941** |  |  |  |  |  |  |  |  |  |  |  |  |  |  |  |  |  |  |  |  |  |  |  |  |  |  |  |  |  |  |  |  |  |  |  |  |
|  |  |  |  | **(Pearson Cor)** | **0.942** |  |  |  |  |  |  |  |  |  |  |  |  |  |  |  |  |  |  |  |  |  |  |  |  |  |  |  |  |  |  |  |  |  |  |  |  |  |
|  |  |  |  | **(Pearson Cor)^2** | **0.887** |  |  |  |  |  |  |  |  |  |  |  |  |  |  |  |  |  |  |  |  |  |  |  |  |  |  |  |  |  |  |  |  |  |  |  |  |  |
|  |  |  |  |  |  |  |  |  |  |  |  |  |  |  |  |  |  |  |  |  |  |  |  |  |  |  |  |  |  |  |  |  |  |  |  |  |  |  |  |  |  |  |
|  |  |  |  |  |  |  |  |  |  |  |  |  |  |  |  |  |  |  |  |  |  |  |  |  |  |  |  |  |  |  |  |  |  |  |  |  |  |  |  |  |  |  |
|  |  |  |  |  |  |  |  |  |  |  |  |  |  |  |  |  |  |  |  |  |  |  |  |  |  |  |  |  |  |  |  |  |  |  |  |  |  |  |  |  |  |  |
|  |  |  |  |  |  |  |  |  |  |  |  |  |  |  |  |  |  |  |  |  |  |  |  |  |  |  |  |  |  |  |  |  |  |  |  |  |  |  |  |  |  |  |
| **NUMBER OF LEAF** | **T0** | **T1** | **T2** | **T3** |  |  |  |  |  |  |  |  |  |  |  |  |  |  |  |  |  |  |  |  |  |  |  |  |  |  |  |  |  |  |  |  |  |  |  |  |  |  |
| **Plant1** | 7 | 8 | 10 | 12 |  |  |  |  |  |  |  |  |  |  |  |  |  |  |  |  |  |  |  |  |  |  |  |  |  |  |  |  |  |  |  |  |  |  |  |  |  |  |
| **Plant2** | 9 | 14 | 14 | 13 |  |  |  |  |  |  |  |  |  |  |  |  |  |  |  |  |  |  |  |  |  |  |  |  |  |  |  |  |  |  |  |  |  |  |  |  |  |  |
| **Plant3** | 6 | 7 | 8 | 11 |  |  |  |  |  |  |  |  |  |  |  |  |  |  |  |  |  |  |  |  |  |  |  |  |  |  |  |  |  |  |  |  |  |  |  |  |  |  |
| **Plant4** | 5 | 7 | 7 | 9 |  |  |  |  |  |  |  |  |  |  |  |  |  |  |  |  |  |  |  |  |  |  |  |  |  |  |  |  |  |  |  |  |  |  |  |  |  |  |
| **Plant5** | 5 | 6 | 8 | 9 |  |  |  |  |  |  |  |  |  |  |  |  |  |  |  |  |  |  |  |  |  |  |  |  |  |  |  |  |  |  |  |  |  |  |  |  |  |  |
| **Plant6** | 6 | 7 | 9 | 10 |  |  |  |  |  |  |  |  |  |  |  |  |  |  |  |  |  |  |  |  |  |  |  |  |  |  |  |  |  |  |  |  |  |  |  |  |  |  |
|  |  |  |  |  |  |  |  |  |  |  |  |  |  |  |  |  |  |  |  |  |  |  |  |  |  |  |  |  |  |  |  |  |  |  |  |  |  |  |  |  |  |  |
|  |  |  |  |  |  |  |  |  |  |  |  |  |  |  |  |  |  |  |  |  |  |  |  |  |  |  |  |  |  |  |  |  |  |  |  |  |  |  |  |  |  |  |
|  |  |  |  |  |  |  |  |  |  |  |  |  |  |  |  |  |  |  |  |  |  |  |  |  |  |  |  |  |  |  |  |  |  |  |  |  |  |  |  |  |  |  |
| **CPU Time** |  |  |  |  |  |  |  |  |  |  |  |  |  |  |  |  |  |  |  |  |  |  |  |  |  |  |  |  |  |  |  |  |  |  |  |  |  |  |  |  |  |  |
|  |  |  |  |  |  |  |  |  |  |  |  |  |  |  |  |  |  |  |  |  |  |  |  |  |  |  |  |  |  |  |  |  |  |  |  |  |  |  |  |  |  |  |
|  |  |  |  |  |  |  |  |  |  |  |  |  |  |  |  |  |  |  |  |  |  |  |  |  |  |  |  |  |  |  |  |  |  |  |  |  |  |  |  |  |  |  |
| **Segmentation** |  |  |  |  |  |  |  | **Segmentation** |  |  |  |  |  | **Data extraction** |  |  |  |  |  |  |  |  |  |  |  |  |  |  |  |  |  |  |  |  |  |  |  |  |  |  |  |  |
| **Time Point** | **Plant1** | **Plant2** | **Plant3** | **Plant4** | **Plant5** | **Plant6** |  | **Run 1** | **Run 2** | **Run 3** | **Average (sec)** |  |  | **Run 1** | **Run 2** | **Run 3** | **Average (sec)** |  |  |  |  |  |  |  |  |  |  |  |  |  |  |  |  |  |  |  |  |  |  |  |  |  |
| T0 | 0.63 | 0.65 | 0.69 | 0.68 | 0.61 | 0.62 |  | 37.25 | 37.74 | 37.74 | 37.58 |  |  | 4.52 | 4.44 | 4.47 | 4.47667 |  |  |  |  |  |  |  |  |  |  |  |  |  |  |  |  |  |  |  |  |  |  |  |  |  |
| T1 | 0.64 | 0.62 | 0.56 | 0.55 | 0.55 | 0.82 |  | 37.92 | 38.31 | 38.98 | 38.40 |  |  | 4.02 | 4.09 | 4.21 | 4.10667 |  |  |  |  |  |  |  |  |  |  |  |  |  |  |  |  |  |  |  |  |  |  |  |  |  |
| T2 | 0.71 | 0.68 | 0.52 | 0.68 | 0.64 | 0.59 |  | 42.54 | 42.91 | 42.61 | 42.69 |  |  | 4.33 | 4.37 | 4.44 | 4.38000 |  |  |  |  |  |  |  |  |  |  |  |  |  |  |  |  |  |  |  |  |  |  |  |  |  |
| T3 | 0.68 | 0.61 | 0.67 | 0.80 | 0.70 | 0.81 |  | 40.72 | 41.16 | 40.78 | 40.89 |  |  | 4.37 | 4.39 | 4.33 | 4.36333 |  |  |  |  |  |  |  |  |  |  |  |  |  |  |  |  |  |  |  |  |  |  |  |  |  |
| T0 (sec) | 37.58 | 38.96 | 41.16 | 40.74 | 36.43 | 37.00 |  | 38.7 | 39.24 | 38.93 | 38.96 |  |  | 3.9 | 3.89 | 3.92 | 3.90333 |  |  |  |  |  |  |  |  |  |  |  |  |  |  |  |  |  |  |  |  |  |  |  |  |  |
| T1 (sec) | 38.40 | 36.98 | 33.76 | 32.80 | 32.85 | 49.32 |  | 36.97 | 36.87 | 37.1 | 36.98 |  |  | 3.96 | 3.98 | 3.96 | 3.96667 |  |  |  |  |  |  |  |  |  |  |  |  |  |  |  |  |  |  |  |  |  |  |  |  |  |
| T2 (sec) | 42.69 | 40.84 | 30.92 | 40.69 | 38.57 | 35.48 |  | 40.82 | 40.96 | 40.75 | 40.84 |  |  | 4.08 | 4.12 | 4.01 | 4.07000 |  |  |  |  |  |  |  |  |  |  |  |  |  |  |  |  |  |  |  |  |  |  |  |  |  |
| T3 (sec) | 40.89 | 36.77 | 40.31 | 48.19 | 42.11 | 48.52 |  | 36.7 | 36.84 | 36.76 | 36.77 |  |  | 4.29 | 4.27 | 4.35 | 4.30333 |  |  |  |  |  |  |  |  |  |  |  |  |  |  |  |  |  |  |  |  |  |  |  |  |  |
|  |  |  |  |  | **Max (sec)** | **49.3166666666667** |  | 41.17 | 41.07 | 41.25 | 41.16 |  |  | 4.93 | 4.85 | 4.91 | 4.89667 |  |  |  |  |  |  |  |  |  |  |  |  |  |  |  |  |  |  |  |  |  |  |  |  |  |
|  |  |  |  |  | **Average (sec)** | **39.2475** |  | 33.78 | 33.83 | 33.67 | 33.76 |  |  | 4.51 | 4.4 | 4.38 | 4.43000 |  |  |  |  |  |  |  |  |  |  |  |  |  |  |  |  |  |  |  |  |  |  |  |  |  |
|  |  |  |  |  |  |  |  | 30.67 | 31.18 | 30.9 | 30.92 |  |  | 3.68 | 3.6 | 3.77 | 3.68333 |  |  |  |  |  |  |  |  |  |  |  |  |  |  |  |  |  |  |  |  |  |  |  |  |  |
|  |  |  |  |  |  |  |  | 39.89 | 40.44 | 40.6 | 40.31 |  |  | 4.24 | 4.26 | 4.25 | 4.25000 |  |  |  |  |  |  |  |  |  |  |  |  |  |  |  |  |  |  |  |  |  |  |  |  |  |
|  |  |  |  |  |  |  |  | 40.99 | 40.57 | 40.65 | 40.74 |  |  | 4.36 | 4.35 | 4.37 | 4.36000 |  |  |  |  |  |  |  |  |  |  |  |  |  |  |  |  |  |  |  |  |  |  |  |  |  |
|  |  |  |  |  |  |  |  | 30.87 | 36.38 | 31.16 | 32.80 |  |  | 3.72 | 3.75 | 3.99 | 3.82000 |  |  |  |  |  |  |  |  |  |  |  |  |  |  |  |  |  |  |  |  |  |  |  |  |  |
|  |  |  |  |  |  |  |  | 40.07 | 40.23 | 41.77 | 40.69 |  |  | 3.8 | 3.8 | 3.82 | 3.80667 |  |  |  |  |  |  |  |  |  |  |  |  |  |  |  |  |  |  |  |  |  |  |  |  |  |
|  |  |  |  |  |  |  |  | 48.22 | 48.1 | 48.24 | 48.19 |  |  | 4.57 | 4.7 | 4.61 | 4.62667 |  |  |  |  |  |  |  |  |  |  |  |  |  |  |  |  |  |  |  |  |  |  |  |  |  |
|  |  |  |  |  |  |  |  | 36.25 | 36.49 | 36.56 | 36.43 |  |  | 4.44 | 4.44 | 4.69 | 4.52333 |  |  |  |  |  |  |  |  |  |  |  |  |  |  |  |  |  |  |  |  |  |  |  |  |  |
| **Data extraction** |  |  |  |  |  |  |  | 32.7 | 32.94 | 32.92 | 32.85 |  |  | 4.43 | 4.42 | 4.4 | 4.41667 |  |  |  |  |  |  |  |  |  |  |  |  |  |  |  |  |  |  |  |  |  |  |  |  |  |
| **Time Point** | **Plant1** | **Plant2** | **Plant3** | **Plant4** | **Plant5** | **Plant6** |  | 38.52 | 38.75 | 38.45 | 38.57 |  |  | 4.06 | 4.01 | 4.19 | 4.08667 |  |  |  |  |  |  |  |  |  |  |  |  |  |  |  |  |  |  |  |  |  |  |  |  |  |
| T0 | 0.0753 | 0.0650 | 0.0822 | 0.0727 | 0.0740 | 0.0767 |  | 42 | 42.04 | 42.28 | 42.11 |  |  | 4.26 | 4.22 | 4.24 | 4.24000 |  |  |  |  |  |  |  |  |  |  |  |  |  |  |  |  |  |  |  |  |  |  |  |  |  |
| T1 | 0.0670 | 0.0660 | 0.0752 | 0.0620 | 0.0738 | 0.0740 |  | 36.84 | 37.11 | 37.04 | 37.00 |  |  | 4.6 | 4.5 | 4.63 | 4.57667 |  |  |  |  |  |  |  |  |  |  |  |  |  |  |  |  |  |  |  |  |  |  |  |  |  |
| T2 | 0.0722 | 0.0680 | 0.0613 | 0.0633 | 0.0677 | 0.0665 |  | 50.07 | 48.89 | 48.99 | 49.32 |  |  | 4.44 | 4.48 | 4.45 | 4.45667 |  |  |  |  |  |  |  |  |  |  |  |  |  |  |  |  |  |  |  |  |  |  |  |  |  |
| T3 | 0.0728 | 0.0715 | 0.0707 | 0.0762 | 0.0710 | 0.0710 |  | 35.41 | 35.5 | 35.52 | 35.48 |  |  | 3.99 | 3.94 | 3.96 | 3.96333 |  |  |  |  |  |  |  |  |  |  |  |  |  |  |  |  |  |  |  |  |  |  |  |  |  |
| T0 (sec) | 4.52 | 3.9 | 4.93 | 4.36 | 4.44 | 4.6 |  | 48.33 | 48.96 | 48.26 | 48.52 |  |  | 4.26 | 4.27 | 4.22 | 4.25000 |  |  |  |  |  |  |  |  |  |  |  |  |  |  |  |  |  |  |  |  |  |  |  |  |  |
| T1 (sec) | 4.02 | 3.96 | 4.51 | 3.72 | 4.43 | 4.44 |  |  |  |  |  |  |  |  |  |  |  |  |  |  |  |  |  |  |  |  |  |  |  |  |  |  |  |  |  |  |  |  |  |  |  |  |
| T2 (sec) | 4.33 | 4.08 | 3.68 | 3.8 | 4.06 | 3.99 |  |  |  |  |  |  |  |  |  |  |  |  |  |  |  |  |  |  |  |  |  |  |  |  |  |  |  |  |  |  |  |  |  |  |  |  |
| T3 (sec) | 4.37 | 4.29 | 4.24 | 4.57 | 4.26 | 4.26 |  |  |  |  |  |  |  |  |  |  |  |  |  |  |  |  |  |  |  |  |  |  |  |  |  |  |  |  |  |  |  |  |  |  |  |  |
|  |  |  |  |  | **Max (sec)** | **4.93** |  |  |  |  |  |  |  |  |  |  |  |  |  |  |  |  |  |  |  |  |  |  |  |  |  |  |  |  |  |  |  |  |  |  |  |  |
|  |  |  |  |  | **Average (sec)** | **4.24** |  |  |  |  |  |  |  |  |  |  |  |  |  |  |  |  |  |  |  |  |  |  |  |  |  |  |  |  |  |  |  |  |  |  |  |  |
|  |  |  |  |  |  |  |  |  |  |  |  |  |  |  |  |  |  |  |  |  |  |  |  |  |  |  |  |  |  |  |  |  |  |  |  |  |  |  |  |  |  |  |
|  |  |  |  |  |  |  |  |  |  |  |  |  |  |  |  |  |  |  |  |  |  |  |  |  |  |  |  |  |  |  |  |  |  |  |  |  |  |  |  |  |  |  |
|  |  |  |  |  |  |  |  |  |  |  |  |  |  |  |  |  |  |  |  |  |  |  |  |  |  |  |  |  |  |  |  |  |  |  |  |  |  |  |  |  |  |  |
|  |  |  |  |  |  |  |  |  |  |  |  |  |  |  |  |  |  |  |  |  |  |  |  |  |  |  |  |  |  |  |  |  |  |  |  |  |  |  |  |  |  |  |
|  |  |  |  |  |  |  |  |  |  |  |  |  |  |  |  |  |  |  |  |  |  |  |  |  |  |  |  |  |  |  |  |  |  |  |  |  |  |  |  |  |  |  |
|  |  |  |  |  |  |  |  |  |  |  |  |  |  |  |  |  |  |  |  |  |  |  |  |  |  |  |  |  |  |  |  |  |  |  |  |  |  |  |  |  |  |  |
| **Longitudinal Matching** |  |  |  |  | **Full analysis** |  |  |  |  |  |  |  |  |  |  |  |  |  |  |  |  |  |  |  |  |  |  |  |  |  |  |  |  |  |  |  |  |  |  |  |  |  |
| **Time Point** | **Time (min)** | **Time (sec)** |  |  | **Run 1** | **Run 2** | **Run 3** | **Average (sec)** |  |  |  |  |  |  |  |  |  |  |  |  |  |  |  |  |  |  |  |  |  |  |  |  |  |  |  |  |  |  |  |  |  |  |
| 1 | 2.213 | **132.4** |  | **Plant1** | 132.69 | 132.73 | 132.83 | 132.75 |  |  |  |  |  |  |  |  |  |  |  |  |  |  |  |  |  |  |  |  |  |  |  |  |  |  |  |  |  |  |  |  |  |  |
| 2 | 2.519 | **150.75** |  | **Plant2** | 150.84 | 151.15 | 151.46 | 151.15 |  |  |  |  |  |  |  |  |  |  |  |  |  |  |  |  |  |  |  |  |  |  |  |  |  |  |  |  |  |  |  |  |  |  |
| 3 | 2.156 | **129.33** |  | **Plant3** | 128.65 | 129.19 | 130.21 | 129.35 |  |  |  |  |  |  |  |  |  |  |  |  |  |  |  |  |  |  |  |  |  |  |  |  |  |  |  |  |  |  |  |  |  |  |
| 4 | 2.018 | **120.36** |  | **Plant4** | 121.00 | 120.84 | 121.40 | 121.08 |  |  |  |  |  |  |  |  |  |  |  |  |  |  |  |  |  |  |  |  |  |  |  |  |  |  |  |  |  |  |  |  |  |  |
| 5 | 2.053 | **123.14** |  | **Plant5** | 122.77 | 123.58 | 123.18 | 123.18 |  |  |  |  |  |  |  |  |  |  |  |  |  |  |  |  |  |  |  |  |  |  |  |  |  |  |  |  |  |  |  |  |  |  |
| 6 | 2.118 | **127.23** |  | **Plant6** | 127.09 | 127.21 | 127.02 | 127.11 |  |  |  |  |  |  |  |  |  |  |  |  |  |  |  |  |  |  |  |  |  |  |  |  |  |  |  |  |  |  |  |  |  |  |
| **Max** | **2.51916666666667** |  |  |  |  |  |  |  |  |  |  |  |  |  |  |  |  |  |  |  |  |  |  |  |  |  |  |  |  |  |  |  |  |  |  |  |  |  |  |  |  |  |
| **Averages** | **2.17948148148148** |  |  |  |  |  |  |  |  |  |  |  |  |  |  |  |  |  |  |  |  |  |  |  |  |  |  |  |  |  |  |  |  |  |  |  |  |  |  |  |  |  |
|  |  |  |  |  |  |  |  |  |  |  |  |  |  |  |  |  |  |  |  |  |  |  |  |  |  |  |  |  |  |  |  |  |  |  |  |  |  |  |  |  |  |  |
|  |  |  |  |  |  |  |  |  |  |  |  |  |  |  |  |  |  |  |  |  |  |  |  |  |  |  |  |  |  |  |  |  |  |  |  |  |  |  |  |  |  |  |
|  |  |  |  |  |  |  |  |  |  |  |  |  |  |  |  |  |  |  |  |  |  |  |  |  |  |  |  |  |  |  |  |  |  |  |  |  |  |  |  |  |  |  |
|  |  |  |  |  |  |  |  |  |  |  |  |  |  |  |  |  |  |  |  |  |  |  |  |  |  |  |  |  |  |  |  |  |  |  |  |  |  |  |  |  |  |  |
|  |  |  |  |  |  |  |  |  |  |  |  |  |  |  |  |  |  |  |  |  |  |  |  |  |  |  |  |  |  |  |  |  |  |  |  |  |  |  |  |  |  |  |
|  |  |  |  |  |  |  |  |  |  |  |  |  |  |  |  |  |  |  |  |  |  |  |  |  |  |  |  |  |  |  |  |  |  |  |  |  |  |  |  |  |  |  |
| **Full analysis** |  |  |  |  |  |  |  |  |  |  |  |  |  |  |  |  |  |  |  |  |  |  |  |  |  |  |  |  |  |  |  |  |  |  |  |  |  |  |  |  |  |  |
| **CPU** | **Time (min)** | **Time (sec)** |  |  | **Full analysis** |  |  |  |  |  |  |  |  |  |  |  |  |  |  |  |  |  |  |  |  |  |  |  |  |  |  |  |  |  |  |  |  |  |  |  |  |  |
| 1 | 4.9649 | 297.893333333333 |  |  | **Run 1** | **Run 2** | **Run 3** | **Average (sec)** |  |  |  |  |  |  |  |  |  |  |  |  |  |  |  |  |  |  |  |  |  |  |  |  |  |  |  |  |  |  |  |  |  |  |
| 2 | 5.1911 | 311.466666666667 |  | **Plant1** | 298.22 | 298.29 | 297.17 | 297.89333 |  |  |  |  |  |  |  |  |  |  |  |  |  |  |  |  |  |  |  |  |  |  |  |  |  |  |  |  |  |  |  |  |  |  |
| 3 | 4.6537 | 279.22 |  | **Plant2** | 310.57 | 312.04 | 311.79 | 311.46667 |  |  |  |  |  |  |  |  |  |  |  |  |  |  |  |  |  |  |  |  |  |  |  |  |  |  |  |  |  |  |  |  |  |  |
| 4 | 4.7699 | 286.193333333333 |  | **Plant3** | 279.17 | 278.88 | 279.61 | 279.22000 |  |  |  |  |  |  |  |  |  |  |  |  |  |  |  |  |  |  |  |  |  |  |  |  |  |  |  |  |  |  |  |  |  |  |
| 5 | 4.6655 | 279.93 |  | **Plant4** | 286.91 | 285.18 | 286.49 | 286.19333 |  |  |  |  |  |  |  |  |  |  |  |  |  |  |  |  |  |  |  |  |  |  |  |  |  |  |  |  |  |  |  |  |  |  |
| 6 | 5.0586 | 303.516666666667 |  | **Plant5** | 279.76 | 278.75 | 281.28 | 279.93000 |  |  |  |  |  |  |  |  |  |  |  |  |  |  |  |  |  |  |  |  |  |  |  |  |  |  |  |  |  |  |  |  |  |  |
| **Average** | 4.8839 |  |  | **Plant6** | 303.09 | 303.36 | 304.1 | 303.51667 |  |  |  |  |  |  |  |  |  |  |  |  |  |  |  |  |  |  |  |  |  |  |  |  |  |  |  |  |  |  |  |  |  |  |
| **Sum** | 29.3036666666667 |  |  |  |  |  |  |  |  |  |  |  |  |  |  |  |  |  |  |  |  |  |  |  |  |  |  |  |  |  |  |  |  |  |  |  |  |  |  |  |  |  |
|  |  |  |  |  |  |  |  |  |  |  |  |  |  |  |  |  |  |  |  |  |  |  |  |  |  |  |  |  |  |  |  |  |  |  |  |  |  |  |  |  |  |  |
|  |  |  |  |  |  |  |  |  |  |  |  |  |  |  |  |  |  |  |  |  |  |  |  |  |  |  |  |  |  |  |  |  |  |  |  |  |  |  |  |  |  |  |
|  |  |  |  |  |  |  |  |  |  |  |  |  |  |  |  |  |  |  |  |  |  |  |  |  |  |  |  |  |  |  |  |  |  |  |  |  |  |  |  |  |  |  |
|  |  |  |  |  |  |  |  |  |  |  |  |  |  |  |  |  |  |  |  |  |  |  |  |  |  |  |  |  |  |  |  |  |  |  |  |  |  |  |  |  |  |  |
|  |  |  |  |  |  |  |  |  |  |  |  |  |  |  |  |  |  |  |  |  |  |  |  |  |  |  |  |  |  |  |  |  |  |  |  |  |  |  |  |  |  |  |
|  |  |  |  |  |  |  |  |  |  |  |  |  |  |  |  |  |  |  |  |  |  |  |  |  |  |  |  |  |  |  |  |  |  |  |  |  |  |  |  |  |  |  |
|  |  |  |  |  |  |  |  |  |  |  |  |  |  |  |  |  |  |  |  |  |  |  |  |  |  |  |  |  |  |  |  |  |  |  |  |  |  |  |  |  |  |  |
|  | **Distribution of the error** |  |  |  |  |  |  |  |  |  |  |  |  |  |  |  |  |  |  |  |  |  |  |  |  |  |  |  |  |  |  |  |  |  |  |  |  |  |  |  |  |  |
|  |  |  |  |  |  |  |  |  |  |  |  |  |  |  |  |  |  |  |  |  |  |  |  |  |  |  |  |  |  |  |  |  |  |  |  |  |  |  |  |  |  |  |
|  |  |  |  |  |  |  |  |  |  |  |  |  |  |  |  |  |  |  |  |  |  |  |  |  |  |  |  |  |  |  |  |  |  |  |  |  |  |  |  |  |  |  |
| **Range Text** |  | [0;2] | [2;4] | [4;6] | [6;8] | [8;10] | [10;12] | [12;14] | [14;16] | [16;18] | [18;20] | [20;22] | [22;24] | [24;26] | [26;28] | [28;30] | [30;32] | [32;34] | [34;36] | [36;38] | [38;40] | [40;42] | [42;44] | [44;46] | [46;48] | [48;50] | [50;52] | [52;54] | [54;56] | [56;58] | [58;60] | [60;62] | [62;64] | [64;66] | [66;68] | [68;70] | [70;72] | [72;74] | [74;76] | [76;78] | [78;80] |  |
| **Text Error** | 0 | 0.02 | 0.04 | 0.06 | 0.08 | 0.1 | 0.12 | 0.14 | 0.16 | 0.18 | 0.2 | 0.22 | 0.24 | 0.26 | 0.28 | 0.3 | 0.32 | 0.34 | 0.36 | 0.38 | 0.4 | 0.42 | 0.44 | 0.46 | 0.48 | 0.5 | 0.52 | 0.54 | 0.56 | 0.58 | 0.6 | 0.62 | 0.64 | 0.66 | 0.68 | 0.7 | 0.72 | 0.74 | 0.76 | 0.78 | 0.8 |  |
| **Error** | **0** | **0.02** | **0.04** | **0.06** | **0.08** | **0.1** | **0.12** | **0.14** | **0.16** | **0.18** | **0.2** | **0.22** | **0.24** | **0.26** | **0.28** | **0.3** | **0.32** | **0.34** | **0.36** | **0.38** | **0.4** | **0.42** | **0.44** | **0.46** | **0.48** | **0.5** | **0.52** | **0.54** | **0.56** | **0.58** | **0.6** | **0.62** | **0.64** | **0.66** | **0.68** | **0.7** | **0.72** | **0.74** | **0.76** | **0.78** | **0.8** | **SUM** |
| **Leaves Width** | 0 | 44 | 38 | 47 | 17 | 16 | 5 | 1 | 0 | 2 | 3 | 1 | 0 | 1 | 1 | 0 | 1 | 1 | 0 | 0 | 1 | 0 | 1 | 0 | 0 | 0 | 0 | 0 | 0 | 0 | 0 | 0 | 0 | 0 | 0 | 0 | 0 | 0 | 0 | 0 | 0 | **180** |
| **Leaves Length** | 0 | 32 | 30 | 17 | 18 | 20 | 17 | 14 | 6 | 9 | 3 | 4 | 3 | 2 | 1 | 1 | 0 | 1 | 0 | 1 | 0 | 0 | 0 | 0 | 0 | 0 | 0 | 0 | 0 | 0 | 0 | 0 | 0 | 0 | 0 | 0 | 0 | 1 | 0 | 0 | 0 | **180** |
| **Stem Height** | 0 | 6 | 4 | 2 | 5 | 1 | 0 | 0 | 0 | 3 | 0 | 0 | 0 | 0 | 1 | 0 | 1 | 0 | 0 | 0 | 0 | 0 | 0 | 0 | 1 | 0 | 0 | 0 | 0 | 0 | 0 | 0 | 0 | 0 | 0 | 0 | 0 | 0 | 0 | 0 | 0 | **24** |
|  |  |  |  |  |  |  |  |  |  |  |  |  |  |  |  |  |  |  |  |  |  |  |  |  |  |  |  |  |  |  |  |  |  |  |  |  |  |  |  |  |  | 384 |
|  |  |  |  |  |  |  |  |  |  |  |  |  |  |  |  |  |  |  |  |  |  |  |  |  |  |  |  |  |  |  |  |  |  |  |  |  |  |  |  |  |  |  |
| [ |  |  |  |  |  |  |  |  |  |  |  |  |  |  |  |  |  |  |  |  |  |  |  |  |  |  |  |  |  |  |  |  |  |  |  |  |  |  |  |  |  |  |
| ] |  |  |  |  |  |  |  |  |  |  |  |  |  |  |  |  |  |  |  |  |  |  |  |  |  |  |  |  |  |  |  |  |  |  |  |  |  |  |  |  |  |  |
| ; |  |  |  |  |  |  |  |  |  |  |  |  |  |  |  |  |  |  |  |  |  |  |  |  |  |  |  |  |  |  |  |  |  |  |  |  |  |  |  |  |  |  |
|  |  |  |  |  |  |  |  |  |  |  |  |  |  |  |  |  |  |  |  |  |  |  |  |  |  |  |  |  |  |  |  |  |  |  |  |  |  |  |  |  |  |  |
| **Trimmed statistics** |  |  |  |  |  |  |  |  |  |  |  |  |  |  |  |  |  |  |  |  |  |  |  |  |  |  |  |  |  |  |  |  |  |  |  |  |  |  |  |  |  |  |
|  |  |  |  |  |  |  |  |  |  |  |  |  |  |  |  |  |  |  |  |  |  |  |  |  |  |  |  |  |  |  |  |  |  |  |  |  |  |  |  |  |  |  |
|  |  |  |  |  |  |  |  |  |  |  |  |  |  |  |  |  |  |  |  |  |  |  |  |  |  |  |  |  |  |  |  |  |  |  |  |  |  |  |  |  |  |  |
|  |  |  |  |  |  |  |  |  |  |  |  |  |  |  |  |  |  |  |  |  |  |  |  |  |  |  |  |  |  |  |  |  |  |  |  |  |  |  |  |  |  |  |
|  |  |  |  |  |  |  |  |  |  |  |  |  |  |  |  |  |  |  |  |  |  |  |  |  |  |  |  |  |  |  |  |  |  |  |  |  |  |  |  |  |  |  |
|  |  |  |  |  |  |  |  |  |  |  |  |  |  |  |  |  |  |  |  |  |  |  |  |  |  |  |  |  |  |  |  |  |  |  |  |  |  |  |  |  |  |  |
| **Trimmed Width** |  |  | **Leaf Length** |  |  | **Main stem data** |  |  |  |  |  |  |  |  |  |  |  |  |  |  |  |  |  |  |  |  |  |  |  |  |  |  |  |  |  |  |  |  |  |  |  |  |
| 0.00056 |  |  | 0.00060 |  |  | 0.00111 |  |  |  |  |  |  |  |  |  |  |  |  |  |  |  |  |  |  |  |  |  |  |  |  |  |  |  |  |  |  |  |  |  |  |  |  |
| 0.00245 |  |  | 0.00118 |  |  | 0.00148 |  |  |  |  |  |  |  |  |  |  |  |  |  |  |  |  |  |  |  |  |  |  |  |  |  |  |  |  |  |  |  |  |  |  |  |  |
| 0.00257 |  |  | 0.00408 |  |  | 0.00183 |  |  |  |  |  |  |  |  |  |  |  |  |  |  |  |  |  |  |  |  |  |  |  |  |  |  |  |  |  |  |  |  |  |  |  |  |
| 0.00269 |  |  | 0.00532 |  |  | 0.00501 |  |  |  |  |  |  |  |  |  |  |  |  |  |  |  |  |  |  |  |  |  |  |  |  |  |  |  |  |  |  |  |  |  |  |  |  |
| 0.00344 |  |  | 0.00566 |  |  | 0.01395 |  |  |  |  |  |  |  |  |  |  |  |  |  |  |  |  |  |  |  |  |  |  |  |  |  |  |  |  |  |  |  |  |  |  |  |  |
| 0.00383 |  |  | 0.00613 |  |  | 0.01545 |  |  |  |  |  |  |  |  |  |  |  |  |  |  |  |  |  |  |  |  |  |  |  |  |  |  |  |  |  |  |  |  |  |  |  |  |
| 0.00383 |  |  | 0.00635 |  |  | 0.02284 |  |  |  |  |  |  |  |  |  |  |  |  |  |  |  |  |  |  |  |  |  |  |  |  |  |  |  |  |  |  |  |  |  |  |  |  |
| 0.00420 |  |  | 0.00683 |  |  | 0.02886 |  |  |  |  |  |  |  |  |  |  |  |  |  |  |  |  |  |  |  |  |  |  |  |  |  |  |  |  |  |  |  |  |  |  |  |  |
| 0.00450 |  |  | 0.00763 |  |  | 0.03080 |  |  |  |  |  |  |  |  |  |  |  |  |  |  |  |  |  |  |  |  |  |  |  |  |  |  |  |  |  |  |  |  |  |  |  |  |
| 0.00504 |  |  | 0.00791 |  |  | 0.03298 |  |  |  |  |  |  |  |  |  |  |  |  |  |  |  |  |  |  |  |  |  |  |  |  |  |  |  |  |  |  |  |  |  |  |  |  |
| 0.00704 |  |  | 0.00911 |  |  | 0.04586 |  |  |  |  |  |  |  |  |  |  |  |  |  |  |  |  |  |  |  |  |  |  |  |  |  |  |  |  |  |  |  |  |  |  |  |  |
| 0.00719 |  |  | 0.00994 |  |  | 0.05297 |  |  |  |  |  |  |  |  |  |  |  |  |  |  |  |  |  |  |  |  |  |  |  |  |  |  |  |  |  |  |  |  |  |  |  |  |
| 0.00762 |  |  | 0.01029 |  |  | 0.06212 |  |  |  |  |  |  |  |  |  |  |  |  |  |  |  |  |  |  |  |  |  |  |  |  |  |  |  |  |  |  |  |  |  |  |  |  |
| 0.00794 |  |  | 0.01046 |  |  | 0.06903 |  |  |  |  |  |  |  |  |  |  |  |  |  |  |  |  |  |  |  |  |  |  |  |  |  |  |  |  |  |  |  |  |  |  |  |  |
| 0.00797 |  |  | 0.01065 |  |  | 0.07160 |  |  |  |  |  |  |  |  |  |  |  |  |  |  |  |  |  |  |  |  |  |  |  |  |  |  |  |  |  |  |  |  |  |  |  |  |
| 0.00810 |  |  | 0.01080 |  |  | 0.07316 |  |  |  |  |  |  |  |  |  |  |  |  |  |  |  |  |  |  |  |  |  |  |  |  |  |  |  |  |  |  |  |  |  |  |  |  |
| 0.00822 |  |  | 0.01087 |  |  | 0.07929 |  |  |  |  |  |  |  |  |  |  |  |  |  |  |  |  |  |  |  |  |  |  |  |  |  |  |  |  |  |  |  |  |  |  |  |  |
| 0.00829 |  |  | 0.01151 |  |  | 0.08731 |  |  |  |  |  |  |  |  |  |  |  |  |  |  |  |  |  |  |  |  |  |  |  |  |  |  |  |  |  |  |  |  |  |  |  |  |
| 0.00882 |  |  | 0.01187 |  |  | 0.16256 |  |  |  |  |  |  |  |  |  |  |  |  |  |  |  |  |  |  |  |  |  |  |  |  |  |  |  |  |  |  |  |  |  |  |  |  |
| 0.00882 |  |  | 0.01202 |  |  | 0.16371 |  |  |  |  |  |  |  |  |  |  |  |  |  |  |  |  |  |  |  |  |  |  |  |  |  |  |  |  |  |  |  |  |  |  |  |  |
| 0.00924 |  |  | 0.01213 |  |  | 0.17029 |  |  |  |  |  |  |  |  |  |  |  |  |  |  |  |  |  |  |  |  |  |  |  |  |  |  |  |  |  |  |  |  |  |  |  |  |
| 0.01078 |  |  | 0.01249 |  |  | 0.26884 |  |  |  |  |  |  |  |  |  |  |  |  |  |  |  |  |  |  |  |  |  |  |  |  |  |  |  |  |  |  |  |  |  |  |  |  |
| 0.01148 |  |  | 0.01271 |  |  | 0.30848 |  |  |  |  |  |  |  |  |  |  |  |  |  |  |  |  |  |  |  |  |  |  |  |  |  |  |  |  |  |  |  |  |  |  |  |  |
| 0.01215 |  |  | 0.01382 |  |  | 0.47089 |  |  |  |  |  |  |  |  |  |  |  |  |  |  |  |  |  |  |  |  |  |  |  |  |  |  |  |  |  |  |  |  |  |  |  |  |
| 0.01316 |  |  | 0.01402 |  |  | **0.07292** | **Error** |  |  |  |  |  |  |  |  |  |  |  |  |  |  |  |  |  |  |  |  |  |  |  |  |  |  |  |  |  |  |  |  |  |  |  |
| 0.01323 |  |  | 0.01478 |  |  | **0.06879** | **STDEV** |  |  |  |  |  |  |  |  |  |  |  |  |  |  |  |  |  |  |  |  |  |  |  |  |  |  |  |  |  |  |  |  |  |  |  |
| 0.01336 |  |  | 0.01535 |  |  |  |  |  |  |  |  |  |  |  |  |  |  |  |  |  |  |  |  |  |  |  |  |  |  |  |  |  |  |  |  |  |  |  |  |  |  |  |
| 0.01344 |  |  | 0.01563 |  |  |  |  |  |  |  |  |  |  |  |  |  |  |  |  |  |  |  |  |  |  |  |  |  |  |  |  |  |  |  |  |  |  |  |  |  |  |  |
| 0.01380 |  |  | 0.01740 |  |  |  |  |  |  |  |  |  |  |  |  |  |  |  |  |  |  |  |  |  |  |  |  |  |  |  |  |  |  |  |  |  |  |  |  |  |  |  |
| 0.01436 |  |  | 0.01917 |  |  |  |  |  |  |  |  |  |  |  |  |  |  |  |  |  |  |  |  |  |  |  |  |  |  |  |  |  |  |  |  |  |  |  |  |  |  |  |
| 0.01437 |  |  | 0.01958 |  |  |  |  |  |  |  |  |  |  |  |  |  |  |  |  |  |  |  |  |  |  |  |  |  |  |  |  |  |  |  |  |  |  |  |  |  |  |  |
| 0.01453 |  |  | 0.01995 |  |  |  |  |  |  |  |  |  |  |  |  |  |  |  |  |  |  |  |  |  |  |  |  |  |  |  |  |  |  |  |  |  |  |  |  |  |  |  |
| 0.01496 |  |  | 0.02034 |  |  |  |  |  |  |  |  |  |  |  |  |  |  |  |  |  |  |  |  |  |  |  |  |  |  |  |  |  |  |  |  |  |  |  |  |  |  |  |
| 0.01548 |  |  | 0.02142 |  |  |  |  |  |  |  |  |  |  |  |  |  |  |  |  |  |  |  |  |  |  |  |  |  |  |  |  |  |  |  |  |  |  |  |  |  |  |  |
| 0.01617 |  |  | 0.02214 |  |  |  |  |  |  |  |  |  |  |  |  |  |  |  |  |  |  |  |  |  |  |  |  |  |  |  |  |  |  |  |  |  |  |  |  |  |  |  |
| 0.01643 |  |  | 0.02263 |  |  |  |  |  |  |  |  |  |  |  |  |  |  |  |  |  |  |  |  |  |  |  |  |  |  |  |  |  |  |  |  |  |  |  |  |  |  |  |
| 0.01734 |  |  | 0.02398 |  |  |  |  |  |  |  |  |  |  |  |  |  |  |  |  |  |  |  |  |  |  |  |  |  |  |  |  |  |  |  |  |  |  |  |  |  |  |  |
| 0.01740 |  |  | 0.02422 |  |  |  |  |  |  |  |  |  |  |  |  |  |  |  |  |  |  |  |  |  |  |  |  |  |  |  |  |  |  |  |  |  |  |  |  |  |  |  |
| 0.01742 |  |  | 0.02469 |  |  |  |  |  |  |  |  |  |  |  |  |  |  |  |  |  |  |  |  |  |  |  |  |  |  |  |  |  |  |  |  |  |  |  |  |  |  |  |
| 0.01749 |  |  | 0.02599 |  |  |  |  |  |  |  |  |  |  |  |  |  |  |  |  |  |  |  |  |  |  |  |  |  |  |  |  |  |  |  |  |  |  |  |  |  |  |  |
| 0.01758 |  |  | 0.02615 |  |  |  |  |  |  |  |  |  |  |  |  |  |  |  |  |  |  |  |  |  |  |  |  |  |  |  |  |  |  |  |  |  |  |  |  |  |  |  |
| 0.01834 |  |  | 0.02624 |  |  |  |  |  |  |  |  |  |  |  |  |  |  |  |  |  |  |  |  |  |  |  |  |  |  |  |  |  |  |  |  |  |  |  |  |  |  |  |
| 0.01948 |  |  | 0.02660 |  |  |  |  |  |  |  |  |  |  |  |  |  |  |  |  |  |  |  |  |  |  |  |  |  |  |  |  |  |  |  |  |  |  |  |  |  |  |  |
| 0.01977 |  |  | 0.02662 |  |  |  |  |  |  |  |  |  |  |  |  |  |  |  |  |  |  |  |  |  |  |  |  |  |  |  |  |  |  |  |  |  |  |  |  |  |  |  |
| 0.02057 |  |  | 0.02765 |  |  |  |  |  |  |  |  |  |  |  |  |  |  |  |  |  |  |  |  |  |  |  |  |  |  |  |  |  |  |  |  |  |  |  |  |  |  |  |
| 0.02059 |  |  | 0.02786 |  |  |  |  |  |  |  |  |  |  |  |  |  |  |  |  |  |  |  |  |  |  |  |  |  |  |  |  |  |  |  |  |  |  |  |  |  |  |  |
| 0.02073 |  |  | 0.02845 |  |  |  |  |  |  |  |  |  |  |  |  |  |  |  |  |  |  |  |  |  |  |  |  |  |  |  |  |  |  |  |  |  |  |  |  |  |  |  |
| 0.02095 |  |  | 0.02847 |  |  |  |  |  |  |  |  |  |  |  |  |  |  |  |  |  |  |  |  |  |  |  |  |  |  |  |  |  |  |  |  |  |  |  |  |  |  |  |
| 0.02207 |  |  | 0.02995 |  |  |  |  |  |  |  |  |  |  |  |  |  |  |  |  |  |  |  |  |  |  |  |  |  |  |  |  |  |  |  |  |  |  |  |  |  |  |  |
| 0.02243 |  |  | 0.03029 |  |  |  |  |  |  |  |  |  |  |  |  |  |  |  |  |  |  |  |  |  |  |  |  |  |  |  |  |  |  |  |  |  |  |  |  |  |  |  |
| 0.02294 |  |  | 0.03066 |  |  |  |  |  |  |  |  |  |  |  |  |  |  |  |  |  |  |  |  |  |  |  |  |  |  |  |  |  |  |  |  |  |  |  |  |  |  |  |
| 0.02306 |  |  | 0.03115 |  |  |  |  |  |  |  |  |  |  |  |  |  |  |  |  |  |  |  |  |  |  |  |  |  |  |  |  |  |  |  |  |  |  |  |  |  |  |  |
| 0.02345 |  |  | 0.03225 |  |  |  |  |  |  |  |  |  |  |  |  |  |  |  |  |  |  |  |  |  |  |  |  |  |  |  |  |  |  |  |  |  |  |  |  |  |  |  |
| 0.02466 |  |  | 0.03243 |  |  |  |  |  |  |  |  |  |  |  |  |  |  |  |  |  |  |  |  |  |  |  |  |  |  |  |  |  |  |  |  |  |  |  |  |  |  |  |
| 0.02596 |  |  | 0.03287 |  |  |  |  |  |  |  |  |  |  |  |  |  |  |  |  |  |  |  |  |  |  |  |  |  |  |  |  |  |  |  |  |  |  |  |  |  |  |  |
| 0.02598 |  |  | 0.03502 |  |  |  |  |  |  |  |  |  |  |  |  |  |  |  |  |  |  |  |  |  |  |  |  |  |  |  |  |  |  |  |  |  |  |  |  |  |  |  |
| 0.02717 |  |  | 0.03605 |  |  |  |  |  |  |  |  |  |  |  |  |  |  |  |  |  |  |  |  |  |  |  |  |  |  |  |  |  |  |  |  |  |  |  |  |  |  |  |
| 0.02817 |  |  | 0.03750 |  |  |  |  |  |  |  |  |  |  |  |  |  |  |  |  |  |  |  |  |  |  |  |  |  |  |  |  |  |  |  |  |  |  |  |  |  |  |  |
| 0.02847 |  |  | 0.03760 |  |  |  |  |  |  |  |  |  |  |  |  |  |  |  |  |  |  |  |  |  |  |  |  |  |  |  |  |  |  |  |  |  |  |  |  |  |  |  |
| 0.02851 |  |  | 0.03833 |  |  |  |  |  |  |  |  |  |  |  |  |  |  |  |  |  |  |  |  |  |  |  |  |  |  |  |  |  |  |  |  |  |  |  |  |  |  |  |
| 0.03073 |  |  | 0.03898 |  |  |  |  |  |  |  |  |  |  |  |  |  |  |  |  |  |  |  |  |  |  |  |  |  |  |  |  |  |  |  |  |  |  |  |  |  |  |  |
| 0.03102 |  |  | 0.03979 |  |  |  |  |  |  |  |  |  |  |  |  |  |  |  |  |  |  |  |  |  |  |  |  |  |  |  |  |  |  |  |  |  |  |  |  |  |  |  |
| 0.03152 |  |  | 0.04016 |  |  |  |  |  |  |  |  |  |  |  |  |  |  |  |  |  |  |  |  |  |  |  |  |  |  |  |  |  |  |  |  |  |  |  |  |  |  |  |
| 0.03204 |  |  | 0.04054 |  |  |  |  |  |  |  |  |  |  |  |  |  |  |  |  |  |  |  |  |  |  |  |  |  |  |  |  |  |  |  |  |  |  |  |  |  |  |  |
| 0.03214 |  |  | 0.04432 |  |  |  |  |  |  |  |  |  |  |  |  |  |  |  |  |  |  |  |  |  |  |  |  |  |  |  |  |  |  |  |  |  |  |  |  |  |  |  |
| 0.03287 |  |  | 0.04541 |  |  |  |  |  |  |  |  |  |  |  |  |  |  |  |  |  |  |  |  |  |  |  |  |  |  |  |  |  |  |  |  |  |  |  |  |  |  |  |
| 0.03303 |  |  | 0.04603 |  |  |  |  |  |  |  |  |  |  |  |  |  |  |  |  |  |  |  |  |  |  |  |  |  |  |  |  |  |  |  |  |  |  |  |  |  |  |  |
| 0.03325 |  |  | 0.04665 |  |  |  |  |  |  |  |  |  |  |  |  |  |  |  |  |  |  |  |  |  |  |  |  |  |  |  |  |  |  |  |  |  |  |  |  |  |  |  |
| 0.03419 |  |  | 0.04963 |  |  |  |  |  |  |  |  |  |  |  |  |  |  |  |  |  |  |  |  |  |  |  |  |  |  |  |  |  |  |  |  |  |  |  |  |  |  |  |
| 0.03421 |  |  | 0.05155 |  |  |  |  |  |  |  |  |  |  |  |  |  |  |  |  |  |  |  |  |  |  |  |  |  |  |  |  |  |  |  |  |  |  |  |  |  |  |  |
| 0.03481 |  |  | 0.05171 |  |  |  |  |  |  |  |  |  |  |  |  |  |  |  |  |  |  |  |  |  |  |  |  |  |  |  |  |  |  |  |  |  |  |  |  |  |  |  |
| 0.03499 |  |  | 0.05437 |  |  |  |  |  |  |  |  |  |  |  |  |  |  |  |  |  |  |  |  |  |  |  |  |  |  |  |  |  |  |  |  |  |  |  |  |  |  |  |
| 0.03619 |  |  | 0.05456 |  |  |  |  |  |  |  |  |  |  |  |  |  |  |  |  |  |  |  |  |  |  |  |  |  |  |  |  |  |  |  |  |  |  |  |  |  |  |  |
| 0.03637 |  |  | 0.05475 |  |  |  |  |  |  |  |  |  |  |  |  |  |  |  |  |  |  |  |  |  |  |  |  |  |  |  |  |  |  |  |  |  |  |  |  |  |  |  |
| 0.03666 |  |  | 0.05511 |  |  |  |  |  |  |  |  |  |  |  |  |  |  |  |  |  |  |  |  |  |  |  |  |  |  |  |  |  |  |  |  |  |  |  |  |  |  |  |
| 0.03670 |  |  | 0.05602 |  |  |  |  |  |  |  |  |  |  |  |  |  |  |  |  |  |  |  |  |  |  |  |  |  |  |  |  |  |  |  |  |  |  |  |  |  |  |  |
| 0.03696 |  |  | 0.05665 |  |  |  |  |  |  |  |  |  |  |  |  |  |  |  |  |  |  |  |  |  |  |  |  |  |  |  |  |  |  |  |  |  |  |  |  |  |  |  |
| 0.03696 |  |  | 0.05703 |  |  |  |  |  |  |  |  |  |  |  |  |  |  |  |  |  |  |  |  |  |  |  |  |  |  |  |  |  |  |  |  |  |  |  |  |  |  |  |
| 0.03796 |  |  | 0.05769 |  |  |  |  |  |  |  |  |  |  |  |  |  |  |  |  |  |  |  |  |  |  |  |  |  |  |  |  |  |  |  |  |  |  |  |  |  |  |  |
| 0.03874 |  |  | 0.06023 |  |  |  |  |  |  |  |  |  |  |  |  |  |  |  |  |  |  |  |  |  |  |  |  |  |  |  |  |  |  |  |  |  |  |  |  |  |  |  |
| 0.03963 |  |  | 0.06166 |  |  |  |  |  |  |  |  |  |  |  |  |  |  |  |  |  |  |  |  |  |  |  |  |  |  |  |  |  |  |  |  |  |  |  |  |  |  |  |
| 0.03995 |  |  | 0.06253 |  |  |  |  |  |  |  |  |  |  |  |  |  |  |  |  |  |  |  |  |  |  |  |  |  |  |  |  |  |  |  |  |  |  |  |  |  |  |  |
| 0.04041 |  |  | 0.06273 |  |  |  |  |  |  |  |  |  |  |  |  |  |  |  |  |  |  |  |  |  |  |  |  |  |  |  |  |  |  |  |  |  |  |  |  |  |  |  |
| 0.04077 |  |  | 0.06417 |  |  |  |  |  |  |  |  |  |  |  |  |  |  |  |  |  |  |  |  |  |  |  |  |  |  |  |  |  |  |  |  |  |  |  |  |  |  |  |
| 0.04102 |  |  | 0.06530 |  |  |  |  |  |  |  |  |  |  |  |  |  |  |  |  |  |  |  |  |  |  |  |  |  |  |  |  |  |  |  |  |  |  |  |  |  |  |  |
| 0.04145 |  |  | 0.06549 |  |  |  |  |  |  |  |  |  |  |  |  |  |  |  |  |  |  |  |  |  |  |  |  |  |  |  |  |  |  |  |  |  |  |  |  |  |  |  |
| 0.04151 |  |  | 0.06570 |  |  |  |  |  |  |  |  |  |  |  |  |  |  |  |  |  |  |  |  |  |  |  |  |  |  |  |  |  |  |  |  |  |  |  |  |  |  |  |
| 0.04231 |  |  | 0.06608 |  |  |  |  |  |  |  |  |  |  |  |  |  |  |  |  |  |  |  |  |  |  |  |  |  |  |  |  |  |  |  |  |  |  |  |  |  |  |  |
| 0.04335 |  |  | 0.06707 |  |  |  |  |  |  |  |  |  |  |  |  |  |  |  |  |  |  |  |  |  |  |  |  |  |  |  |  |  |  |  |  |  |  |  |  |  |  |  |
| 0.04337 |  |  | 0.06974 |  |  |  |  |  |  |  |  |  |  |  |  |  |  |  |  |  |  |  |  |  |  |  |  |  |  |  |  |  |  |  |  |  |  |  |  |  |  |  |
| 0.04365 |  |  | 0.07432 |  |  |  |  |  |  |  |  |  |  |  |  |  |  |  |  |  |  |  |  |  |  |  |  |  |  |  |  |  |  |  |  |  |  |  |  |  |  |  |
| 0.04385 |  |  | 0.07746 |  |  |  |  |  |  |  |  |  |  |  |  |  |  |  |  |  |  |  |  |  |  |  |  |  |  |  |  |  |  |  |  |  |  |  |  |  |  |  |
| 0.04471 |  |  | 0.07807 |  |  |  |  |  |  |  |  |  |  |  |  |  |  |  |  |  |  |  |  |  |  |  |  |  |  |  |  |  |  |  |  |  |  |  |  |  |  |  |
| 0.04493 |  |  | 0.07812 |  |  |  |  |  |  |  |  |  |  |  |  |  |  |  |  |  |  |  |  |  |  |  |  |  |  |  |  |  |  |  |  |  |  |  |  |  |  |  |
| 0.04544 |  |  | 0.07827 |  |  |  |  |  |  |  |  |  |  |  |  |  |  |  |  |  |  |  |  |  |  |  |  |  |  |  |  |  |  |  |  |  |  |  |  |  |  |  |
| 0.04628 |  |  | 0.07943 |  |  |  |  |  |  |  |  |  |  |  |  |  |  |  |  |  |  |  |  |  |  |  |  |  |  |  |  |  |  |  |  |  |  |  |  |  |  |  |
| 0.04643 |  |  | 0.07975 |  |  |  |  |  |  |  |  |  |  |  |  |  |  |  |  |  |  |  |  |  |  |  |  |  |  |  |  |  |  |  |  |  |  |  |  |  |  |  |
| 0.04673 |  |  | 0.08169 |  |  |  |  |  |  |  |  |  |  |  |  |  |  |  |  |  |  |  |  |  |  |  |  |  |  |  |  |  |  |  |  |  |  |  |  |  |  |  |
| 0.04782 |  |  | 0.08221 |  |  |  |  |  |  |  |  |  |  |  |  |  |  |  |  |  |  |  |  |  |  |  |  |  |  |  |  |  |  |  |  |  |  |  |  |  |  |  |
| 0.04824 |  |  | 0.08223 |  |  |  |  |  |  |  |  |  |  |  |  |  |  |  |  |  |  |  |  |  |  |  |  |  |  |  |  |  |  |  |  |  |  |  |  |  |  |  |
| 0.04826 |  |  | 0.08223 |  |  |  |  |  |  |  |  |  |  |  |  |  |  |  |  |  |  |  |  |  |  |  |  |  |  |  |  |  |  |  |  |  |  |  |  |  |  |  |
| 0.04897 |  |  | 0.08244 |  |  |  |  |  |  |  |  |  |  |  |  |  |  |  |  |  |  |  |  |  |  |  |  |  |  |  |  |  |  |  |  |  |  |  |  |  |  |  |
| 0.04933 |  |  | 0.08334 |  |  |  |  |  |  |  |  |  |  |  |  |  |  |  |  |  |  |  |  |  |  |  |  |  |  |  |  |  |  |  |  |  |  |  |  |  |  |  |
| 0.04971 |  |  | 0.08490 |  |  |  |  |  |  |  |  |  |  |  |  |  |  |  |  |  |  |  |  |  |  |  |  |  |  |  |  |  |  |  |  |  |  |  |  |  |  |  |
| 0.05042 |  |  | 0.08791 |  |  |  |  |  |  |  |  |  |  |  |  |  |  |  |  |  |  |  |  |  |  |  |  |  |  |  |  |  |  |  |  |  |  |  |  |  |  |  |
| 0.05044 |  |  | 0.08813 |  |  |  |  |  |  |  |  |  |  |  |  |  |  |  |  |  |  |  |  |  |  |  |  |  |  |  |  |  |  |  |  |  |  |  |  |  |  |  |
| 0.05046 |  |  | 0.09052 |  |  |  |  |  |  |  |  |  |  |  |  |  |  |  |  |  |  |  |  |  |  |  |  |  |  |  |  |  |  |  |  |  |  |  |  |  |  |  |
| 0.05048 |  |  | 0.09204 |  |  |  |  |  |  |  |  |  |  |  |  |  |  |  |  |  |  |  |  |  |  |  |  |  |  |  |  |  |  |  |  |  |  |  |  |  |  |  |
| 0.05056 |  |  | 0.09237 |  |  |  |  |  |  |  |  |  |  |  |  |  |  |  |  |  |  |  |  |  |  |  |  |  |  |  |  |  |  |  |  |  |  |  |  |  |  |  |
| 0.05120 |  |  | 0.09270 |  |  |  |  |  |  |  |  |  |  |  |  |  |  |  |  |  |  |  |  |  |  |  |  |  |  |  |  |  |  |  |  |  |  |  |  |  |  |  |
| 0.05155 |  |  | 0.09297 |  |  |  |  |  |  |  |  |  |  |  |  |  |  |  |  |  |  |  |  |  |  |  |  |  |  |  |  |  |  |  |  |  |  |  |  |  |  |  |
| 0.05159 |  |  | 0.09352 |  |  |  |  |  |  |  |  |  |  |  |  |  |  |  |  |  |  |  |  |  |  |  |  |  |  |  |  |  |  |  |  |  |  |  |  |  |  |  |
| 0.05168 |  |  | 0.09369 |  |  |  |  |  |  |  |  |  |  |  |  |  |  |  |  |  |  |  |  |  |  |  |  |  |  |  |  |  |  |  |  |  |  |  |  |  |  |  |
| 0.05289 |  |  | 0.09720 |  |  |  |  |  |  |  |  |  |  |  |  |  |  |  |  |  |  |  |  |  |  |  |  |  |  |  |  |  |  |  |  |  |  |  |  |  |  |  |
| 0.05404 |  |  | 0.09778 |  |  |  |  |  |  |  |  |  |  |  |  |  |  |  |  |  |  |  |  |  |  |  |  |  |  |  |  |  |  |  |  |  |  |  |  |  |  |  |
| 0.05405 |  |  | 0.09846 |  |  |  |  |  |  |  |  |  |  |  |  |  |  |  |  |  |  |  |  |  |  |  |  |  |  |  |  |  |  |  |  |  |  |  |  |  |  |  |
| 0.05418 |  |  | 0.09902 |  |  |  |  |  |  |  |  |  |  |  |  |  |  |  |  |  |  |  |  |  |  |  |  |  |  |  |  |  |  |  |  |  |  |  |  |  |  |  |
| 0.05494 |  |  | 0.10078 |  |  |  |  |  |  |  |  |  |  |  |  |  |  |  |  |  |  |  |  |  |  |  |  |  |  |  |  |  |  |  |  |  |  |  |  |  |  |  |
| 0.05530 |  |  | 0.10242 |  |  |  |  |  |  |  |  |  |  |  |  |  |  |  |  |  |  |  |  |  |  |  |  |  |  |  |  |  |  |  |  |  |  |  |  |  |  |  |
| 0.05544 |  |  | 0.10311 |  |  |  |  |  |  |  |  |  |  |  |  |  |  |  |  |  |  |  |  |  |  |  |  |  |  |  |  |  |  |  |  |  |  |  |  |  |  |  |
| 0.05579 |  |  | 0.10329 |  |  |  |  |  |  |  |  |  |  |  |  |  |  |  |  |  |  |  |  |  |  |  |  |  |  |  |  |  |  |  |  |  |  |  |  |  |  |  |
| 0.05609 |  |  | 0.10342 |  |  |  |  |  |  |  |  |  |  |  |  |  |  |  |  |  |  |  |  |  |  |  |  |  |  |  |  |  |  |  |  |  |  |  |  |  |  |  |
| 0.05642 |  |  | 0.10674 |  |  |  |  |  |  |  |  |  |  |  |  |  |  |  |  |  |  |  |  |  |  |  |  |  |  |  |  |  |  |  |  |  |  |  |  |  |  |  |
| 0.05812 |  |  | 0.10739 |  |  |  |  |  |  |  |  |  |  |  |  |  |  |  |  |  |  |  |  |  |  |  |  |  |  |  |  |  |  |  |  |  |  |  |  |  |  |  |
| 0.05856 |  |  | 0.10811 |  |  |  |  |  |  |  |  |  |  |  |  |  |  |  |  |  |  |  |  |  |  |  |  |  |  |  |  |  |  |  |  |  |  |  |  |  |  |  |
| 0.05876 |  |  | 0.11032 |  |  |  |  |  |  |  |  |  |  |  |  |  |  |  |  |  |  |  |  |  |  |  |  |  |  |  |  |  |  |  |  |  |  |  |  |  |  |  |
| 0.05920 |  |  | 0.11202 |  |  |  |  |  |  |  |  |  |  |  |  |  |  |  |  |  |  |  |  |  |  |  |  |  |  |  |  |  |  |  |  |  |  |  |  |  |  |  |
| 0.05976 |  |  | 0.11442 |  |  |  |  |  |  |  |  |  |  |  |  |  |  |  |  |  |  |  |  |  |  |  |  |  |  |  |  |  |  |  |  |  |  |  |  |  |  |  |
| 0.05977 |  |  | 0.11521 |  |  |  |  |  |  |  |  |  |  |  |  |  |  |  |  |  |  |  |  |  |  |  |  |  |  |  |  |  |  |  |  |  |  |  |  |  |  |  |
| 0.06055 |  |  | 0.11573 |  |  |  |  |  |  |  |  |  |  |  |  |  |  |  |  |  |  |  |  |  |  |  |  |  |  |  |  |  |  |  |  |  |  |  |  |  |  |  |
| 0.06251 |  |  | 0.11628 |  |  |  |  |  |  |  |  |  |  |  |  |  |  |  |  |  |  |  |  |  |  |  |  |  |  |  |  |  |  |  |  |  |  |  |  |  |  |  |
| 0.06390 |  |  | 0.11785 |  |  |  |  |  |  |  |  |  |  |  |  |  |  |  |  |  |  |  |  |  |  |  |  |  |  |  |  |  |  |  |  |  |  |  |  |  |  |  |
| 0.06467 |  |  | 0.11847 |  |  |  |  |  |  |  |  |  |  |  |  |  |  |  |  |  |  |  |  |  |  |  |  |  |  |  |  |  |  |  |  |  |  |  |  |  |  |  |
| 0.06593 |  |  | 0.11877 |  |  |  |  |  |  |  |  |  |  |  |  |  |  |  |  |  |  |  |  |  |  |  |  |  |  |  |  |  |  |  |  |  |  |  |  |  |  |  |
| 0.06631 |  |  | 0.12055 |  |  |  |  |  |  |  |  |  |  |  |  |  |  |  |  |  |  |  |  |  |  |  |  |  |  |  |  |  |  |  |  |  |  |  |  |  |  |  |
| 0.06799 |  |  | 0.12241 |  |  |  |  |  |  |  |  |  |  |  |  |  |  |  |  |  |  |  |  |  |  |  |  |  |  |  |  |  |  |  |  |  |  |  |  |  |  |  |
| 0.06917 |  |  | 0.12621 |  |  |  |  |  |  |  |  |  |  |  |  |  |  |  |  |  |  |  |  |  |  |  |  |  |  |  |  |  |  |  |  |  |  |  |  |  |  |  |
| 0.07130 |  |  | 0.12664 |  |  |  |  |  |  |  |  |  |  |  |  |  |  |  |  |  |  |  |  |  |  |  |  |  |  |  |  |  |  |  |  |  |  |  |  |  |  |  |
| 0.07172 |  |  | 0.12669 |  |  |  |  |  |  |  |  |  |  |  |  |  |  |  |  |  |  |  |  |  |  |  |  |  |  |  |  |  |  |  |  |  |  |  |  |  |  |  |
| 0.07232 |  |  | 0.12748 |  |  |  |  |  |  |  |  |  |  |  |  |  |  |  |  |  |  |  |  |  |  |  |  |  |  |  |  |  |  |  |  |  |  |  |  |  |  |  |
| 0.07320 |  |  | 0.12832 |  |  |  |  |  |  |  |  |  |  |  |  |  |  |  |  |  |  |  |  |  |  |  |  |  |  |  |  |  |  |  |  |  |  |  |  |  |  |  |
| 0.07489 |  |  | 0.13027 |  |  |  |  |  |  |  |  |  |  |  |  |  |  |  |  |  |  |  |  |  |  |  |  |  |  |  |  |  |  |  |  |  |  |  |  |  |  |  |
| 0.07490 |  |  | 0.13205 |  |  |  |  |  |  |  |  |  |  |  |  |  |  |  |  |  |  |  |  |  |  |  |  |  |  |  |  |  |  |  |  |  |  |  |  |  |  |  |
| 0.07844 |  |  | 0.13373 |  |  |  |  |  |  |  |  |  |  |  |  |  |  |  |  |  |  |  |  |  |  |  |  |  |  |  |  |  |  |  |  |  |  |  |  |  |  |  |
| 0.07859 |  |  | 0.13555 |  |  |  |  |  |  |  |  |  |  |  |  |  |  |  |  |  |  |  |  |  |  |  |  |  |  |  |  |  |  |  |  |  |  |  |  |  |  |  |
| 0.07982 |  |  | 0.13685 |  |  |  |  |  |  |  |  |  |  |  |  |  |  |  |  |  |  |  |  |  |  |  |  |  |  |  |  |  |  |  |  |  |  |  |  |  |  |  |
| 0.08042 |  |  | 0.13686 |  |  |  |  |  |  |  |  |  |  |  |  |  |  |  |  |  |  |  |  |  |  |  |  |  |  |  |  |  |  |  |  |  |  |  |  |  |  |  |
| 0.08160 |  |  | 0.13981 |  |  |  |  |  |  |  |  |  |  |  |  |  |  |  |  |  |  |  |  |  |  |  |  |  |  |  |  |  |  |  |  |  |  |  |  |  |  |  |
| 0.08289 |  |  | 0.14541 |  |  |  |  |  |  |  |  |  |  |  |  |  |  |  |  |  |  |  |  |  |  |  |  |  |  |  |  |  |  |  |  |  |  |  |  |  |  |  |
| 0.08312 |  |  | 0.14574 |  |  |  |  |  |  |  |  |  |  |  |  |  |  |  |  |  |  |  |  |  |  |  |  |  |  |  |  |  |  |  |  |  |  |  |  |  |  |  |
| 0.08460 |  |  | 0.14845 |  |  |  |  |  |  |  |  |  |  |  |  |  |  |  |  |  |  |  |  |  |  |  |  |  |  |  |  |  |  |  |  |  |  |  |  |  |  |  |
| 0.08634 |  |  | 0.15299 |  |  |  |  |  |  |  |  |  |  |  |  |  |  |  |  |  |  |  |  |  |  |  |  |  |  |  |  |  |  |  |  |  |  |  |  |  |  |  |
| 0.08643 |  |  | 0.15421 |  |  |  |  |  |  |  |  |  |  |  |  |  |  |  |  |  |  |  |  |  |  |  |  |  |  |  |  |  |  |  |  |  |  |  |  |  |  |  |
| 0.08689 |  |  | 0.15766 |  |  |  |  |  |  |  |  |  |  |  |  |  |  |  |  |  |  |  |  |  |  |  |  |  |  |  |  |  |  |  |  |  |  |  |  |  |  |  |
| 0.08691 |  |  | 0.16105 |  |  |  |  |  |  |  |  |  |  |  |  |  |  |  |  |  |  |  |  |  |  |  |  |  |  |  |  |  |  |  |  |  |  |  |  |  |  |  |
| 0.08790 |  |  | 0.16134 |  |  |  |  |  |  |  |  |  |  |  |  |  |  |  |  |  |  |  |  |  |  |  |  |  |  |  |  |  |  |  |  |  |  |  |  |  |  |  |
| 0.09012 |  |  | 0.16190 |  |  |  |  |  |  |  |  |  |  |  |  |  |  |  |  |  |  |  |  |  |  |  |  |  |  |  |  |  |  |  |  |  |  |  |  |  |  |  |
| 0.09036 |  |  | 0.16359 |  |  |  |  |  |  |  |  |  |  |  |  |  |  |  |  |  |  |  |  |  |  |  |  |  |  |  |  |  |  |  |  |  |  |  |  |  |  |  |
| 0.09229 |  |  | 0.16880 |  |  |  |  |  |  |  |  |  |  |  |  |  |  |  |  |  |  |  |  |  |  |  |  |  |  |  |  |  |  |  |  |  |  |  |  |  |  |  |
| 0.09489 |  |  | 0.16945 |  |  |  |  |  |  |  |  |  |  |  |  |  |  |  |  |  |  |  |  |  |  |  |  |  |  |  |  |  |  |  |  |  |  |  |  |  |  |  |
| 0.09536 |  |  | 0.17343 |  |  |  |  |  |  |  |  |  |  |  |  |  |  |  |  |  |  |  |  |  |  |  |  |  |  |  |  |  |  |  |  |  |  |  |  |  |  |  |
| 0.09633 |  |  | 0.17388 |  |  |  |  |  |  |  |  |  |  |  |  |  |  |  |  |  |  |  |  |  |  |  |  |  |  |  |  |  |  |  |  |  |  |  |  |  |  |  |
| 0.10603 |  |  | 0.17734 |  |  |  |  |  |  |  |  |  |  |  |  |  |  |  |  |  |  |  |  |  |  |  |  |  |  |  |  |  |  |  |  |  |  |  |  |  |  |  |
| 0.10679 |  |  | 0.18086 |  |  |  |  |  |  |  |  |  |  |  |  |  |  |  |  |  |  |  |  |  |  |  |  |  |  |  |  |  |  |  |  |  |  |  |  |  |  |  |
| 0.10729 |  |  | 0.19004 |  |  |  |  |  |  |  |  |  |  |  |  |  |  |  |  |  |  |  |  |  |  |  |  |  |  |  |  |  |  |  |  |  |  |  |  |  |  |  |
| 0.10927 |  |  | 0.19867 |  |  |  |  |  |  |  |  |  |  |  |  |  |  |  |  |  |  |  |  |  |  |  |  |  |  |  |  |  |  |  |  |  |  |  |  |  |  |  |
| 0.11426 |  |  | 0.20028 |  |  |  |  |  |  |  |  |  |  |  |  |  |  |  |  |  |  |  |  |  |  |  |  |  |  |  |  |  |  |  |  |  |  |  |  |  |  |  |
| 0.13327 |  |  | 0.20187 |  |  |  |  |  |  |  |  |  |  |  |  |  |  |  |  |  |  |  |  |  |  |  |  |  |  |  |  |  |  |  |  |  |  |  |  |  |  |  |
| 0.16388 |  |  | 0.20746 |  |  |  |  |  |  |  |  |  |  |  |  |  |  |  |  |  |  |  |  |  |  |  |  |  |  |  |  |  |  |  |  |  |  |  |  |  |  |  |
| 0.16694 |  |  | 0.21220 |  |  |  |  |  |  |  |  |  |  |  |  |  |  |  |  |  |  |  |  |  |  |  |  |  |  |  |  |  |  |  |  |  |  |  |  |  |  |  |
| 0.18086 |  |  | 0.22197 |  |  |  |  |  |  |  |  |  |  |  |  |  |  |  |  |  |  |  |  |  |  |  |  |  |  |  |  |  |  |  |  |  |  |  |  |  |  |  |
| 0.19049 |  |  | 0.22526 |  |  |  |  |  |  |  |  |  |  |  |  |  |  |  |  |  |  |  |  |  |  |  |  |  |  |  |  |  |  |  |  |  |  |  |  |  |  |  |
| 0.19099 |  |  | 0.23561 |  |  |  |  |  |  |  |  |  |  |  |  |  |  |  |  |  |  |  |  |  |  |  |  |  |  |  |  |  |  |  |  |  |  |  |  |  |  |  |
| 0.21443 |  |  | 0.24602 |  |  |  |  |  |  |  |  |  |  |  |  |  |  |  |  |  |  |  |  |  |  |  |  |  |  |  |  |  |  |  |  |  |  |  |  |  |  |  |
| 0.25688 |  |  | 0.24773 |  |  |  |  |  |  |  |  |  |  |  |  |  |  |  |  |  |  |  |  |  |  |  |  |  |  |  |  |  |  |  |  |  |  |  |  |  |  |  |
| 0.27036 |  |  | 0.26640 |  |  |  |  |  |  |  |  |  |  |  |  |  |  |  |  |  |  |  |  |  |  |  |  |  |  |  |  |  |  |  |  |  |  |  |  |  |  |  |
| 0.31382 |  |  | 0.29534 |  |  |  |  |  |  |  |  |  |  |  |  |  |  |  |  |  |  |  |  |  |  |  |  |  |  |  |  |  |  |  |  |  |  |  |  |  |  |  |
| 0.32017 |  |  | 0.33321 |  |  |  |  |  |  |  |  |  |  |  |  |  |  |  |  |  |  |  |  |  |  |  |  |  |  |  |  |  |  |  |  |  |  |  |  |  |  |  |
| 0.39141 |  |  | 0.36078 |  |  |  |  |  |  |  |  |  |  |  |  |  |  |  |  |  |  |  |  |  |  |  |  |  |  |  |  |  |  |  |  |  |  |  |  |  |  |  |
| 0.42521 |  |  | 0.72254 |  |  |  |  |  |  |  |  |  |  |  |  |  |  |  |  |  |  |  |  |  |  |  |  |  |  |  |  |  |  |  |  |  |  |  |  |  |  |  |
| **0.04781** | **Error** |  | **0.07919** | **Error** |  |  |  |  |  |  |  |  |  |  |  |  |  |  |  |  |  |  |  |  |  |  |  |  |  |  |  |  |  |  |  |  |  |  |  |  |  |  |
| **0.03199** | **STDEV** |  | **0.05415** | **STDEV** |  |  |  |  |  |  |  |  |  |  |  |  |  |  |  |  |  |  |  |  |  |  |  |  |  |  |  |  |  |  |  |  |  |  |  |  |  |  |
|  |  |  |  |  |  |  |  |  |  |  |  |  |  |  |  |  |  |  |  |  |  |  |  |  |  |  |  |  |  |  |  |  |  |  |  |  |  |  |  |  |  |  |
|  |  |  |  |  |  |  |  |  |  |  |  |  |  |  |  |  |  |  |  |  |  |  |  |  |  |  |  |  |  |  |  |  |  |  |  |  |  |  |  |  |  |  |
|  |  |  |  |  |  |  |  |  |  |  |  |  |  |  |  |  |  |  |  |  |  |  |  |  |  |  |  |  |  |  |  |  |  |  |  |  |  |  |  |  |  |  |
|  |  |  |  |  |  |  |  |  |  |  |  |  |  |  |  |  |  |  |  |  |  |  |  |  |  |  |  |  |  |  |  |  |  |  |  |  |  |  |  |  |  |  |
|  |  |  |  |  |  |  |  |  |  |  |  |  |  |  |  |  |  |  |  |  |  |  |  |  |  |  |  |  |  |  |  |  |  |  |  |  |  |  |  |  |  |  |
| **Study case** |  |  |  |  |  |  |  |  |  |  |  |  |  |  |  |  |  |  |  |  |  |  |  |  |  |  |  |  |  |  |  |  |  |  |  |  |  |  |  |  |  |  |
|  |  |  |  |  |  |  |  |  |  |  |  |  |  |  |  |  |  |  |  |  |  |  |  |  |  |  |  |  |  |  |  |  |  |  |  |  |  |  |  |  |  |  |
|  |  |  |  |  |  |  |  |  |  |  |  |  |  |  |  |  |  |  |  |  |  |  |  |  |  |  |  |  |  |  |  |  |  |  |  |  |  |  |  |  |  |  |
| **Number of Leaves** | **Plant1** | **Plant2** | **Plant3** | **Plant4** | **Plant5** | **Plant6** |  |  |  |  |  |  |  |  |  |  |  |  |  |  |  |  |  |  |  |  |  |  |  |  |  |  |  |  |  |  |  |  |  |  |  |  |
| **T0** | 7 | 9 | 6 | 5 | 5 | 6 |  |  |  |  |  |  |  |  |  |  |  |  |  |  |  |  |  |  |  |  |  |  |  |  |  |  |  |  |  |  |  |  |  |  |  |  |
| **T1** | 8 | 14 | 7 | 7 | 6 | 7 |  |  |  |  |  |  |  |  |  |  |  |  |  |  |  |  |  |  |  |  |  |  |  |  |  |  |  |  |  |  |  |  |  |  |  |  |
| **T2** | 10 | 14 | 8 | 7 | 8 | 9 |  |  |  |  |  |  |  |  |  |  |  |  |  |  |  |  |  |  |  |  |  |  |  |  |  |  |  |  |  |  |  |  |  |  |  |  |
| **T3** | 12 | 13 | 11 | 9 | 9 | 10 |  |  |  |  |  |  |  |  |  |  |  |  |  |  |  |  |  |  |  |  |  |  |  |  |  |  |  |  |  |  |  |  |  |  |  |  |
|  |  |  |  |  |  |  |  |  |  |  |  |  |  |  |  |  |  |  |  |  |  |  |  |  |  |  |  |  |  |  |  |  |  |  |  |  |  |  |  |  |  |  |
|  |  |  |  |  |  |  |  |  |  |  |  |  |  |  |  |  |  |  |  |  |  |  |  |  |  |  |  |  |  |  |  |  |  |  |  |  |  |  |  |  |  |  |
|  |  |  |  |  |  |  |  |  |  |  |  |  |  |  |  |  |  |  |  |  |  |  |  |  |  |  |  |  |  |  |  |  |  |  |  |  |  |  |  |  |  |  |
| **Main stem height** | **Plant1** | **Plant2** | **Plant3** | **Plant4** | **Plant5** | **Plant6** |  |  |  |  |  |  |  |  |  |  |  |  |  |  |  |  |  |  |  |  |  |  |  |  |  |  |  |  |  |  |  |  |  |  |  |  |
| **T0** | 142.775 | 182.391 | 130.848 | 108.225 | 88.355 | 121.779 |  |  |  |  |  |  |  |  |  |  |  |  |  |  |  |  |  |  |  |  |  |  |  |  |  |  |  |  |  |  |  |  |  |  |  |  |
| **T1** | 167.752 | 228.391 | 139.299 | 132.766 | 106.465 | 152.180 |  |  |  |  |  |  |  |  |  |  |  |  |  |  |  |  |  |  |  |  |  |  |  |  |  |  |  |  |  |  |  |  |  |  |  |  |
| **T2** | 222.978 | 232.538 | 169.609 | 166.184 | 135.922 | 188.568 |  |  |  |  |  |  |  |  |  |  |  |  |  |  |  |  |  |  |  |  |  |  |  |  |  |  |  |  |  |  |  |  |  |  |  |  |
| **T3** | 259.328 | 257.216 | 193.353 | 189.535 | 152.929 | 231.136 |  |  |  |  |  |  |  |  |  |  |  |  |  |  |  |  |  |  |  |  |  |  |  |  |  |  |  |  |  |  |  |  |  |  |  |  |
|  |  |  |  |  |  |  |  |  |  |  |  |  |  |  |  |  |  |  |  |  |  |  |  |  |  |  |  |  |  |  |  |  |  |  |  |  |  |  |  |  |  |  |
|  |  |  |  |  |  |  |  |  |  |  |  |  |  |  |  |  |  |  |  |  |  |  |  |  |  |  |  |  |  |  |  |  |  |  |  |  |  |  |  |  |  |  |
|  |  |  |  |  |  |  |  |  |  |  |  |  |  |  |  |  |  |  |  |  |  |  |  |  |  |  |  |  |  |  |  |  |  |  |  |  |  |  |  |  |  |  |
| **Average Area** | **Plant1** | **Plant2** | **Plant3** | **Plant4** | **Plant5** | **Plant6** |  |  |  |  |  |  |  |  |  |  |  |  |  |  |  |  |  |  |  |  |  |  |  |  |  |  |  |  |  |  |  |  |  |  |  |  |
| **T0** | 3830.861 | 6249.608 | 2714.912 | 2765.256 | 2289.677 | 2885.520 |  |  |  |  |  |  |  |  |  |  |  |  |  |  |  |  |  |  |  |  |  |  |  |  |  |  |  |  |  |  |  |  |  |  |  |  |
| **T1** | 4863.317 | 7210.253 | 4179.821 | 4470.948 | 3651.745 | 4538.882 |  |  |  |  |  |  |  |  |  |  |  |  |  |  |  |  |  |  |  |  |  |  |  |  |  |  |  |  |  |  |  |  |  |  |  |  |
| **T2** | 6787.993 | 7901.324 | 5650.810 | 6341.251 | 4657.436 | 6013.955 |  |  |  |  |  |  |  |  |  |  |  |  |  |  |  |  |  |  |  |  |  |  |  |  |  |  |  |  |  |  |  |  |  |  |  |  |
| **T3** | 7476.669 | 8818.667 | 6372.005 | 7155.950 | 6231.041 | 6779.344 |  |  |  |  |  |  |  |  |  |  |  |  |  |  |  |  |  |  |  |  |  |  |  |  |  |  |  |  |  |  |  |  |  |  |  |  |
|  |  |  |  |  |  |  |  |  |  |  |  |  |  |  |  |  |  |  |  |  |  |  |  |  |  |  |  |  |  |  |  |  |  |  |  |  |  |  |  |  |  |  |
|  |  |  |  |  |  |  |  |  |  |  |  |  |  |  |  |  |  |  |  |  |  |  |  |  |  |  |  |  |  |  |  |  |  |  |  |  |  |  |  |  |  |  |
|  |  |  |  |  |  |  |  |  |  |  |  |  |  |  |  |  |  |  |  |  |  |  |  |  |  |  |  |  |  |  |  |  |  |  |  |  |  |  |  |  |  |  |
| **Specific leaf width** | **Leaf 1** | **Leaf 2** | **Leaf 3** | **Leaf 4** | **Leaf 5** | **Leaf 6** | **Leaf 7** | **Leaf 8** | **Leaf 9** |  |  |  |  |  |  |  |  |  |  |  |  |  |  |  |  |  |  |  |  |  |  |  |  |  |  |  |  |  |  |  |  |  |
| **T0** | 62.05 | 44.23 | 83.31 | 58.14 | 71.18 | 0.00 | 0.00 | 0.00 | 0.00 |  |  |  |  |  |  |  |  |  |  |  |  |  |  |  |  |  |  |  |  |  |  |  |  |  |  |  |  |  |  |  |  |  |
| **T1** | 63.39 | 99.17 | 95.99 | 53.14 | 76.58 | 91.67 | 25.70 | 0.00 | 0.00 |  |  |  |  |  |  |  |  |  |  |  |  |  |  |  |  |  |  |  |  |  |  |  |  |  |  |  |  |  |  |  |  |  |
| **T2** | 62.95 | 117.45 | 102.10 | 57.92 | 81.73 | 130.46 | 102.38 | 0.00 | 0.00 |  |  |  |  |  |  |  |  |  |  |  |  |  |  |  |  |  |  |  |  |  |  |  |  |  |  |  |  |  |  |  |  |  |
| **T3** | 63.47 | 131.51 | 108.18 | 59.42 | 84.68 | 144.25 | 116.64 | 94.56 | 65.20 |  |  |  |  |  |  |  |  |  |  |  |  |  |  |  |  |  |  |  |  |  |  |  |  |  |  |  |  |  |  |  |  |  |
|  |  |  |  |  |  |  |  |  |  |  |  |  |  |  |  |  |  |  |  |  |  |  |  |  |  |  |  |  |  |  |  |  |  |  |  |  |  |  |  |  |  |  |
|  |  |  |  |  |  |  |  |  |  |  |  |  |  |  |  |  |  |  |  |  |  |  |  |  |  |  |  |  |  |  |  |  |  |  |  |  |  |  |  |  |  |  |
|  |  |  |  |  |  |  |  |  |  |  |  |  |  |  |  |  |  |  |  |  |  |  |  |  |  |  |  |  |  |  |  |  |  |  |  |  |  |  |  |  |  |  |
|  |  |  |  |  |  |  |  |  |  |  |  |  |  |  |  |  |  |  |  |  |  |  |  |  |  |  |  |  |  |  |  |  |  |  |  |  |  |  |  |  |  |  |
|  | | | | | | | | |  |  |  |  |  |  |  |  |  |  |  |  |  |  |  |  |  |  |  |  |  |  |  |  |  |  |  |  |  |  |  |  |  |  |
|  |  |  |  |  |  |  |  |  |  |  |  |  |  |  |  |  |  |  |  |  |  |  |  |  |  |  |  |  |  |  |  |  |  |
|  |  |  |  |  |  |  |  |  |  |  |  |  |  |  |  |  |  |  |  |  |  |  |  |  |  |  |  |  |  |  |  |  |  |
|  |  |  |  |  |  |  |  |  |  |  |  |  |  |  |  |  |  |  |  |  |  |  |  |  |  |  |  |  |  |  |  |  |  |
|  |  |  |  |  |  |  |  |  |  |  |  |  |  |  |  |  |  |  |  |  |  |  |  |  |  |  |  |  |  |  |  |  |  |
|  |  |  |  |  |  |  |  |  |  |  |  |  |  |  |  |  |  |  |  |  |  |  |  |  |  |  |  |  |  |  |  |  |  |
|  |  |  |  |  |  |  |  |  |  |  |  |  |  |  |  |  |  |  |  |  |  |  |  |  |  |  |  |  |  |  |  |  |  |
|  |  |  |  |  |  |  |  |  |  |  |  |  |  |  |  |  |  |  |  |  |  |  |  |  |  |  |  |  |  |  |  |  |  |
|  |  |  |  |  |  |  |  |  |  |  |  |  |  |  |  |  |  |  |  |  |  |  |  |  |  |  |  |  |  |  |  |  |  |
|  |  |  |  |  |  |  |  |  |  |  |  |  |  |  |  |  |  |  |  |  |  |  |  |  |  |  |  |  |  |  |  |  |  |
|  |  |  |  |  |  |  |  |  |  |  |  |  |  |  |  |  |  |  |  |  |  |  |  |  |  |  |  |  |  |  |  |  |  |
|  |  |  |  |  |  |  |  |  |  |  |  |  |  |  |  |  |  |  |  |  |  |  |  |  |  |  |  |  |  |  |  |  |  |
|  |  |  |  |  |  |  |  |  |  |  |  |  |  |  |  |  |  |  |  |  |  |  |  |  |  |  |  |  |  |  |  |  |  |
|  |  |  |  |  |  |  |  |  |  |  |  |  |  |  |  |  |  |  |  |  |  |  |  |  |  |  |  |  |  |  |  |  |  |
|  |  |  |  |  |  |  |  |  |  |  |  |  |  |  |  |  |  |  |  |  |  |  |  |  |  |  |  |  |  |  |  |  |  |
|  |  |  |  |  |  |  |  |  |  |  |  |  |  |  |  |  |  |  |  |  |  |  |  |  |  |  |  |  |  |  |  |  |  |
|  |  |  |  |  |  |  |  |  |  |  |  |  |  |  |  |  |  |  |  |  |  |  |  |  |  |  |  |  |  |  |  |  |  |
|  |  |  |  |  |  |  |  |  |  |  |  |  |  |  |  |  |  |  |  |  |  |  |  |  |  |  |  |  |  |  |  |  |  |
|  |  |  |  |  |  |  |  |  |  |  |  |  |  |  |  |  |  |  |  |  |  |  |  |  |  |  |  |  |  |  |  |  |  |
|  |  |  |  |  |  |  |  |  |  |  |  |  |  |  |  |  |  |  |  |  |  |  |  |  |  |  |  |  |  |  |  |  |  |
|  |  |  |  |  |  |  |  |  |  |  |  |  |  |  |  |  |  |  |  |  |  |  |  |  |  |  |  |  |  |  |  |  |  |
|  |  |  |  |  |  |  |  |  |  |  |  |  |  |  |  |  |  |  |  |  |  |  |  |  |  |  |  |  |  |  |  |  |  |
|  |  |  |  |  |  |  |  |  |  |  |  |  |  |  |  |  |  |  |  |  |  |  |  |  |  |  |  |  |  |  |  |  |  |
|  |  |  |  |  |  |  |  |  |  |  |  |  |  |  |  |  |  |  |  |  |  |  |  |  |  |  |  |  |  |  |  |  |  |
|  |  |  |  |  |  |  |  |  |  |  |  |  |  |  |  |  |  |  |  |  |  |  |  |  |  |  |  |  |  |  |  |  |  |
|  |  |  |  |  |  |  |  |  |  |  |  |  |  |  |  |  |  |  |  |  |  |  |  |  |  |  |  |  |  |  |  |  |  |
|  |  |  |  |  |  |  |  |  |  |  |  |  |  |  |  |  |  |  |  |  |  |  |  |  |  |  |  |  |  |  |  |  |  |
|  |  |  |  |  |  |  |  |  |  |  |  |  |  |  |  |  |  |  |  |  |  |  |  |  |  |  |  |  |  |  |  |  |  |
|  |  |  |  |  |  |  |  |  |  |  |  |  |  |  |  |  |  |  |  |  |  |  |  |  |  |  |  |  |  |  |  |  |  |
|  |  |  |  |  |  |  |  |  |  |  |  |  |  |  |  |  |  |  |  |  |  |  |  |  |  |  |  |  |  |  |  |  |  |
|  |  |  |  |  |  |  |  |  |  |  |  |  |  |  |  |  |  |  |  |  |  |  |  |  |  |  |  |  |  |  |  |  |  |
|  |  |  |  |  |  |  |  |  |  |  |  |  |  |  |  |  |  |  |  |  |  |  |  |  |  |  |  |  |  |  |  |  |  |
|  |  |  |  |  |  |  |  |  |  |  |  |  |  |  |  |  |  |  |  |  |  |  |  |  |  |  |  |  |  |  |  |  |  |
|  |  |  |  |  |  |  |  |  |  |  |  |  |  |  |  |  |  |  |  |  |  |  |  |  |  |  |  |  |  |  |  |  |  |
|  |  |  |  |  |  |  |  |  |  |  |  |  |  |  |  |  |  |  |  |  |  |  |  |  |  |  |  |  |  |  |  |  |  |
|  |  |  |  |  |  |  |  |  |  |  |  |  |  |  |  |  |  |  |  |  |  |  |  |  |  |  |  |  |  |  |  |  |  |
|  |  |  |  |  |  |  |  |  |  |  |  |  |  |  |  |  |  |  |  |  |  |  |  |  |  |  |  |  |  |  |  |  |  |
|  |  |  |  |  |  |  |  |  |  |  |  |  |  |  |  |  |  |  |  |  |  |  |  |  |  |  |  |  |  |  |  |  |  |
|  |  |  |  |  |  |  |  |  |  |  |  |  |  |  |  |  |  |  |  |  |  |  |  |  |  |  |  |  |  |  |  |  |  |
|  |  |  |  |  |  |  |  |  |  |  |  |  |  |  |  |  |  |  |  |  |  |  |  |  |  |  |  |  |  |  |  |  |  |
|  |  |  |  |  |  |  |  |  |  |  |  |  |  |  |  |  |  |  |  |  |  |  |  |  |  |  |  |  |  |  |  |  |  |
|  |  |  |  |  |  |  |  |  |  |  |  |  |  |  |  |  |  |  |  |  |  |  |  |  |  |  |  |  |  |  |  |  |  |
|  |  |  |  |  |  |  |  |  |  |  |  |  |  |  |  |  |  |  |  |  |  |  |  |  |  |  |  |  |  |  |  |  |  |
|  |  |  |  |  |  |  |  |  |  |  |  |  |  |  |  |  |  |  |  |  |  |  |  |  |  |  |  |  |  |  |  |  |  |
|  |  |  |  |  |  |  |  |  |  |  |  |  |  |  |  |  |  |  |  |  |  |  |  |  |  |  |  |  |  |  |  |  |  |
|  |  |  |  |  |  |  |  |  |  |  |  |  |  |  |  |  |  |  |  |  |  |  |  |  |  |  |  |  |  |  |  |  |  |
|  |  |  |  |  |  |  |  |  |  |  |  |  |  |  |  |  |  |  |  |  |  |  |  |  |  |  |  |  |  |  |  |  |  |
|  |  |  |  |  |  |  |  |  |  |  |  |  |  |  |  |  |  |  |  |  |  |  |  |  |  |  |  |  |  |  |  |  |  |
|  |  |  |  |  |  |  |  |  |  |  |  |  |  |  |  |  |  |  |  |  |  |  |  |  |  |  |  |  |  |  |  |  |  |
|  |  |  |  |  |  |  |  |  |  |  |  |  |  |  |  |  |  |  |  |  |  |  |  |  |  |  |  |  |  |  |  |  |  |
|  |  |  |  |  |  |  |  |  |  |  |  |  |  |  |  |  |  |  |  |  |  |  |  |  |  |  |  |  |  |  |  |  |  |
|  |  |  |  |  |  |  |  |  |  |  |  |  |  |  |  |  |  |  |  |  |  |  |  |  |  |  |  |  |  |  |  |  |  |
|  |  |  |  |  |  |  |  |  |  |  |  |  |  |  |  |  |  |  |  |  |  |  |  |  |  |  |  |  |  |  |  |  |  |  |  |  |  |  |  |  |  |  |
|  |  |  |  |  |  |  |  |  |  |  |  |  |  |  |  |  |  |  |  |  |  |  |  |  |  |  |  |  |  |  |  |  |  |  |  |  |  |  |  |  |  |  |
|  |  |  |  |  |  |  |  |  |  |  |  |  |  |  |  |  |  |  |  |  |  |  |  |  |  |  |  |  |  |  |  |  |  |  |  |  |  |  |  |  |  |  |
|  |  |  |  |  |  |  |  |  |  |  |  |  |  |  |  |  |  |  |  |  |  |  |  |  |  |  |  |  |  |  |  |  |  |  |  |  |  |  |  |  |  |  |
|  |  |  |  |  |  |  |  |  |  |  |  |  |  |  |  |  |  |  |  |  |  |  |  |  |  |  |  |  |  |  |  |  |  |  |  |  |  |  |  |  |  |  |
|  | | | | | | | | |  |  |  |  |  |  |  |  |  |  |  |  |  |  |  |  |  |  |  |  |  |  |  |  |  |  |  |  |  |  |  |  |  |  |
|  |  |  |  |  |  |  |  |  |  |  |  |  |  |  |  |  |  |  |  |  |  |  |  |  |  |  |  |  |  |  |  |  |  |
|  |  |  |  |  |  |  |  |  |  |  |  |  |  |  |  |  |  |  |  |  |  |  |  |  |  |  |  |  |  |  |  |  |  |
|  |  |  |  |  |  |  |  |  |  |  |  |  |  |  |  |  |  |  |  |  |  |  |  |  |  |  |  |  |  |  |  |  |  |
|  |  |  |  |  |  |  |  |  |  |  |  |  |  |  |  |  |  |  |  |  |  |  |  |  |  |  |  |  |  |  |  |  |  |
|  |  |  |  |  |  |  |  |  |  |  |  |  |  |  |  |  |  |  |  |  |  |  |  |  |  |  |  |  |  |  |  |  |  |
|  |  |  |  |  |  |  |  |  |  |  |  |  |  |  |  |  |  |  |  |  |  |  |  |  |  |  |  |  |  |  |  |  |  |
|  |  |  |  |  |  |  |  |  |  |  |  |  |  |  |  |  |  |  |  |  |  |  |  |  |  |  |  |  |  |  |  |  |  |
|  |  |  |  |  |  |  |  |  |  |  |  |  |  |  |  |  |  |  |  |  |  |  |  |  |  |  |  |  |  |  |  |  |  |
|  |  |  |  |  |  |  |  |  |  |  |  |  |  |  |  |  |  |  |  |  |  |  |  |  |  |  |  |  |  |  |  |  |  |
|  |  |  |  |  |  |  |  |  |  |  |  |  |  |  |  |  |  |  |  |  |  |  |  |  |  |  |  |  |  |  |  |  |  |
|  |  |  |  |  |  |  |  |  |  |  |  |  |  |  |  |  |  |  |  |  |  |  |  |  |  |  |  |  |  |  |  |  |  |
|  |  |  |  |  |  |  |  |  |  |  |  |  |  |  |  |  |  |  |  |  |  |  |  |  |  |  |  |  |  |  |  |  |  |
|  |  |  |  |  |  |  |  |  |  |  |  |  |  |  |  |  |  |  |  |  |  |  |  |  |  |  |  |  |  |  |  |  |  |
|  |  |  |  |  |  |  |  |  |  |  |  |  |  |  |  |  |  |  |  |  |  |  |  |  |  |  |  |  |  |  |  |  |  |
|  |  |  |  |  |  |  |  |  |  |  |  |  |  |  |  |  |  |  |  |  |  |  |  |  |  |  |  |  |  |  |  |  |  |
|  |  |  |  |  |  |  |  |  |  |  |  |  |  |  |  |  |  |  |  |  |  |  |  |  |  |  |  |  |  |  |  |  |  |
|  |  |  |  |  |  |  |  |  |  |  |  |  |  |  |  |  |  |  |  |  |  |  |  |  |  |  |  |  |  |  |  |  |  |
|  |  |  |  |  |  |  |  |  |  |  |  |  |  |  |  |  |  |  |  |  |  |  |  |  |  |  |  |  |  |  |  |  |  |
|  |  |  |  |  |  |  |  |  |  |  |  |  |  |  |  |  |  |  |  |  |  |  |  |  |  |  |  |  |  |  |  |  |  |
|  |  |  |  |  |  |  |  |  |  |  |  |  |  |  |  |  |  |  |  |  |  |  |  |  |  |  |  |  |  |  |  |  |  |
|  |  |  |  |  |  |  |  |  |  |  |  |  |  |  |  |  |  |  |  |  |  |  |  |  |  |  |  |  |  |  |  |  |  |
|  |  |  |  |  |  |  |  |  |  |  |  |  |  |  |  |  |  |  |  |  |  |  |  |  |  |  |  |  |  |  |  |  |  |
|  |  |  |  |  |  |  |  |  |  |  |  |  |  |  |  |  |  |  |  |  |  |  |  |  |  |  |  |  |  |  |  |  |  |
|  |  |  |  |  |  |  |  |  |  |  |  |  |  |  |  |  |  |  |  |  |  |  |  |  |  |  |  |  |  |  |  |  |  |
|  |  |  |  |  |  |  |  |  |  |  |  |  |  |  |  |  |  |  |  |  |  |  |  |  |  |  |  |  |  |  |  |  |  |
|  |  |  |  |  |  |  |  |  |  |  |  |  |  |  |  |  |  |  |  |  |  |  |  |  |  |  |  |  |  |  |  |  |  |
|  |  |  |  |  |  |  |  |  |  |  |  |  |  |  |  |  |  |  |  |  |  |  |  |  |  |  |  |  |  |  |  |  |  |
|  |  |  |  |  |  |  |  |  |  |  |  |  |  |  |  |  |  |  |  |  |  |  |  |  |  |  |  |  |  |  |  |  |  |
|  |  |  |  |  |  |  |  |  |  |  |  |  |  |  |  |  |  |  |  |  |  |  |  |  |  |  |  |  |  |  |  |  |  |
|  |  |  |  |  |  |  |  |  |  |  |  |  |  |  |  |  |  |  |  |  |  |  |  |  |  |  |  |  |  |  |  |  |  |
|  |  |  |  |  |  |  |  |  |  |  |  |  |  |  |  |  |  |  |  |  |  |  |  |  |  |  |  |  |  |  |  |  |  |
|  |  |  |  |  |  |  |  |  |  |  |  |  |  |  |  |  |  |  |  |  |  |  |  |  |  |  |  |  |  |  |  |  |  |
|  |  |  |  |  |  |  |  |  |  |  |  |  |  |  |  |  |  |  |  |  |  |  |  |  |  |  |  |  |  |  |  |  |  |
|  |  |  |  |  |  |  |  |  |  |  |  |  |  |  |  |  |  |  |  |  |  |  |  |  |  |  |  |  |  |  |  |  |  |
|  |  |  |  |  |  |  |  |  |  |  |  |  |  |  |  |  |  |  |  |  |  |  |  |  |  |  |  |  |  |  |  |  |  |
|  |  |  |  |  |  |  |  |  |  |  |  |  |  |  |  |  |  |  |  |  |  |  |  |  |  |  |  |  |  |  |  |  |  |
|  |  |  |  |  |  |  |  |  |  |  |  |  |  |  |  |  |  |  |  |  |  |  |  |  |  |  |  |  |  |  |  |  |  |
|  |  |  |  |  |  |  |  |  |  |  |  |  |  |  |  |  |  |  |  |  |  |  |  |  |  |  |  |  |  |  |  |  |  |
|  |  |  |  |  |  |  |  |  |  |  |  |  |  |  |  |  |  |  |  |  |  |  |  |  |  |  |  |  |  |  |  |  |  |
|  |  |  |  |  |  |  |  |  |  |  |  |  |  |  |  |  |  |  |  |  |  |  |  |  |  |  |  |  |  |  |  |  |  |
|  |  |  |  |  |  |  |  |  |  |  |  |  |  |  |  |  |  |  |  |  |  |  |  |  |  |  |  |  |  |  |  |  |  |
|  |  |  |  |  |  |  |  |  |  |  |  |  |  |  |  |  |  |  |  |  |  |  |  |  |  |  |  |  |  |  |  |  |  |
|  |  |  |  |  |  |  |  |  |  |  |  |  |  |  |  |  |  |  |  |  |  |  |  |  |  |  |  |  |  |  |  |  |  |
|  |  |  |  |  |  |  |  |  |  |  |  |  |  |  |  |  |  |  |  |  |  |  |  |  |  |  |  |  |  |  |  |  |  |
|  |  |  |  |  |  |  |  |  |  |  |  |  |  |  |  |  |  |  |  |  |  |  |  |  |  |  |  |  |  |  |  |  |  |
|  |  |  |  |  |  |  |  |  |  |  |  |  |  |  |  |  |  |  |  |  |  |  |  |  |  |  |  |  |  |  |  |  |  |
|  |  |  |  |  |  |  |  |  |  |  |  |  |  |  |  |  |  |  |  |  |  |  |  |  |  |  |  |  |  |  |  |  |  |
|  |  |  |  |  |  |  |  |  |  |  |  |  |  |  |  |  |  |  |  |  |  |  |  |  |  |  |  |  |  |  |  |  |  |
|  |  |  |  |  |  |  |  |  |  |  |  |  |  |  |  |  |  |  |  |  |  |  |  |  |  |  |  |  |  |  |  |  |  |
|  |  |  |  |  |  |  |  |  |  |  |  |  |  |  |  |  |  |  |  |  |  |  |  |  |  |  |  |  |  |  |  |  |  |
|  |  |  |  |  |  |  |  |  |  |  |  |  |  |  |  |  |  |  |  |  |  |  |  |  |  |  |  |  |  |  |  |  |  |  |  |  |  |  |  |  |  |  |
|  |  |  |  |  |  |  |  |  |  |  |  |  |  |  |  |  |  |  |  |  |  |  |  |  |  |  |  |  |  |  |  |  |  |  |  |  |  |  |  |  |  |  |
|  |  |  |  |  |  |  |  |  |  |  |  |  |  |  |  |  |  |  |  |  |  |  |  |  |  |  |  |  |  |  |  |  |  |  |  |  |  |  |  |  |  |  |
|  |  |  |  |  |  |  |  |  |  |  |  |  |  |  |  |  |  |  |  |  |  |  |  |  |  |  |  |  |  |  |  |  |  |  |  |  |  |  |  |  |  |  |
|  |  |  |  |  |  |  |  |  |  |  |  |  |  |  |  |  |  |  |  |  |  |  |  |  |  |  |  |  |  |  |  |  |  |  |  |  |  |  |  |  |  |  |
|  |  |  |  |  |  |  |  |  |  |  |  |  |  |  |  |  |  |  |  |  |  |  |  |  |  |  |  |  |  |  |  |  |  |  |  |  |  |  |  |  |  |  |
|  |  |  |  |  |  |  |  |  |  |  |  |  |  |  |  |  |  |  |  |  |  |  |  |  |  |  |  |  |  |  |  |  |  |  |  |  |  |  |  |  |  |  |
|  |  |  |  |  |  |  |  |  |  |  |  |  |  |  |  |  |  |  |  |  |  |  |  |  |  |  |  |  |  |  |  |  |  |  |  |  |  |  |  |  |  |  |
|  |  |  |  |  |  |  |  |  |  |  |  |  |  |  |  |  |  |  |  |  |  |  |  |  |  |  |  |  |  |  |  |  |  |  |  |  |  |  |  |  |  |  |
|  | | | | | | | | |  |  |  |  |  |  |  |  |  |  |  |  |  |  |  |  |  |  |  |  |  |  |  |  |  |  |  |  |  |  |  |  |  |  |
|  |  |  |  |  |  |  |  |  |  |  |  |  |  |  |  |  |  |  |  |  |  |  |  |  |  |  |  |  |  |  |  |  |  |
|  |  |  |  |  |  |  |  |  |  |  |  |  |  |  |  |  |  |  |  |  |  |  |  |  |  |  |  |  |  |  |  |  |  |
|  |  |  |  |  |  |  |  |  |  |  |  |  |  |  |  |  |  |  |  |  |  |  |  |  |  |  |  |  |  |  |  |  |  |
|  |  |  |  |  |  |  |  |  |  |  |  |  |  |  |  |  |  |  |  |  |  |  |  |  |  |  |  |  |  |  |  |  |  |
|  |  |  |  |  |  |  |  |  |  |  |  |  |  |  |  |  |  |  |  |  |  |  |  |  |  |  |  |  |  |  |  |  |  |
|  |  |  |  |  |  |  |  |  |  |  |  |  |  |  |  |  |  |  |  |  |  |  |  |  |  |  |  |  |  |  |  |  |  |
|  |  |  |  |  |  |  |  |  |  |  |  |  |  |  |  |  |  |  |  |  |  |  |  |  |  |  |  |  |  |  |  |  |  |
|  |  |  |  |  |  |  |  |  |  |  |  |  |  |  |  |  |  |  |  |  |  |  |  |  |  |  |  |  |  |  |  |  |  |
|  |  |  |  |  |  |  |  |  |  |  |  |  |  |  |  |  |  |  |  |  |  |  |  |  |  |  |  |  |  |  |  |  |  |
|  |  |  |  |  |  |  |  |  |  |  |  |  |  |  |  |  |  |  |  |  |  |  |  |  |  |  |  |  |  |  |  |  |  |
|  |  |  |  |  |  |  |  |  |  |  |  |  |  |  |  |  |  |  |  |  |  |  |  |  |  |  |  |  |  |  |  |  |  |
|  |  |  |  |  |  |  |  |  |  |  |  |  |  |  |  |  |  |  |  |  |  |  |  |  |  |  |  |  |  |  |  |  |  |
|  |  |  |  |  |  |  |  |  |  |  |  |  |  |  |  |  |  |  |  |  |  |  |  |  |  |  |  |  |  |  |  |  |  |
|  |  |  |  |  |  |  |  |  |  |  |  |  |  |  |  |  |  |  |  |  |  |  |  |  |  |  |  |  |  |  |  |  |  |
|  |  |  |  |  |  |  |  |  |  |  |  |  |  |  |  |  |  |  |  |  |  |  |  |  |  |  |  |  |  |  |  |  |  |
|  |  |  |  |  |  |  |  |  |  |  |  |  |  |  |  |  |  |  |  |  |  |  |  |  |  |  |  |  |  |  |  |  |  |
|  |  |  |  |  |  |  |  |  |  |  |  |  |  |  |  |  |  |  |  |  |  |  |  |  |  |  |  |  |  |  |  |  |  |
|  |  |  |  |  |  |  |  |  |  |  |  |  |  |  |  |  |  |  |  |  |  |  |  |  |  |  |  |  |  |  |  |  |  |
|  |  |  |  |  |  |  |  |  |  |  |  |  |  |  |  |  |  |  |  |  |  |  |  |  |  |  |  |  |  |  |  |  |  |
|  |  |  |  |  |  |  |  |  |  |  |  |  |  |  |  |  |  |  |  |  |  |  |  |  |  |  |  |  |  |  |  |  |  |
|  |  |  |  |  |  |  |  |  |  |  |  |  |  |  |  |  |  |  |  |  |  |  |  |  |  |  |  |  |  |  |  |  |  |
|  |  |  |  |  |  |  |  |  |  |  |  |  |  |  |  |  |  |  |  |  |  |  |  |  |  |  |  |  |  |  |  |  |  |
|  |  |  |  |  |  |  |  |  |  |  |  |  |  |  |  |  |  |  |  |  |  |  |  |  |  |  |  |  |  |  |  |  |  |
|  |  |  |  |  |  |  |  |  |  |  |  |  |  |  |  |  |  |  |  |  |  |  |  |  |  |  |  |  |  |  |  |  |  |
|  |  |  |  |  |  |  |  |  |  |  |  |  |  |  |  |  |  |  |  |  |  |  |  |  |  |  |  |  |  |  |  |  |  |
|  |  |  |  |  |  |  |  |  |  |  |  |  |  |  |  |  |  |  |  |  |  |  |  |  |  |  |  |  |  |  |  |  |  |
|  |  |  |  |  |  |  |  |  |  |  |  |  |  |  |  |  |  |  |  |  |  |  |  |  |  |  |  |  |  |  |  |  |  |
|  |  |  |  |  |  |  |  |  |  |  |  |  |  |  |  |  |  |  |  |  |  |  |  |  |  |  |  |  |  |  |  |  |  |
|  |  |  |  |  |  |  |  |  |  |  |  |  |  |  |  |  |  |  |  |  |  |  |  |  |  |  |  |  |  |  |  |  |  |
|  |  |  |  |  |  |  |  |  |  |  |  |  |  |  |  |  |  |  |  |  |  |  |  |  |  |  |  |  |  |  |  |  |  |
|  |  |  |  |  |  |  |  |  |  |  |  |  |  |  |  |  |  |  |  |  |  |  |  |  |  |  |  |  |  |  |  |  |  |
|  |  |  |  |  |  |  |  |  |  |  |  |  |  |  |  |  |  |  |  |  |  |  |  |  |  |  |  |  |  |  |  |  |  |
|  |  |  |  |  |  |  |  |  |  |  |  |  |  |  |  |  |  |  |  |  |  |  |  |  |  |  |  |  |  |  |  |  |  |
|  |  |  |  |  |  |  |  |  |  |  |  |  |  |  |  |  |  |  |  |  |  |  |  |  |  |  |  |  |  |  |  |  |  |
|  |  |  |  |  |  |  |  |  |  |  |  |  |  |  |  |  |  |  |  |  |  |  |  |  |  |  |  |  |  |  |  |  |  |
|  |  |  |  |  |  |  |  |  |  |  |  |  |  |  |  |  |  |  |  |  |  |  |  |  |  |  |  |  |  |  |  |  |  |
|  |  |  |  |  |  |  |  |  |  |  |  |  |  |  |  |  |  |  |  |  |  |  |  |  |  |  |  |  |  |  |  |  |  |
|  |  |  |  |  |  |  |  |  |  |  |  |  |  |  |  |  |  |  |  |  |  |  |  |  |  |  |  |  |  |  |  |  |  |
|  |  |  |  |  |  |  |  |  |  |  |  |  |  |  |  |  |  |  |  |  |  |  |  |  |  |  |  |  |  |  |  |  |  |
|  |  |  |  |  |  |  |  |  |  |  |  |  |  |  |  |  |  |  |  |  |  |  |  |  |  |  |  |  |  |  |  |  |  |
|  |  |  |  |  |  |  |  |  |  |  |  |  |  |  |  |  |  |  |  |  |  |  |  |  |  |  |  |  |  |  |  |  |  |
|  |  |  |  |  |  |  |  |  |  |  |  |  |  |  |  |  |  |  |  |  |  |  |  |  |  |  |  |  |  |  |  |  |  |
|  |  |  |  |  |  |  |  |  |  |  |  |  |  |  |  |  |  |  |  |  |  |  |  |  |  |  |  |  |  |  |  |  |  |
|  |  |  |  |  |  |  |  |  |  |  |  |  |  |  |  |  |  |  |  |  |  |  |  |  |  |  |  |  |  |  |  |  |  |
|  |  |  |  |  |  |  |  |  |  |  |  |  |  |  |  |  |  |  |  |  |  |  |  |  |  |  |  |  |  |  |  |  |  |
|  |  |  |  |  |  |  |  |  |  |  |  |  |  |  |  |  |  |  |  |  |  |  |  |  |  |  |  |  |  |  |  |  |  |
|  |  |  |  |  |  |  |  |  |  |  |  |  |  |  |  |  |  |  |  |  |  |  |  |  |  |  |  |  |  |  |  |  |  |
|  |  |  |  |  |  |  |  |  |  |  |  |  |  |  |  |  |  |  |  |  |  |  |  |  |  |  |  |  |  |  |  |  |  |
|  |  |  |  |  |  |  |  |  |  |  |  |  |  |  |  |  |  |  |  |  |  |  |  |  |  |  |  |  |  |  |  |  |  |
|  |  |  |  |  |  |  |  |  |  |  |  |  |  |  |  |  |  |  |  |  |  |  |  |  |  |  |  |  |  |  |  |  |  |
|  |  |  |  |  |  |  |  |  |  |  |  |  |  |  |  |  |  |  |  |  |  |  |  |  |  |  |  |  |  |  |  |  |  |
|  |  |  |  |  |  |  |  |  |  |  |  |  |  |  |  |  |  |  |  |  |  |  |  |  |  |  |  |  |  |  |  |  |  |
|  |  |  |  |  |  |  |  |  |  |  |  |  |  |  |  |  |  |  |  |  |  |  |  |  |  |  |  |  |  |  |  |  |  |
|  |  |  |  |  |  |  |  |  |  |  |  |  |  |  |  |  |  |  |  |  |  |  |  |  |  |  |  |  |  |  |  |  |  |
|  |  |  |  |  |  |  |  |  |  |  |  |  |  |  |  |  |  |  |  |  |  |  |  |  |  |  |  |  |  |  |  |  |  |
|  |  |  |  |  |  |  |  |  |  |  |  |  |  |  |  |  |  |  |  |  |  |  |  |  |  |  |  |  |  |  |  |  |  |
|  |  |  |  |  |  |  |  |  |  |  |  |  |  |  |  |  |  |  |  |  |  |  |  |  |  |  |  |  |  |  |  |  |  |
|  |  |  |  |  |  |  |  |  |  |  |  |  |  |  |  |  |  |  |  |  |  |  |  |  |  |  |  |  |  |  |  |  |  |
|  |  |  |  |  |  |  |  |  |  |  |  |  |  |  |  |  |  |  |  |  |  |  |  |  |  |  |  |  |  |  |  |  |  |
|  |  |  |  |  |  |  |  |  |  |  |  |  |  |  |  |  |  |  |  |  |  |  |  |  |  |  |  |  |  |  |  |  |  |
|  |  |  |  |  |  |  |  |  |  |  |  |  |  |  |  |  |  |  |  |  |  |  |  |  |  |  |  |  |  |  |  |  |  |
|  |  |  |  |  |  |  |  |  |  |  |  |  |  |  |  |  |  |  |  |  |  |  |  |  |  |  |  |  |  |  |  |  |  |
|  |  |  |  |  |  |  |  |  |  |  |  |  |  |  |  |  |  |  |  |  |  |  |  |  |  |  |  |  |  |  |  |  |  |
|  |  |  |  |  |  |  |  |  |  |  |  |  |  |  |  |  |  |  |  |  |  |  |  |  |  |  |  |  |  |  |  |  |  |
|  |  |  |  |  |  |  |  |  |  |  |  |  |  |  |  |  |  |  |  |  |  |  |  |  |  |  |  |  |  |  |  |  |  |
|  |  |  |  |  |  |  |  |  |  |  |  |  |  |  |  |  |  |  |  |  |  |  |  |  |  |  |  |  |  |  |  |  |  |
|  |  |  |  |  |  |  |  |  |  |  |  |  |  |  |  |  |  |  |  |  |  |  |  |  |  |  |  |  |  |  |  |  |  |
|  |  |  |  |  |  |  |  |  |  |  |  |  |  |  |  |  |  |  |  |  |  |  |  |  |  |  |  |  |  |  |  |  |  |
|  |  |  |  |  |  |  |  |  |  |  |  |  |  |  |  |  |  |  |  |  |  |  |  |  |  |  |  |  |  |  |  |  |  |
|  |  |  |  |  |  |  |  |  |  |  |  |  |  |  |  |  |  |  |  |  |  |  |  |  |  |  |  |  |  |  |  |  |  |
