# Supplementary material for: A novel mesh processing based technique for 3D plant analysis
Source: BMC Plant Biol. 2012 May 3;12:63. doi: 10.1186/1471-2229-12-63 (PMC3464618; doi:10.1186/1471-2229-12-63)
Supplement: Addtional file 1 — Website presenting the results. Website containing the results obtained by applying our method on the initial set of plant meshes. The different results are presented as tables containing links to the different web-pages. The results of the segmentation and temporal matching between the different time-points are available as images. Phenotypic parameters estimated by our method are available in the form of tables. In addition, a spreadsheet containing all the mesh-based and manual measurements is available as a web-page and contains the statistical analysis presented in the paper. [file 1471-2229-12-63-S1.zip › PlantPhenomics_mini_website_bmc/webpages/Longitudinal_Plant1.html]

# A novel mesh processing based technique for 3-D plant analysis

Return to index

## Temporal analysis for Plant1

### Original data

- Plant1\_T0.vtk
- Plant1\_T1.vtk
- Plant1\_T2.vtk
- Plant1\_T3.vtk

  
  
These meshes can be visualised using Paraview

### Temporal main stem height

| Plant ID | Main Stem Height T0 | Main Stem Height T1 | Main Stem Height T2 | Main Stem Height T3 |
| --- | --- | --- | --- | --- |
| Plant1 | 142.775 | 167.752 | 222.978 | 259.328 |

### Number of leaves per time point

| Leaf ID | # leaf T0 | # leaf T1 | # leaf T2 | # leaf T3 |
| --- | --- | --- | --- | --- |
| Plant1 | 7 | 8 | 10 | 12 |

### Temporal leaves width monitoring

| Leaf ID | Leaf width T0 | Leaf width T1 | Leaf width T2 | Leaf width T3 |
| --- | --- | --- | --- | --- |
| Leaf0 | 63.944 | 65.4125 | 65.9666 | 65.1738 |
| Leaf1 | 91.292 | 117.796 | 125.63 | 129.212 |
| Leaf2 | 84.3709 | 76.5217 | 127.004 | 135.15 |
| Leaf3 | 41.9575 | 43.9156 | 39.6414 | 45.1564 |
| Leaf4 | 38.252 | 102.181 | 134.715 | 139.84 |
| Leaf5 | 83.0794 | 86.0017 | 88.813 | 88.6196 |
| Leaf6 | 59.271 | 43.7673 | 60.2804 | 61.6718 |
| Leaf7 | 0.0 | 106.184 | 119.023 | 120.939 |
| Leaf8 | 0.0 | 0.0 | 97.6879 | 123.684 |
| Leaf9 | 0.0 | 0.0 | 83.3824 | 121.271 |
| Leaf10 | 0.0 | 0.0 | 0.0 | 28.7924 |
| Leaf11 | 0.0 | 0.0 | 0.0 | 39.2311 |

### Temporal leaves length monitoring

| Leaf ID | Leaf length T0 | Leaf length T1 | Leaf length T2 | Leaf length T3 |
| --- | --- | --- | --- | --- |
| Leaf0 | 65.845 | 66.258 | 68.9257 | 67.1619 |
| Leaf1 | 93.4657 | 109.424 | 116.937 | 117.363 |
| Leaf2 | 86.0088 | 72.6402 | 122.907 | 125.891 |
| Leaf3 | 32.6725 | 30.8705 | 32.4076 | 27.8248 |
| Leaf4 | 32.3213 | 81.7902 | 121.038 | 124.296 |
| Leaf5 | 78.0491 | 77.1204 | 86.8845 | 85.3442 |
| Leaf6 | 38.9684 | 34.1963 | 39.3129 | 35.6089 |
| Leaf7 | 0.0 | 109.635 | 115.651 | 111.39 |
| Leaf8 | 0.0 | 0.0 | 92.1543 | 107.477 |
| Leaf9 | 0.0 | 0.0 | 72.6641 | 99.1692 |
| Leaf10 | 0.0 | 0.0 | 0.0 | 20.6672 |
| Leaf11 | 0.0 | 0.0 | 0.0 | 36.7142 |

### Temporal leaves area monitoring

| Leaf ID | Leaf area T0 | Leaf area T1 | Leaf area T2 | Leaf area T3 |
| --- | --- | --- | --- | --- |
| Leaf0 | 3490.9 | 3369.32 | 3648.26 | 3354.25 |
| Leaf1 | 5749.58 | 9076.88 | 11514.9 | 11209.6 |
| Leaf2 | 5341.93 | 4087.39 | 11208.7 | 12613.2 |
| Leaf3 | 1301.88 | 1103.61 | 1152.52 | 1115.23 |
| Leaf4 | 664.272 | 6284.44 | 12192.3 | 12052.3 |
| Leaf5 | 5075.1 | 5158.27 | 5811.78 | 5694.94 |
| Leaf6 | 2025.69 | 1426.63 | 1999.51 | 1897.29 |
| Leaf7 | 0.0 | 8400 | 10376 | 9747.18 |
| Leaf8 | 0.0 | 0.0 | 6108.76 | 9175.74 |
| Leaf9 | 0.0 | 0.0 | 3899.22 | 7906.99 |
| Leaf10 | 0.0 | 0.0 | 0.0 | 440.665 |
| Leaf11 | 0.0 | 0.0 | 0.0 | 914.353 |

### Illustration of the matching of the plants limbs

#### From T0 to T1

 

#### From T1 to T2

 

#### From T2 to T3

 
Return to index 
