# Supplementary material for: A novel mesh processing based technique for 3D plant analysis
Source: BMC Plant Biol. 2012 May 3;12:63. doi: 10.1186/1471-2229-12-63 (PMC3464618; doi:10.1186/1471-2229-12-63)
Supplement: Addtional file 1 — Website presenting the results. Website containing the results obtained by applying our method on the initial set of plant meshes. The different results are presented as tables containing links to the different web-pages. The results of the segmentation and temporal matching between the different time-points are available as images. Phenotypic parameters estimated by our method are available in the form of tables. In addition, a spreadsheet containing all the mesh-based and manual measurements is available as a web-page and contains the statistical analysis presented in the paper. [file 1471-2229-12-63-S1.zip › PlantPhenomics_mini_website_bmc/webpages/Plant6_T0.html]

# A novel mesh processing based technique for 3-D plant analysis

Return to index

## Results for Plant6 at T0

### Original data

Mesh available upon request to Anthony Paproki. Only the meshes for the plant 1 are available through this website due to size limitation for the BMC mini-websites

### Leaves data

| Case ID | Time Point | Leaf ID | Width | Length | Area |
| --- | --- | --- | --- | --- | --- |
| Plant6 | T0 | 0 | 84.483735 | 77.920663 | 4491.451105 |
| Plant6 | T0 | 1 | 58.751958 | 58.123213 | 3038.654072 |
| Plant6 | T0 | 2 | 57.488509 | 37.234795 | 1844.638643 |
| Plant6 | T0 | 3 | 55.760779 | 44.053503 | 1058.280455 |
| Plant6 | T0 | 4 | 88.091308 | 83.984992 | 5344.376648 |
| Plant6 | T0 | 5 | 53.611783 | 32.970494 | 1535.720418 |

### Branches data

| Case ID | Time Point | Branch ID | Length | Initiation Angle |
| --- | --- | --- | --- | --- |
| Plant6 | T0 | 0 | 41.960165 | 76.803238 |
| Plant6 | T0 | 1 | 0.550734 | 77.922803 |
| Plant6 | T0 | 2 | 28.637189 | 60.635630 |
| Plant6 | T0 | 3 | 69.461144 | 60.529726 |
| Plant6 | T0 | 4 | 26.952259 | 51.806110 |
| Plant6 | T0 | 5 | 28.612312 | 59.196061 |

### Segmentation Illustration

  Return to index 
